# Novel regional age-associated DNA methylation changes within human common disease-associated loci

**DOI:** 10.1186/s13059-016-1051-8

**Published:** 2016-09-23

**Authors:** Christopher G. Bell, Yudong Xia, Wei Yuan, Fei Gao, Kirsten Ward, Leonie Roos, Massimo Mangino, Pirro G. Hysi, Jordana Bell, Jun Wang, Timothy D. Spector

**Affiliations:** 1 Department of Twin Research & Genetic Epidemiology, King’s College London, London, UK; 2 MRC Lifecourse Epidemiology Unit, University of Southampton, Southampton, UK; 3 Human Development and Health Academic Unit, Institute of Developmental Sciences, University of Southampton, Southampton, UK; 4 Epigenomic Medicine, Biological Sciences, Faculty of Environmental and Natural Sciences, University of Southampton, Southampton, UK; 5 BGI-Shenzhen, Shenzhen, 518083 China; 6 Institute of Cancer Research, Sutton, UK

**Keywords:** Epigenetics, DNA methylation, Ageing, Common Disease, Human

## Abstract

**Background:**

Advancing age progressively impacts on risk and severity of chronic disease. It also modifies the epigenome, with changes in DNA methylation, due to both random drift and variation within specific functional loci.

**Results:**

In a discovery set of 2238 peripheral-blood genome-wide DNA methylomes aged 19–82 years, we identify 71 age-associated differentially methylated regions within the linkage disequilibrium blocks of the single nucleotide polymorphisms from the NIH genome-wide association study catalogue. This included 52 novel regions, 29 within loci not covered by 450 k or 27 k Illumina array, and with enrichment for DNase-I Hypersensitivity sites across the full range of tissues. These age-associated differentially methylated regions also show marked enrichment for enhancers and poised promoters across multiple cell types. In a replication set of 2084 DNA methylomes, 95.7 % of the age-associated differentially methylated regions showed the same direction of ageing effect, with 80.3 % and 53.5 % replicated to *p* < 0.05 and *p* < 1.85 × 10^–8^, respectively.

**Conclusion:**

By analysing the functionally enriched disease and trait-associated regions of the human genome, we identify novel epigenetic ageing changes, which could be useful biomarkers or provide mechanistic insights into age-related common diseases.

**Electronic supplementary material:**

The online version of this article (doi:10.1186/s13059-016-1051-8) contains supplementary material, which is available to authorized users.

## Background

Age is a risk factor for multiple chronic diseases. It impacts on all organ systems leading to decreased functionality and eventual death [1]. Epigenetic changes associated with the ageing process, specifically DNA methylation, have been identified in a number of studies [2–16]. While stochastic epigenetic drift does occur [17], non-random changes are seen within specific functional loci, such as increased DNA methylation in the promoters of the target genes of polycomb group proteins [3] and bivalent chromatin regions or poised promoters [4]. Additionally, recent formulation of an epigenetic ‘clock’ [9] has led to the observation that accelerated discordance between the DNA methylation estimate and actual chronological age is a risk factor for all-cause mortality in later life [18].

The ageing-associated DNA methylation signatures identified to date have almost exclusively been via targeted array based methodology (Illumina 27 k or 450 k). Notable exceptions are a study by McClay et al. performed via methyl binding domain enriched second-generation sequencing (MBD-seq) [12] and Heyn et al. comparing a single centenarian with a single newborn with whole genome bisulphite sequencing (WGBS) data [6]. Within heterogeneous peripheral blood, an increase in the myeloid fraction of blood cell sub-types is known to occur with advancing age [19] and must be accounted for in these analyses. DNA methylation changes in peripheral blood may therefore represent this myeloid skewing, biological cascades leading to upregulation or downregulation of specialised cell subtypes [20], or additional undefined active or passive changes associated with age [21].

We analysed 2238 unique DNA methylomes for changes associated with chronological age. These data were generated by genome-wide methylated DNA immunoprecipitation Illumina second-generation sequencing (MeDIP-seq) in peripheral blood. MeDIP-seq methylome results identify broader regional changes compared with targeted individual CpG array approaches and are not limited to the array-predefined cytosines. Data were included on significant influencing variables including full blood count, smoking status and batch. To account for the strong genetic effects inherent with DNA methylation analysis, especially by techniques such as MeDIP-seq [22] we incorporated common SNP genotyping data available on all these individuals.

We focused on the a priori functional regions [23], the linkage disequilibrium (LD) blocks of all of the phenotype and disease-related single-nucleotide polymorphisms (SNPs) entered in the genome-wide association study (GWAS) catalogue [24] (8093 curated GWAS SNPs with *p* value < 1 × 10^–7^ residing within 2709 distinct LD blocks, ~22.1 % of the genome). Multiple strands of evidence have now accrued from the ENCODE consortium and other regulatory datasets that the regions identified through the thousands of GWAS studies performed to date are enriched for active loci [25, 26]. We pursued this strategy to explore the GWAS LD blocks in order to identify novel epigenetic changes that were more likely to be functional and, due to their co-location, would enable direct integration into future locus-specific common disease investigations. As age impacts on the penetrance and severity of many of these common diseases and phenotypes, this analysis enabled us to specifically explore these blocks for age-related changes. This is particularly of interest because changes identified in studies, such as the DNA methylation ‘clock’ and others, can be seen across multiple tissues [9, 15]. Thus these blood-based findings could, in some cases, have impact not just in haematological or immunological disorders, but also within the most disease-relevant tissue.

The epigenetic state within a locus can be obligatory or fixed due to the underlying genetic framework or may vary, facilitated by particular sequence constructs [27]. Using haplotype-tagging common SNP data in these individuals, we could control for the obligatory genetic effects in the LD blocks. We could also subsequently investigate whether facilitative DNA methylation ageing changes differed with respect to common risk versus non-risk haplotype background within these GWAS regions. The potential of genetically facilitated ageing modifications has been explored [10, 16] and its direct assessment could add additional mechanistic insight within these disease-associated loci.

## Results

### GWAS LD block regions are functionally enriched

Multiple studies have indicated that the regions identified by GWAS are functionally enriched [25, 26]. To further demonstrate this, we explored public ENCODE data and compared the 2709 distinct GWAS LD blocks (~22.1 % of the genome) to the remainder of the genome. We focused on the DNase I Hypersensitivity site (DHS) due to their broad capacity to act as functional indicators [28]. We also explored the recently identified DNA methylation-sensitive transcription factor NRF1 [29]. We found that DHSs and NRF1 both show significant occupancy enrichment within this portion of the genome (both Fisher’s exact test *p* < 2.2 × 10^–16^, odds ratio (OR) = 1.70 and 2.26, confidence intervals (CI) 1.69–1.71 and 2.17–2.35, respectively).

### Age-associated differentially methylated regions

**Fig. 1:**
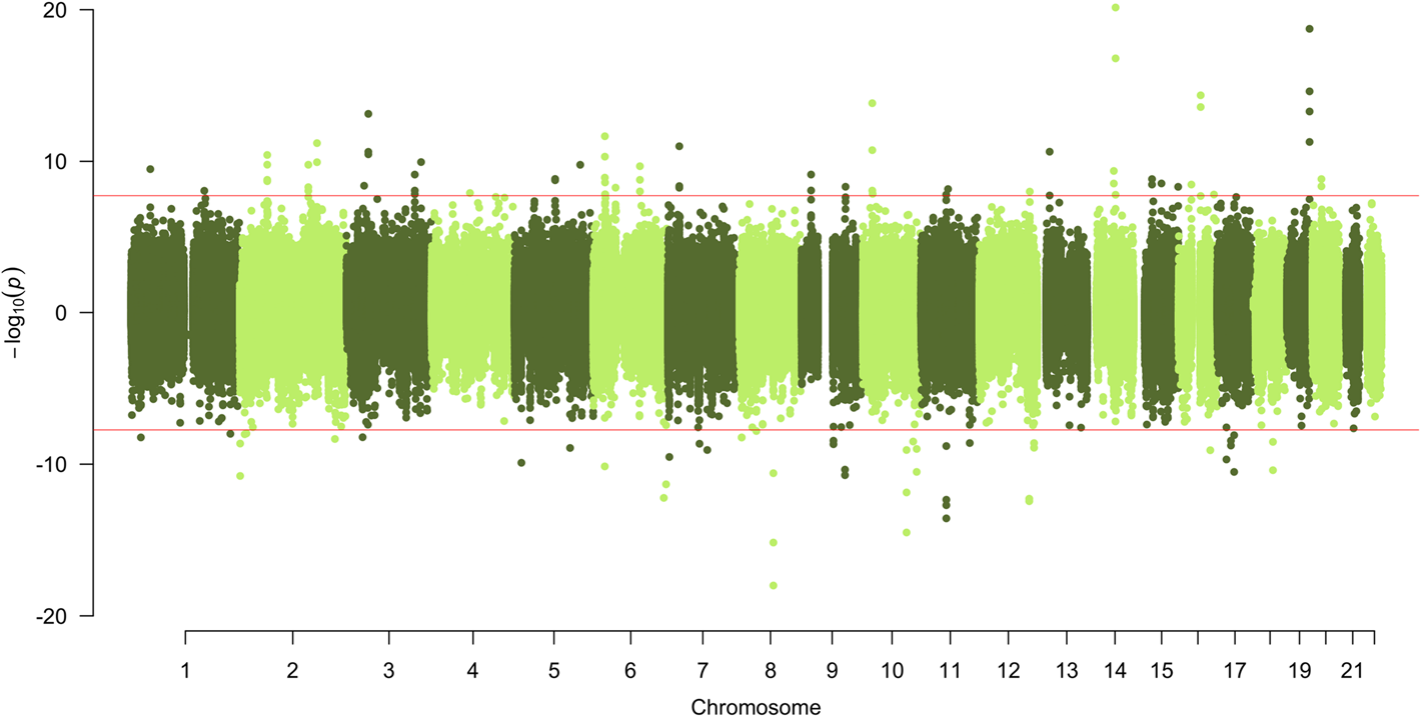
*Bidirectional Manhattan plot* of age-associated differentially methylated regions (a-DMRs). Loci with a positive correlated with age are displayed in the *positive y-axis* and those regions where DNA methylation changes negatively correlate with age are displayed in the *negative y-axis*

From our discovery sample set of 2238 MeDIP-seq DNA methylomes, we identified 115 individual 500-bp windows that passed a Bonferroni corrected significance level (*p* < 1.85 × 10^–8^, Fig. 1; bidirectional Manhattan plot) within these GWAS LD block regions. Due to overlapping and adjacent windows, these merged into 71 discrete age-associated differentially methylated regions (a-DMRs) (Additional file 1). The a-DMRs were 54.9 % and 45.1 % hypermethylated and hypomethylated with increasing age, respectively. They were on average the size of ~0.65 kb and include 1546 individual CpGs.

To ascertain the novelty of our results, we compared the 71 a-DMRs with results from 14 prior studies also performed in blood [3–16]. All these studies were array-based and therefore focused on a defined set of CpGs, except for McClay et al. (MBD-seq) [12] and Heyn et al. (WGBS) [6]. The array-based studies are less in sample size, except for a meta-analysis [9], with a maximum of around 1000 individuals, but the majority is much smaller. In total, 52 (73.2 %) of our a-DMRs were previously unidentified associations with age (Additional file 2: Table S1). Highlighting the use of the MeDIP-seq data, 29 of the a-DMR regions identified (40.8 %) were not covered at all by any CpG probe from either the 27 k or 450 k array platforms. A further 11 a-DMRs are covered on these arrays by only one or two CpGs.

### a-DMR chromatin segmentation is enriched for poised promoters and enhancers

**Fig. 2:**
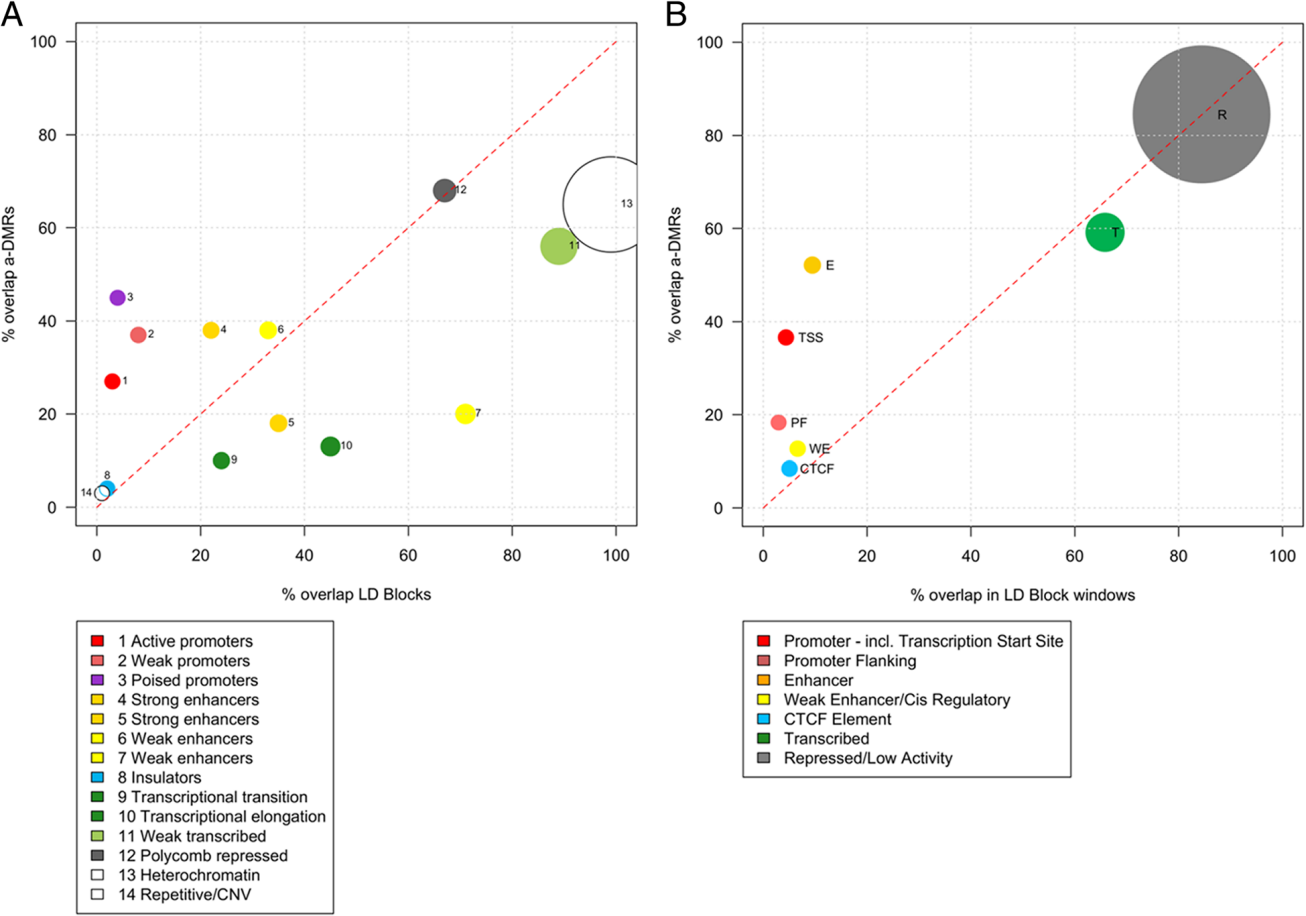
Chromatin segmentation enrichment. **a** Overlap with a-DMRs and GWAS LD blocks for ENCODE ChromHMM [30] genome segmentation from ENCODE in nine tissues (data via and graph adapted from Epiexplorer [85]). Strong poised promoter enrichment seen. Observed versus expected for the GWAS LD block regions. **b** Overlap with a-DMRs and LD blocks for combined genome segmentation from ENCODE (ChromHMM [30] and Segway [31]) in six tissues. Strong enrichment for enhancers is evident. Sphere size is proportional to genomic space. *TSS* predicted promoter region including transcription start site, *PF* predicted promoter flanking region, *E* predicted enhancer, *WE* predicted weak enhancer or open chromatin *cis* regulatory element, *CTCF* CTCF enriched element, *T* predicted transcribed region, *R* predicted repressed or low activity region

To identify chromatin-defined epigenomic functional enrichments, the a-DMRs were compared with both ChromHMM [30] and the Combined (ChromHMM and Segway [31]) genome segmentation analysis from nine and six tissue types, respectively (Fig. 2a and b). This was in a comparison with the GWAS LD blocks (using﻿ 500-bp non-overlapping windows), not the entire genome, as these regions already have an inherent functional increase [23]. Ageing changes have previously been identified in poised promoters [4] and in this ChromHMM analysis, this enrichment was particularly strong (Fig. 2a, ~45 % *cf*. ~3 % within LD blocks, χ^2^*p* < 2.2 × 10^−16^). Strong and weak promoters are also more prevalent, but interestingly, there is a separation between the delineated ChromHMM enhancer classes. Enhancer states 4 and 6 show increases, however 5 and 7 do not. The major constituent difference between these classes is higher levels of H3K4me2 (state 4: 99 % and state 6: 75 % versus state 5: 57 % and state 7: 3 %) and recent data from Wang et al. indicate that specifically high levels of H3K4me2 reliably identify transcription factor binding regions across different cells [32].

As stated we also compared the a-DMRs with the combined segmentation that incorporates overlap with the Segway algorithm and results in more discrete, reduced and potentially more accurate categories [31] (Fig. 2b). This classification does not include the poised promoter subcategory. It classifies an extremely strong enrichment in enhancer sequence across these multiple cell types (52.1 % a-DMRs versus 9.46 % in LD block non overlapping 500-bp windows, χ^2^*p* < 2.2 × 10^−16^).

### a-DMRs are enriched for genetic functional indicators

**Fig. 3:**
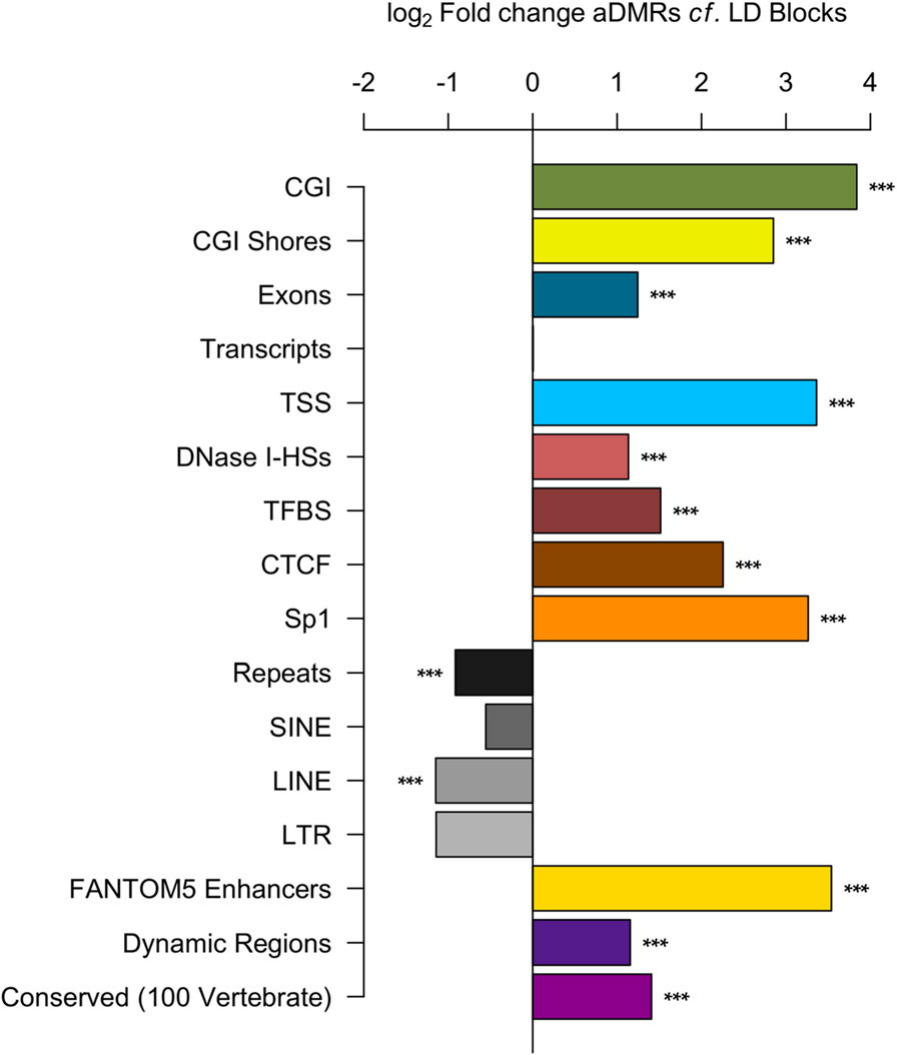
Fold enrichments for a-DMRs compared to LD block non-overlapped 500-bp windows. All categories are enriched in a-DMRs (χ^2^ *p* all < 1 × 10^–5^) except for transcripts (near identical fraction), SINE and LTR repeats (non-significant) and all repeats and LINE repeats, which are significantly depleted (χ^2^ *p* = 6.73 × 10^–9^ and 2.81 × 10^–3^, respectively). *CGI* CpG Islands, *TSS* transcription start sites, *DNase I HSs* DNase I hypersensitivity sites in 125 cell types, *TFBS* transcription factor binding sites, CTCF and Sp1 from all tissues (ENCODE v3), *Repeats* All, *SINE, LINE, LTR* repeats, FANTOM5 Enhancers [36], Dynamic Regions [66] and Conserved (100 Vertebrate) regions [87]

We then examined additional specified genetic and functional regions, in the same manner as the segmentation analysis above, by again comparison with the regions within the GWAS LD blocks, not the entire genome. This revealed greater fold enrichment for a-DMRs within CpG Islands (CGI) themselves (14.33-fold), even stronger, in fact, than for CpG Island shores (7.22-fold; Fig. 3). Thus a-DMRs differ from the findings in tissue-specific [33], reprogramming-specific [34] and species-specific [35] DMRs which are more prevalent in CGI shores. a-DMRs also showed enrichment within functional indicators such as DNase I hypersensitivity sites and transcription factor ChIP-seq binding sites, as well as even stronger fold enrichments for CTCF and Sp1 ENCODE ChIP-seq data across all tissues (χ^2^*p* all < 1 × 10^–10^). The eRNA expression validated FANTOM5 enhancer set [36] also showed a strong increase for a-DMR locations (χ^2^*p* = 2.2 × 10^–16^). Repeat classes in total were significantly depleted by comparison (χ^2^*p* = 6.70 × 10^–9^), although potential mapping issues to these regions may confound this.

### a-DMR Gene Ontology analysis

Gene Ontology enrichment was performed with the Genomic Regions Enrichment of Annotations Tool (GREAT) comparing the a-DMRs to the region contained within the LD blocks as the background set (Additional file 2: Table S2). This revealed an increase for categories (all Bonferroni *p* < 0.05), such as Molecular classifications of DNA binding; Nucleic acid binding transcription factor activity; Sequence-specific DNA binding transcription factor activity; and Nucleic acid binding. An enrichment in the PRD Gene family was also identified which includes multiple homeobox genes.

### Hypermethylated and hypomethylated a-DMR transcription factor binding site analysis

**Fig. 4:**
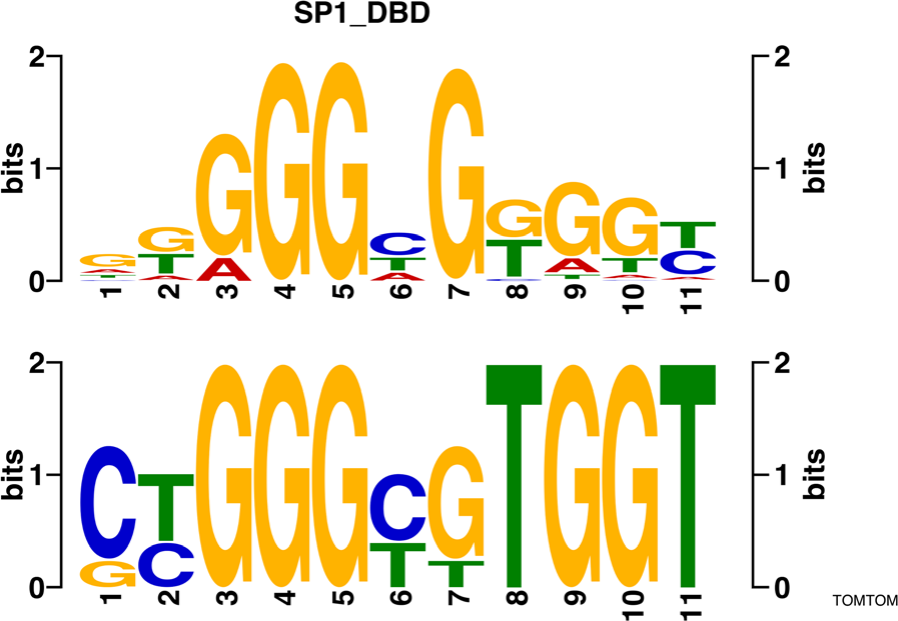
MEME-ChIP [38] enriched sequence identified in hypomethylated a-DMRs ﻿(below) and TOMTOM (v4.10.2) enrichment for the SP1 transcription factor motif (above)

We then examined the sequence within the 38 hypermethylated and 33 hypomethylated a-DMRs separately and explored for sequence enrichment of specific transcription factor binding sites (TFBSs) with both the transcription factor affinity prediction (TRAP) [37] and MEME-ChIP algorithms. [38] TRAP identified four TFBSs, three within hypomethylated DMRs including *NFE2L2* (*p* < 0.05, Benjamini–Hochberg corrected; Table 1), previously associated with age-related diseases [39]. The MEME analysis, which identifies sequences agnostically, before then comparing them to known motifs in TOMTOM, found three sequences in hypomethylated a-DMRs, that were found to closely match a number of motifs and only one match for hypermethylated a-DMRs (Table 2). These results included the SP1 motif, that is of interest as it corresponds with the ENCODE TF CHIP-seq enrichment data and also due to the known methylation-determining region effect of this TFBS [40] (Fig. 4). An enrichment for the binding motif of *KLF14* is noteworthy due this gene’s known role as a master *trans*-regulator in metabolism [41].

**Table 1:** TRAP transcription factor motif prediction

Rank	Combined *p* value	Corrected *p* value (Benjamini–Hochberg)	Matrix ID	Motif	a-DMR
1	3.84 × 10^–6^	4.53 × 10^–4^	MA0150.1	NFE2L2	Hypo
2	1.52 × 10^–5^	8.97 × 10^–4^	MA0041.1	FOXI1	Hypo
3	4.13 × 10^–5^	1.63 × 10^–3^	MA0041.1	FOXD3	Hypo
1	1.99 × 10^–5^	2.35 × 10^–3^	MA0152.1	NFATC2	Hyper

Enrichment for defined TFBS within hypomethylated and hypermethylated a-DMRs via the TRAP algorithm [37]

**Table 2:** MEME/TOMTOM agnostic motif analysis

Hypomethylated motif 1	SPDEF_DBD_3
Hypomethylated motif 2	FOXC1_DBD_1
	ONECUT3_DBD
	UP00037_1 (Zfp105_primary)
	UP00097_2 (Mtf1_secondary)
	ONECUT1_DBD
Hypomethylated motif 3	SP8_DBD
	SP1_DBD
	SP3_DBD
	KLF14_DBD
	Klf12_DBD
	KLF16_DBD
	SP4_full
	UP00011_2 (Irf6_secondary).
Hypermethylated motif 1	SPDEF_DBD_3

Enrichment for defined TFBS within hypomethylated and hypermethylated a-DMRs via MEME/TOMTOM motif analysis [38]

### No enrichment with blood-cell derived DNA methylome changes

The myeloid skew with age is an acknowledged confounding factor in the analysis of ageing changes. However, Yuan et al. have presented data that the majority of age-related drift is independent of increase of granulocytes in comparison to lymphocytes [42].

We had included available leukocyte fraction data in our linear mixed-effect model, but to further test whether we had accounted for these effects we performed additional investigations. We first compared our a-DMRs with the 500 leukocyte subtype-related differentially methylated CpG positions (L-DMPs) identified by Houseman et al. [43]. None of the a-DMRs overlap with these L-DMPs. Next, to check for blood-cell type bias we performed six epigenome-wide association studies (EWAS) in 54 monozygotic (MZ) discordant twins with precise white blood cell data for CD4^+^ helper T; CD8^+^ cytotoxic T; T cell; natural killer cell; CD34^+^ multipotential haematopoietic stem cell; and B cells [44] within the entire DNA methylome dataset. In this MZ analysis, 6.44 % of all windows have *p* < 0.05 for any of these six blood traits. However, a slightly lesser value of 6.38 % have a *p* < 0.05, and none near Bonferroni, within the a-DMR windows. Therefore, there is no strong evidence for enrichment for blood-cell changes in the a-DMRs (χ^2^*p* > 0.05).

### Validation of a-DMRs

In a dataset of 811 individuals that possessed 450 k array blood data (mean age, 58.0 years; age range, 18.6–81.7 years; 88.9 % overlap with MeDIP samples), we attempted to validate the fraction of a-DMRs that had overlapping probe(s). Thirty-eight a-DMRs possess at least one overlapping 450 k probe and, of these, 36 a-DMRs included probe(s) that passed quality control (QC). We performed a similar linear mixed effect model analysis for ageing methylation changes including the same covariant information as in our MEDIP-seq data. In this investigation, 32 (88.9 %) and 25 (69.4 %) of these 36 a-DMRs had at least one CpG with a nominal or Bonferroni significant (*p* < 1 × 10^–7^) results with the same directional change, respectively (Additional file 2: Table S3). This thus strongly supports our results but also shows the unique power of our analysis.

Of note, those a-DMRs that did possess an overlapping probe(s) were almost exclusively those where methylation increases with age, 34 of the total 38 and 31 of the 32 with at least nominally significant probes. This is thus consistent with early array studies that predominately found this direction of change, but also starkly revealing the bias of the CpGs present on the array.

### Replication of a-DMRs

The significant windows of the 71 a-DMR loci were assessed in a non-overlapping dataset of 2084 DNA methylomes also sourced from TwinsUK. This analysis was performed identically as for the discovery set, but with reduced covariate information for the genotype, smoking or leukocyte categories. Of the total number of 115 Bonferroni significant ageing-related windows, 96.5 % (111) show the same direction of effect, 84.3 % (97) nominal significance (*p* < 0.05) and 60.9 % (70) are even significant beyond the discovery Bonferroni level (*p* < 1.85 × 10^–8^). Of the merged 71 a-DMRs, 68 (95.7 %) have consistent windows with the same direction of effect, 57 (80.3 %) include a window that is nominally significant (*p* < 0.05) and in 38 (53.5 %), this reaches Bonferroni significance in this replication dataset (*p* < 1.85 × 10^–8^; Additional file 2: Table S1).

### Individual a-DMR loci

**Fig. 5:**
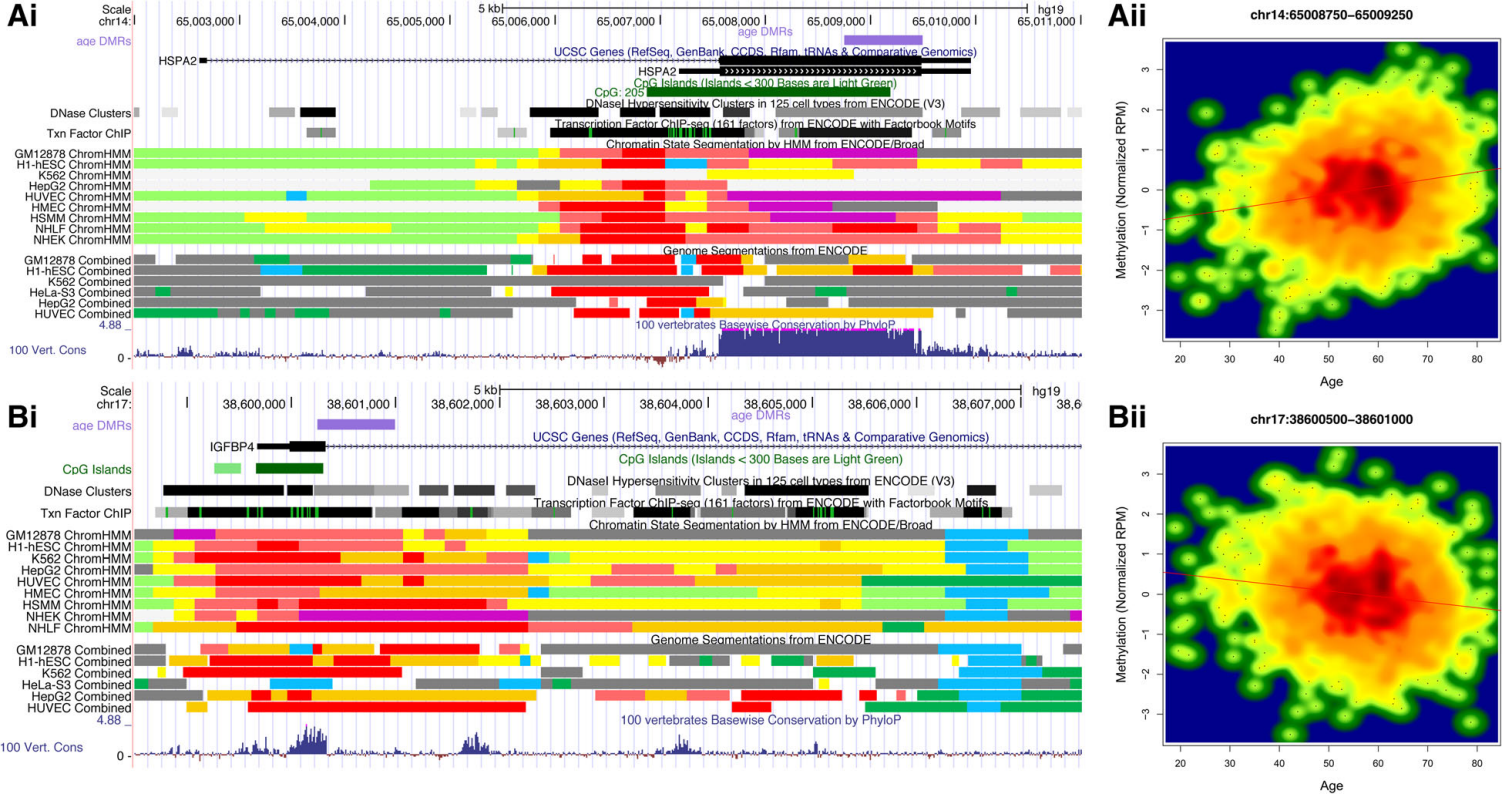
Selected a-DMRs within (i) genomic location; from top: a-DMRs (purple), gene, DNase I HS clusters, transcription factor ChIP-seq, ChromHMM segmentation, combined segmentation and conservation; and (ii) scatterplot: x-axis = age, y-axis = normalised methylation. **a**) *HPAS2* **b**) *IGFBP4*

We identified numerous a-DMRs in genetic regions with intriguing additional evidence from the literature for potential roles in age-related phenotypes. A selection of these a-DMR results are discussed below and are shown in Fig. 5, Additional file 3: Figure S1, and all in Additional file 4: Figure S2. The top a-DMR overlapped an intragenic 3′ CpG island and shore region within the *HSPA2* (Heat Shock 70 kDa Protein 2) gene (chr14:65,008,750-65,009,500), with the peak window *p* = 7.14 × 10^–21^. It is within a GM12878 ChromHMM predicted poised promoter and possesses strong vertebrate conservation (Fig. 5a). This precise locus was also previously identified in the MBD-seq study by McClay et al. [12] and also in a paediatric cohort via the 27 k array [5].

A number of physiologically interesting genetic loci are associated with the a-DMRs. This includes novel DNA hypomethylation in the *IGFBP4* promoter, with previously identified ageing-related increases in this gene product in both human serum and bone and a proposed role in bone loss in the elderly [45] (Fig. 5b). Another novel finding was within an intragenic a-DMR within *CDC14B*, residing over multiple-tissue enhancer predictions (Additional file 3: Figure S1c). A *Cdc14b* knockout mouse model displays an early ageing phenotype and a defective DNA damage response [46]. Murine data also shows enhancer evidence in this location and conserved epigenomic enhancer signals have been shown to be highly informative in disease models [47].

An intragenic a-DMR within *HFE*, the Haemachromatosis gene, resides upstream of the two common non-synonymous SNPs causative in this autosomal recessive condition (Additional file 3: Figure S1d). This is a low penetrance age-related phenotype with the symptoms only usually appearing between 30 and 50 years. The a-DMR overlaps predicted weak enhancer signal in hepatocellular-derived HepG2 ChromHMM data. If this novel change is occurring across additional tissues, such as the liver, it may be involved in the pathophysiology. However, there is no current array coverage with the nearest probe > ~1 kb further upstream away. Located in the gene body within the 0.3–8 kb downstream region of the promoter, it would also be defined as an undefined intragenic DMR (uiDMR), as per Schultz et al., shown to strongly influence expression [48].

Two a-DMRs reside within the vicinity of the *NR4A2* gene, one within an intragenic CpG Island shore and the other ~1.5 kb downstream from this gene, both overlapping predicted poised promoters (Additional file 3: Figure S1e). This gene itself is associated with nutritional status, postnatal development and hormonal imbalances [49]. Another a-DMR resides in the poised promoter of the growth hormone secretoagogue receptor (*GHSR*), or Ghrelin receptor, for the orexigenic hormone ghrelin that is active in the hypothalamus (Additional file 3: Figure S1f) with potential age-related effects [50]. Additional novel a-DMRs in genes of interest include *BMI1*, with an associated brain ageing phenotype in mouse knockout [51], and *C14orf39*, with a non-synonymous SNP associated with menarche [52].

Earlier studies have found numerous ageing changes within cancer-related genes and we also find many genes in this category, including previously identified loci in *CDKN2A* (also with a well-known role in ageing [53]), *MGA* and *ZNF577*, and novel changes in *ZNF300P1* (Additional file 3: Figure S1g), *STEAP1*, *FOXE1* and *PAX1*. Four overlapping significant windows comprise the 1.25 kb a-DMR in *ZNF577* that completely overlaps its 5′ promoter CpG island (Additional file 3: Figure S1h). This CpG island is known to be hypermethylated in lung and other cancers, but of particular interest to this blood tissue study, it was also found to be the only gene with promoter hypermethylation in a study of polycythaemia vera (PCV) *JAK2* V617F mutation individuals [54]. PCV is usually a late onset disease that is commonly diagnosed around 60–65 years. Subclinical PCV with undetectable low-level *JAK2* somatic clonal mutations could potentially contribute to this signature within this ageing cohort. This a-DMR location also overlaps with three previous studies [6, 8, 11].

All the above-mentioned genes were significant beyond the Bonferroni level in the replication set, except for *C14orf39* (replication *p* = 2.953 × 10^–6^).

### Multi-tissue regulatory enrichment

**Fig. 6:**
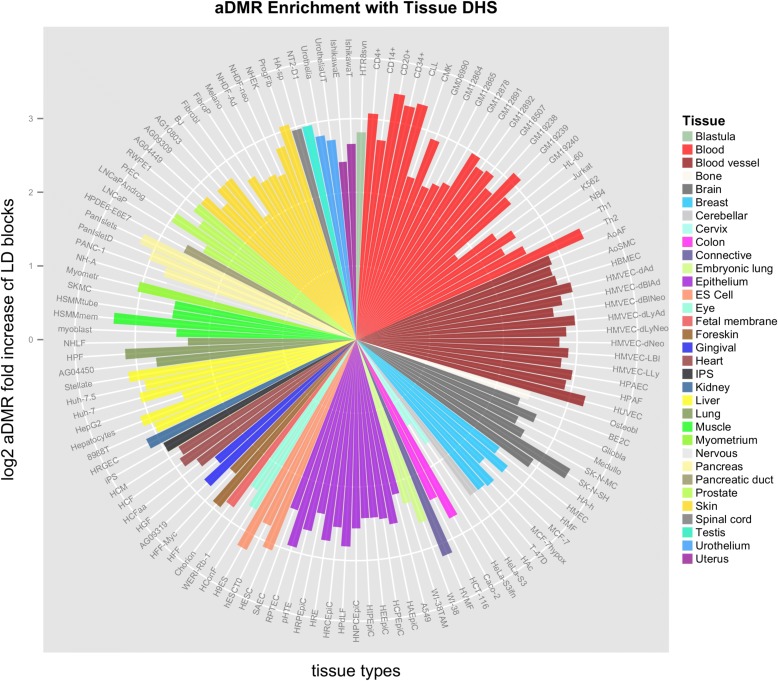
DHS fold enrichments for a-DMRs compared to the regions within LD blocks. *y-axis* indicates log_2_ fold enrichment. An enrichment across multiple tissue types is seen

We investigated the a-DMRs to ascertain whether they were more likely to fall within DHSs identified within blood-cell tissues and/or other cell types. This revealed that while the a-DMRs are enriched within this regulatory marker in blood cells, they were in fact significant across all tissue types and not particularly within haematological tissue (125 ENCODE DHS tissue types [55], all χ^2^*p* < 2.2 × 10^−16^; Fig. 6). This implies their systemic functional potential. Those that do reside within blood are an interesting subset due to their direct mechanistic interpretation. Twelve a-DMRs show a strong enrichment in blood-related DHS (≥50 % of the 22 blood-cell related analyses out of 125 total, Additional file 2: Table S4). These include novel a-DMRs within the promoter of *TAOK2* involved in the MAPK signalling pathway implicated in degenerative disease [56] (Additional file 3: Figure S1i) and within the promoter of an isoform of *FADS2* associated with liver omega desaturation [57]. a-DMRs that overlapped with previous studies include: an intragenic region within *AFF1*, the 3′ portion of the oncogenic fusion gene causative in acute lymphoblastic leukaemia [58] (Additional file 3: Figure S1j); the promoter CpG Island shore region of the transcription factor *KLF9* induced by oxidative stress [59]; and the *ZNF577* a-DMR mentioned above with respect to PCV.

### a-DMRs that reside within blood-related disease regions

Age-related DNA methylation changes within GWAS disease-associated regions where blood tissue may be directly involved in the pathophysiology make strong candidates for an epigenetic ageing and genetic interaction. The 71 a-DMRs reside within 64 GWAS LD blocks, containing 242 unique SNPs that have been related to 534 overlapping diseases and traits (Additional file 2: Table S5). When categorised by 15 broad disease classes [60], we found associations for  haematological measures (24 associations), autoimmune-related diseases (34) and large numbers of associations across an overlapping range of serum metabolites (257). All of these may influence or could be represented in blood physiological changes (Additional file 2: Table S6). In fact, a large proportion of the a-DMRs (33, ~46.48 %) are implicated in these three broad disease categories. Additionally, within the cancer category, there are two blood-related malignancies with a-DMRs within their GWAS association risk loci: multiple myeloma and acute lymphoblastic leukaemia.

### Age-related disease enrichment

We additionally parsed our a-DMR results for an enrichment for ageing-related diseases. While many disorders and phenotypes have some complex age component, we restricted this analysis to adult-onset cancers, age-related and degenerative neurological, musculoskeletal, metabolic, cardiac and cerebrovascular diseases, as well as cognitive decline and age-of-onset analyses (Additional file 2: Table S7). This revealed an enrichment for the a-DMRs to reside within GWAS LD blocks for an age-related phenotype. These disorders are found in 26.7 % of the GWAS LD blocks, compared to 39.4 % of the a-DMR co-locating GWAS LD blocks, Fisher’s exact test *p* = 0.02, OR = 1.81, CI 1.08–3.02). Permutation analysis, by randomly selecting 1000 times the age-related fraction of GWAS LD blocks from the total set and then testing overlap with the observed a-DMRs, significantly supported this enrichment (empirical *p* value = 0.021).

### Interaction with genotype

While genetic effects can strongly confound an EWAS analysis, we attempted to explore our dataset for any evidence of a genetic facilitated effect, through common haplotype differences, on DNA methylation changes occurring with age. We performed an interactive analysis between the LD block haplotype tagging genotype and age with DNA methylation. We then overlapped these results with the a-DMR regions, where we had excluded genotype being the sole driver of the signal. Thus we are not looking for obligatory differences, but instead an accentuated facilitated ageing signature on either the risk or non-risk haplotype, with the potential to then have influence on the associated phenotype or disease.

Three a-DMRs were identified that co-located with significant interaction results (all *p* < 0.05, Additional file 5: Figure S3A–C). One of these included the enhancer located a-DMR in the *TRAK1* locus associated with the late onset neoplasm, multiple myeloma (rs1052501). Other SNPs within this LD block associate with additional phenotypes, such as blood pressure. The G risk allele carriers did not show the same increase in DNA methylation with age, possibly indicating that these individuals have a prematurely higher DNA methylation in this locus and therefore the age-related trend is not seen. The a-DMR within the promoter of the *MGA* promoter also shows this trend, with the SNP rs28374715 in this LD block associated with ulcerative colitis. The a-DMR within the promoter of the *IGFBP4* gene, previously mentioned with regards to age-related bone loss, shows methylation change in those individuals with the SNP rs584438 T allele, which is related to height, decreasing more strongly than the non-associated haplotype.

## Discussion

Epigenomic changes with advancing years provide a novel avenue to explore the physiology and mechanisms of ageing [9]. This is clear with the observed discordance between chronological and predicted epigenetic age and its association with mortality [18]. The driving role of heterochromatic epigenetic changes in ageing has also been displayed in the human premature ageing disorder Werner syndrome [61]. In this study, we performed the largest analysis of DNA methylation changes with age to date and identified novel age-related regions that show strong functional enrichment over a range of publicly available evidence. In comparison to previous array findings, in almost half of these loci DNA methylation decreased with age. Those a-DMRs that did overlap array probes were almost exclusively regions that hypermethylate with age, clearly displaying the bias of the array to CpGs possessing this directional change only. The identification of these novel ageing changes in loci with no array coverage reinforces the insights more genome-wide methodology can provide. Furthermore, additional evidence that strong associations are identified in this study comes from the convincing replication in an independent large dataset.

The a-DMR loci co-locate with many physiologically interesting genes, including novel and robustly replicated findings in *IGFBP4*, *CDC14B*, *HFE*, *BMI1* and *TAOK2*, among others. These loci reside within regions that have strong genetic associations with common diseases and therefore are novel candidates for potential genetic and epigenetic interactions, particularly for late-onset conditions. Blood-derived DNA methylation ageing changes can be concordant across a range of other tissues [9, 15], consequently some of these alterations may act in the most disease-relevant tissue. We identified that our ageing changes were enriched for the regulatory regions of multiple tissue types. Furthermore, epigenetic variation is strongly intertwined with sequence-specific transcription factor interaction and binding [62, 63] and thus these changes can be a key to unlocking the precise molecular mechanisms involved.

Multiple cell-types are present in peripheral blood, therefore its analysis represents a meta-epigenome [64], so that changes in subpopulation proportions will be detected as epigenetic variation. However, we found no strong evidence of enrichment for blood cell-type related DNA methylation changes within these a-DMRs. Additionally, contribution to DNA methylome variation by subclinical or unknown pathology, as identified in recent age-related brain analysis via neuropathologies [65], cannot be completely excluded. Although this is unlikely to be present in our samples, since TwinsUK participants are recruited as predominately healthy volunteers, unselected for any diseases.

There are inherent power benefits from this study that is six-fold larger in sample size to the only comparable previous analysis for regional changes by McClay et al. [12]. Robust DMRs are strongly enriched for function [66]; however, denser coverage is required for successful DMR calling than is provided by 450 k array [67]. Fundamentally, arrays are designed to identify individual CpGs in contrast to regional approaches, such as MeDIP-seq and MBD-seq, which can only identify consistent regional variation. Thus while the 71 a-DMRs may not initially compare to multiple a-DMPs identified with arrays, this is inherent with these methodical differences. Our results are directly analogous to the 11 a-DMRs identified in the other large-scale genome-wide approach of McClay et al. [12].

The finding in this study of aberrant DNA methylation within disease-related loci proposes potential interrelationships with ageing changes and disease susceptibility or downstream consequence. Evidence that age-associated DNA methylation may predispose to cancer by reducing threshold of malignant transformation has been previously shown [68] and is implicated in the increased cancer incidence with age [15]. The ability of epimutations to pathogenically drive disease in the same fashion as genetic mutation, such as in acute myeloid leukaemia [69], further support the pathological potential of these changes and also the possibilities in regards to non-malignant disease [70].

## Conclusion

The loci we have investigated are associated with human traits and disease through robust and replicated GWAS. Thus these epigenetic changes with age will be valuable measures to incorporate in these disease models. With the availability of genotype data, we have also been able to test and identify variation in this signal between risk and non-risk haplotypes. With a more detailed understanding of the haplotypic nature of both genetic risk [71, 72], but also epigenetic risk, through the integration of obligatory and facilitated epigenetic changes [27, 73, 74], a more precise understanding of common disease will emerge. This well-defined allele-specific genetic and epigenetic variability should accelerate mechanistic discoveries into ageing’s role in late-onset disorders and the biology of human ageing and disease.

## Methods

### Participants

Participants are from the deeply phenotyped UK Adult Twin Register (TwinsUK Resource) [75] based at St Thomas’ Hospital, London. Phenotyping occurs at interview when blood is also taken for haematological analysis and DNA extraction. Storage is in EDTA tubes at –80 °C. Nucleon Genomic DNA Extraction Kits are used for DNA extraction which are then stored at –20 °C in TE buffer. Haematological analysis for full blood count was performed on the majority of extracted bloods. Smoking status is recorded at this time or within the nearest five years via questionnaire if not available. Zygosity is determined by twinning questionnaire and confirmed by genotyping.

The discovery set consisted of 2238 DNA methylomes, which were all female, therefore sex-specific modifications were removed [76], and included longitudinal data with two or more time points on 408 individuals (mean time difference 2.18 years) and single time point data on 1350. These 1758 individuals included 203 MZ twin pairs and 489 MZ singletons and 371 dizygotic (DZ) pairs and 121 DZ singletons, therefore comprising equal numbers of MZ (50.9 %) and DZ (49.1 %) individuals from a total of 1184 unique families. The age at collection date of blood for DNA extraction was in the range of 19–82.2 years (mean age, 55.99 years; median age, 56.60 years; std. dev. 10.32 years).

### MeDIP-seq

DNA sample preparation, MeDIP reaction and Illumina second-generation sequencing were all performed at BGI-Shenzhen, Shenzhen, China. Fragmentation of the whole peripheral blood TwinsUK DNA was via sonication with a Covaris system (Woburn, MA, USA). Libraries for sequencing were prepared from 5 ug of fragmented genomic DNA. End repair, <A > base addition and adaptor ligation steps were performed using Illumina’s DNA Sample Prep kit for single-end sequencing. The anti-5mC antibody (Diagenode) was used to immunoprecipitate the adaptor-ligated DNA and the resultant MeDIP was validated by quantitative polymerase chain reaction (PCR). This captured DNA was then purified with Zymo DNA Clean & Concentrator™-5 (Zymo Research) and subsequently amplified with adaptor-mediated PCR. Fragments of size 200–500 bp were selected by gel excision and then QC assessed by Agilent BioAnalyzer analysis. These libraries were then sequenced on the Illumina platform. Sequencing data passed initial QC for base composition assessed via FASTQC (v0.10.0) (http://www.bioinformatics.bbsrc.ac.uk/projects/fastqc). MeDIP-seq data were processed with BWA (Burrows-Wheeler Aligner) alignment [77] (passing a mapping quality score of Q10), with duplicates removal, FastQC and SAMTools [78] QC and MEDIPS(v1.0) [79] for MeDIP-specific analysis, QC, reads per million (RPM) and absolute methylation score (AMS) generation. The average high quality BWA aligned reads was ~16.9 million per sample for the discovery set of 2238 and ~16.8 million for the replication set of 2084. Further QC was performed via R (correlation matrix, hierarchical clustering, dendogram, heatmap, density plot) and batch effects inspection by principle component analysis. Processed data for statistical analysis are BED files of genomic windows (500-bp, 250-bp slide) with RPM scores. All human genome coordinates, calculations performed and those cited are in build hg19/GRCh37.

### GWAS LD blocks

The analysis was performed on the a priori functionally enriched genomic regions contained within the LD blocks of the NIH GWAS SNP catalogue [24, 25]. The LD blocks were ascertained from the GRCh37 genetic map, downloaded from Center of Statistic Genetics, University of Michigan, Locuszoom 1.3 [80], with recombination rate of 10 cM/Mb boundaries. LD blocks were further pruned to those ≤ 10 Mb in size. We selected the 8093 curated GWAS SNPs with *p* value < 1 × 10^–7^ deposited within the NIH GWAS catalogue as at December 2014. Due to co-associations for the same SNP, these are 5522 unique individual SNPs and 5477 of these resided within the above-identified LD blocks. In fact, these represented 2709 distinct LD blocks once accounting for SNPs present within the same block. These regions cover ~22.1 % of the human genome.

### Age-associated DNA methylation analysis

All statistical analyses were run in the R (3.0.0) environment [81]. The lme4 package [82] was employed to perform a linear mixed effect analysis of the relationship between chronological age at DNA extraction and DNA methylation, which was represented as normalised RPM values within the 500-bp windows. Additional fixed effects terms included allelic count of the haplotype-tagging SNP, smoking status, batch, blood cell subtypes (lymphocytes, monocyte, neutrophil and eosinophil) with family and zygosity as random effects. This model for DNA methylation age analysis is similar to that used previously in array based analyses [15] with the additional inclusion of genetic allelic information. *p* values were calculated with the ANOVA function by likelihood ratio test of the full model including age versus null model excluding this variable. A Bonferroni multiple testing correction was calculated by the total number of DNA methylation windows included in the analysis (2,708,462), giving a *p* value significance level of <1.85 × 10^–8^ (see “Study Design” in Additional file 6: Figure S4).

The immunoprecipitation reaction in MeDIP-seq data is extremely susceptible to the influence of genetic variation in CpG number (due to CpG-SNPs, CNVs, indels and STRs), leading to a direct relationship between the number of methylated cytosines in the DNA fragment and the amount of DNA captured by the antibody as discussed by Okitsu and Hsieh [22]. We accounted for this influence by the inclusion of the haplotype-tagging common SNP data for each LD block examined within our statistical model. We further also removed the ENCODE poor mappability blacklist regions [28] from any further analysis (13,726 500-bp windows). Shared *trans* factors, however, cannot be accounted for, although are much less frequent [83], but the large replication set, described below, adds powerful support to the discovery findings.

An interaction between genotype and age was directly tested for by comparing the full model, but with DNA methylation and age included as interacting factors, and the full model in the initial analysis, with again a likelihood ratio test via ANOVA to derive significance levels. As the direct confounding of common genetic effects was included in the initial a-DMR analysis with strict Bonferroni cutoff, we then overlapped these results with our a-DMR set to identify those robust a-DMRs with potential evidence of interaction.

### Novelty of a-DMRs analysis

We identified 14 previous studies [3–16] that had been performed for DNA methylation changes in blood with respect to age with available data for comparison and downloaded these results placing CG positions at their correct co-ordinates from Illumina array annotation files and converting all that were in previous builds to hg19/GRCh37 via UCSC tools liftOver [84]. These were merged and compared via BEDtools (v.2.17.0) and are available in Additional file 7.

### Blood-cell discordant monozygotic twin EWAS

A MZ discordant EWAS in 54 MZ pairs that possessed precise white blood cell data within this DNA methylome dataset was performed. These data were generated by Roederer et al. [44] and included calculations for CD4^+^ helper T, CD8^+^ cytotoxic T, T cell, natural killer cell, CD34^+^ multipotential haematopoietic stem cell and B cells. MZ twin pairs’ discordance for each blood-cell trait was calculated. The 500-bp DNA methylome windows for analysis required ≥90 % of individuals with non-zero values. Residuals from the linear regression model of RPM methylation scores with adjustments for smoking, leukocyte counts, age at DNA extraction and batch were normalised (qqnorm) and then the high–low difference significance was compared by one-sided T-test.

### Enrichment analysis

Initial exploration of a-DMRs was performed via Epiexplorer [85]. This enabled enrichment for chromatin state (ChromHMM), histone modifications and additional ENCODE and Roadmap data to be investigated first. Comparisons were made with ChromHMM in nine tissues from Encode Broad HMM (Gm12878; H1hesc; Hepg2; Hmec; Hsmm; Huvec; K562; Nhek; Nhlf) and then with combined segmentation in six tissues - Encode AwgSegmentation (Gm12878; H1hesc; Helas3; Hepg2; Huvec; K562) via UCSC. Overlap in genetic and functional data was calculated with BEDtools (v.2.17.0) command intersectBed, compared with non-overlapping LD block 500-bp windows with –f 0.1 parameter (moderate overlap). The genetic regions compared for enrichment were CpG islands, TFBSs from ENCODE v3 (690 datasets from wgEncodeRegTfbsClusteredV3 [86]), DHS in 125 cell types from ENCODE analysis [55] and Vertebrate Multiz Alignment and Conservation (100 Species) from 100Vert_El_phastConsElement100way bedfile (~10.1 m regions), all downloaded from UCSC [87]. FANTOM5 enhancers regions were from Anderson et al. [36] and ‘Dynamic’ regions from Ziller et al. [66].

A further a-DMR enrichment analysis was performed with the Genomic Regions Enrichment of Annotations Tool (GREAT v3.0.0) [88] region-based binomial analysis with basal, but the extension parameters reduced from the default (constitutive 5.0 kb upstream, 1.0 kb downstream and up to 100 kb max extension, not 1 Mb). Curated regulatory domains were included and all LD block regions were used as the background set.

For TFBS motif enrichment, we used the TRAP method [37] and the MEME suit (MEME-ChIP [38] and TOMTOM (v4.10.2) [89]). FASTA sequence files of the 71 a-DMRs were inputted as separated hypomethylated and hypermethylated groups. In TRAP they were compared to the JASPAR vertebrates with a background model of human promoters. MEME-Chip compared with a set of 1229 DNA motifs, in the range of 7–23 in length (average length 13.8), from the database Human and Mouse (in silico).

### Validation analysis

Within the a-DMRs, 116 CpG probes from the Infinium Human Methylation450 BeadChip reside that passed QC, as detailed below. These were blood-derived CpG methylation scores from 811 female individuals, 89.1 % overlapped with the MeDIP samples. QC included removal of probes that failed detection in at least one sample and with a bead count less than 3 in more than 5 % of the samples, and probes for which the 50 bp sequence aligned to multiple locations in the genome. Cell type proportions were estimated for CD8+ T cells, CD4+ T cells, B cells, natural killer cells, granulocytes and monocytes [43]. All data were normalised using the intra-array normalisation, beta-mixture quantile dilation (BMIQ) [90] to correct for probe type bias. The validation was performed using a linear mixed effects model fitted on standardised beta values per probe (N(0,1)) with age, genotype as allelic count, smoking status, beadchip, position on the beadchip, granulocytes, monocytes and CD8+ T cells as fixed effects, as well as family and zygosity as random effects. To assess for significance, ANOVA was used to compare this model to a null model without age.

### Replication analysis

We utilised an additional 2084 peripheral blood MeDIP-seq data, also available from TwinsUK, for our replication set. None of these individuals were present in the discovery set and do not differ from that set in any selective way. These samples were in the age range of 16–82.2 years (mean age, 51.00 years; median age, 53.40 years; std. dev. 14.91), were 87.04 % female and included 1897 samples from 1710 MZ individuals (582 pairs, 546 lone) and 187 samples from 159 DZ individuals (46 pairs, 67 lone), with 215 possessing data from >1 time point. Analysis was performed as for the discovery set using an identical linear mixed effect model, for normalised DNA methylation (500 bp windows) with age at DNA collection; however, these samples did not possess genotype, smoking or leukocyte information, and therefore only included the additional fixed effect of batch and random effects of zygosity and family.

### Tissue-specific investigation

The DHS from 125 cell type experiments from ENCODE analysis [55] were used for tissue-specific analysis of the a-DMRs. This dataset includes 22 blood tissue related samples. Broad disease classes were taken from Maurano et al. [60].

## Additional files

Additional file 1: BED file of a-DMRs with UCSC browser header. (BED 1 kb) Additional file 2: Supplementary **Tables S1–7.** (XLSX 1125 kb) Additional file 3:
**Figure S1.** Selected a-DMRs within (i) Genomic Location; From Top: a-DMRs (purple), Gene, DNase I HS Clusters, Transcription Factor ChIPseq, ChromHMM Segmentation, Combined Segmentation, & Conservation. (ii) Scatterplot: X axis = Age, Y axis = Normalised Methylation. C) CDC14B, D) HFE, E) NR4A2, F) GHSR, G) ZNF300P1, H) ZNF577, I) TAOK2, J) AFF1 locus. (PDF 5.8 mb) Additional file 4:
**Figure S2.** All a-DMRs within (1) genomic location. *Top*: a-DMRs (*purple*), gene, DHS clusters, transcription factor ChIP-seq, ChromHMM segmentation, combined segmentation and conservation; and (2) *scatterplot*: *x-axis* = Age, *y-axis* = Normalised methylation. (PDF 34909 kb) Additional file 5:
**Figure S3.** Genotype-interaction analysis. Variation in ageing signal depending on genotype. Homozygote SNP trait related allele is in *red. Left*: *Scatterplot*: *x-axis* = Age, *y-axis* = Normalised methylation. *Right*: *Boxplot*: three genotype categories separated into two groups by ≤ or > median age (56.6 years). (PDF 910 kb) Additional file 6:
**Figure S4.** Study design *flowchart*. (PDF 152 kb) Additional file 7: BED file of combined 14 ageing studies. (BED 2508 kb)

## Abbreviations

a-DMR: Ageing-related differentially methylated region
CGI: CpG island
DMP: Differentially methylated position
DMR: Differentially methylated region
EWAS: Epigenome-wide association study
GWAS: Genome-wide association study
LD: Linkage disequilibrium
MeDIP-seq: Methylated DNA immunoprecipitation second-generation sequencing
WGBS: Whole genome shotgun bisulfite second-generation sequencing
