# Supplementary figures and images for: Novel regional age-associated DNA methylation changes within human common disease-associated loci
Source: Genome Biol. 2016 Sep 23;17:193. doi: 10.1186/s13059-016-1051-8 (PMC5034469; doi:10.1186/s13059-016-1051-8)

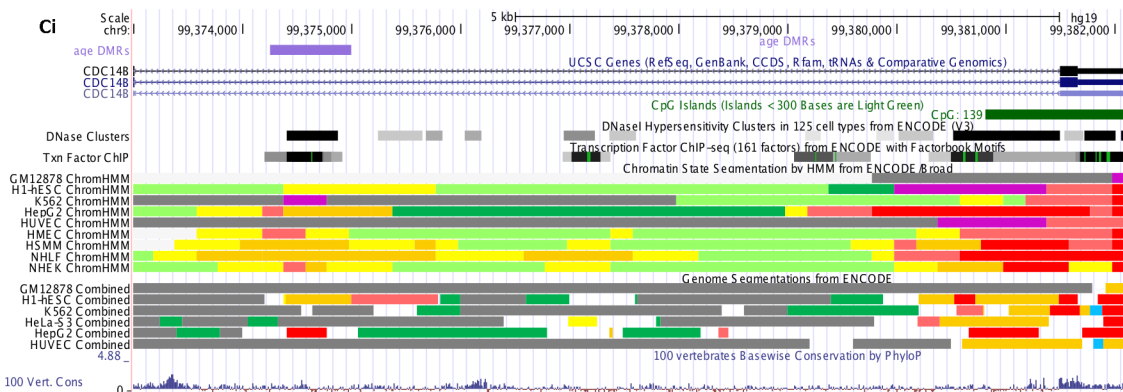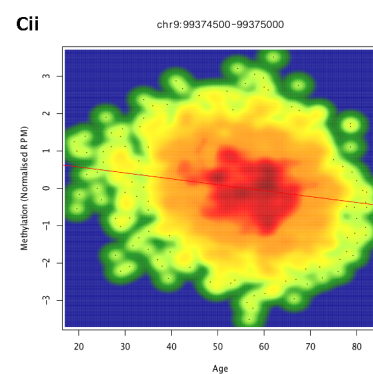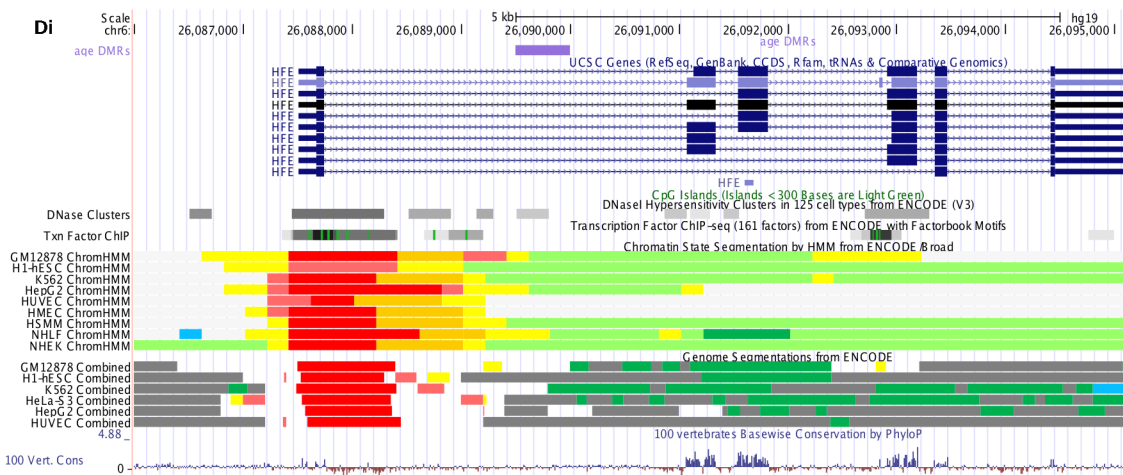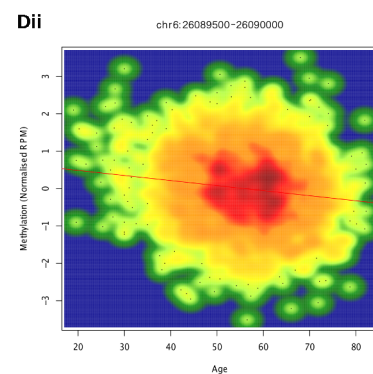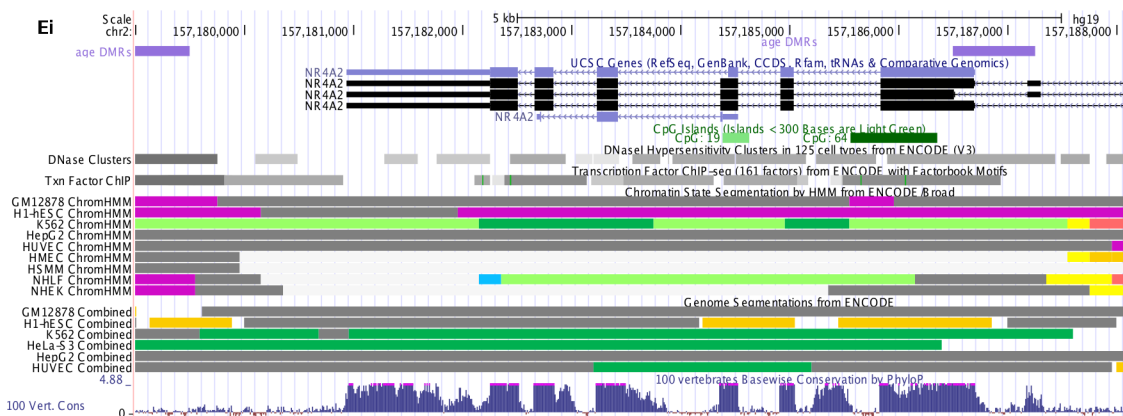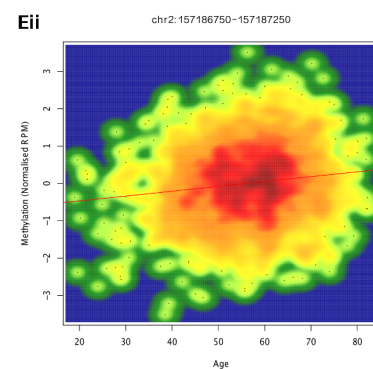

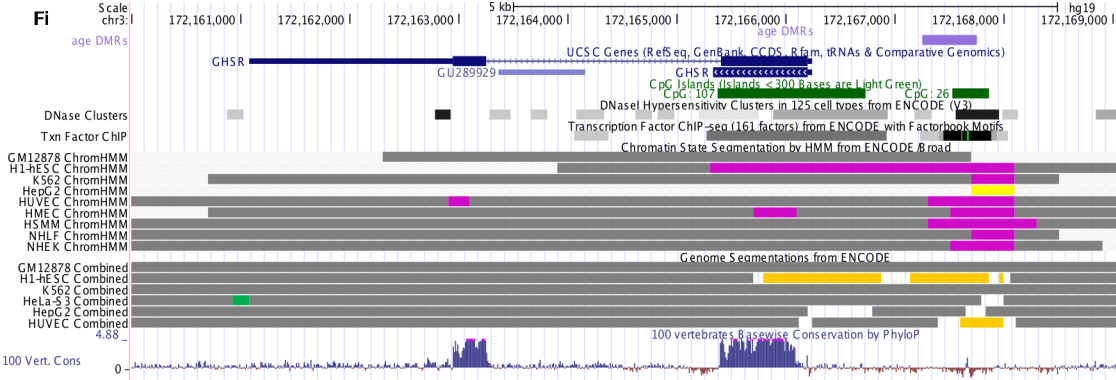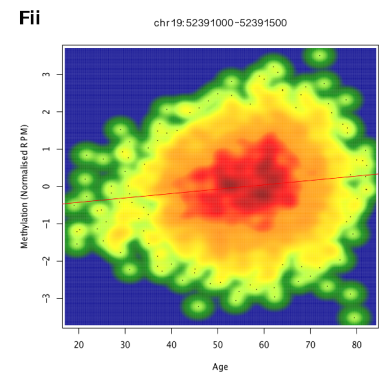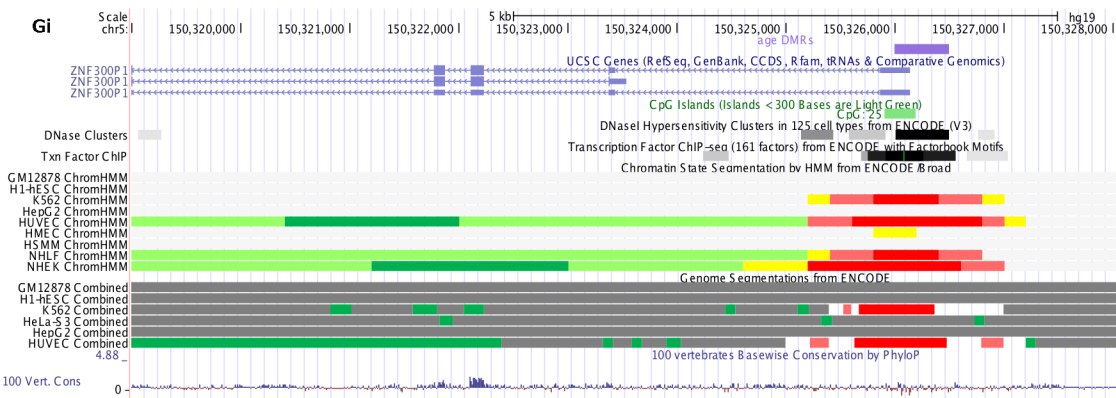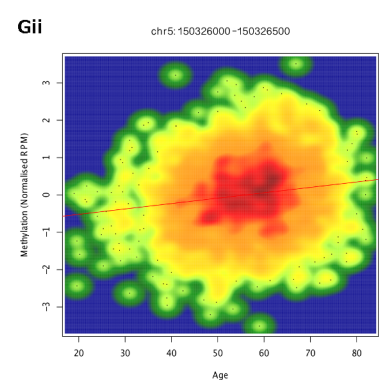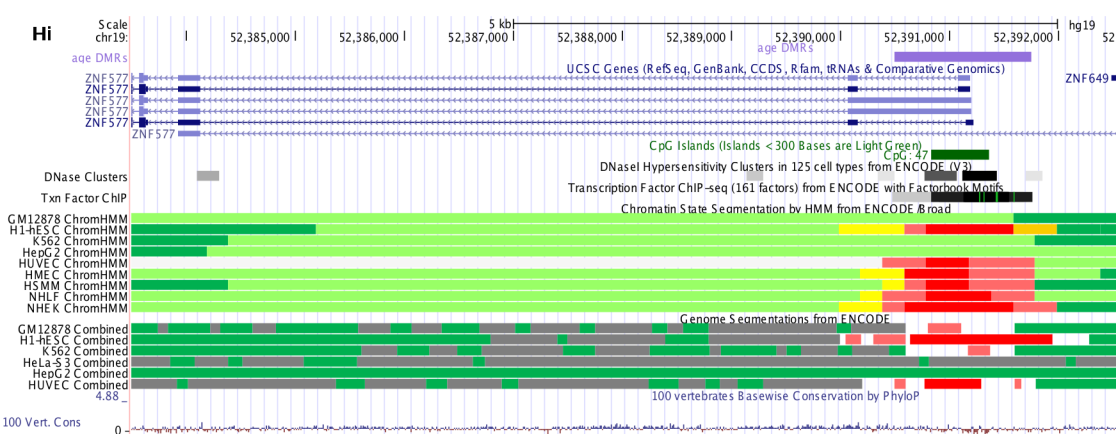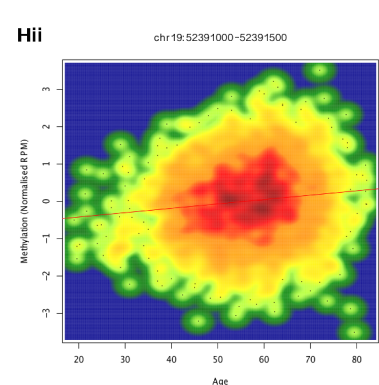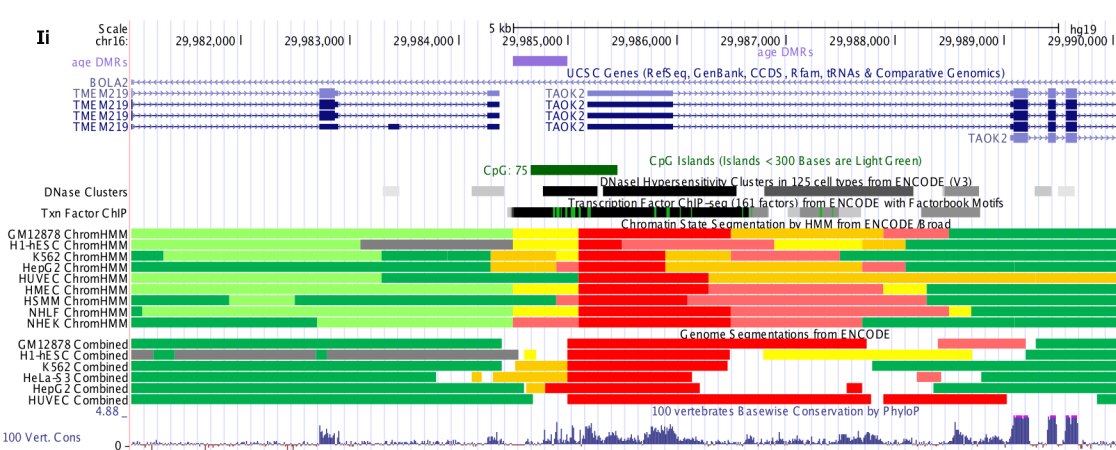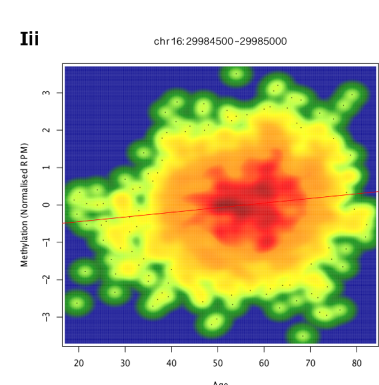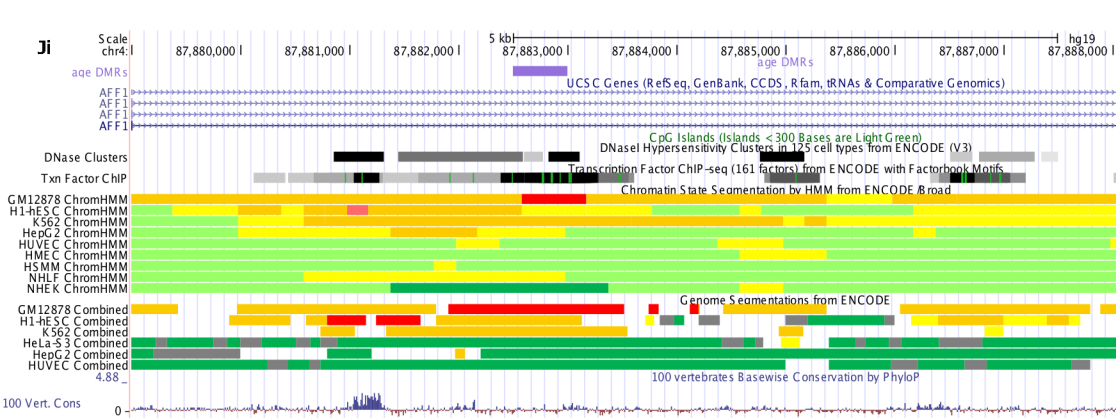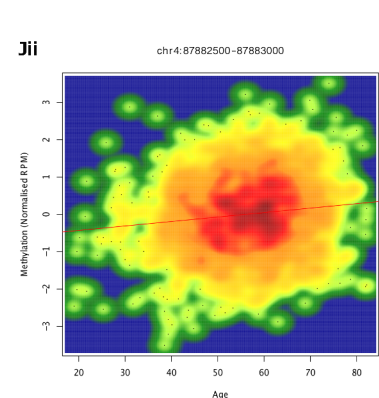

Supplement: Additional file 3: — Figure S1. Selected a-DMRs within (i) Genomic Location; From Top: a-DMRs (purple), Gene, DNase I HS Clusters, Transcription Factor ChIPseq, ChromHMM Segmentation, Combined Segmentation, & Conservation. (ii) Scatterplot: X axis = Age, Y axis = Normalised Methylation. C) CDC14B, D) HFE, E) NR4A2, F) GHSR, G) ZNF300P1, H) ZNF577, I) TAOK2, J) AFF1 locus. (PDF 5.8 mb) [file 13059_2016_1051_MOESM3_ESM.pdf]

**chr3:42113500-42114000**

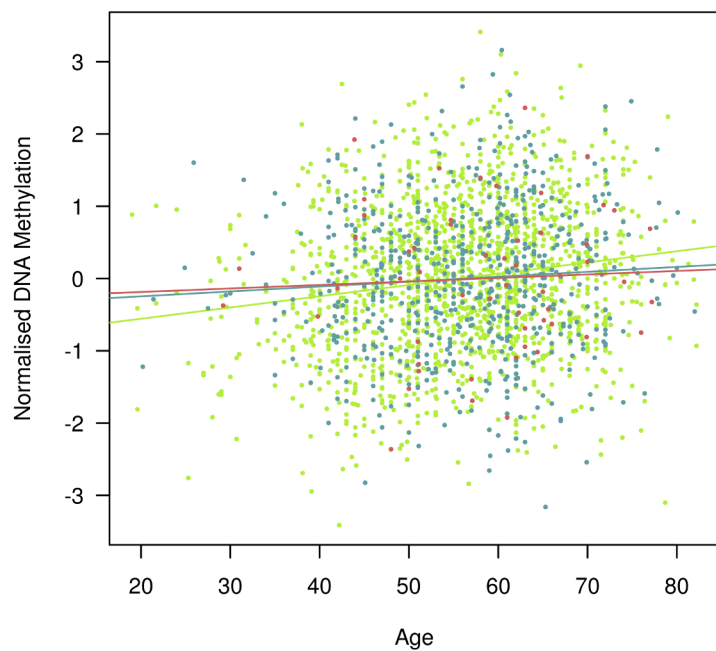

**rs1052501**

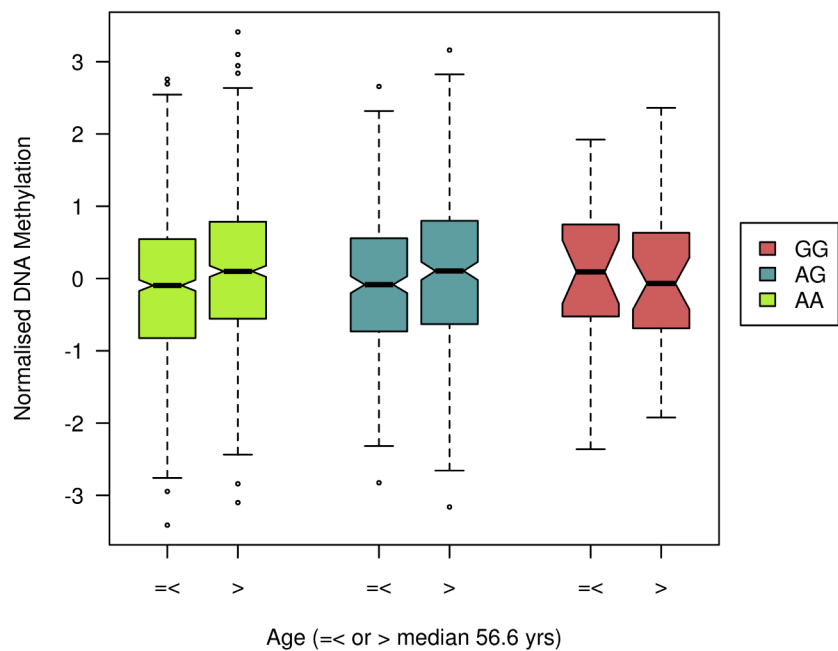

**chr15:41951750-41952250**

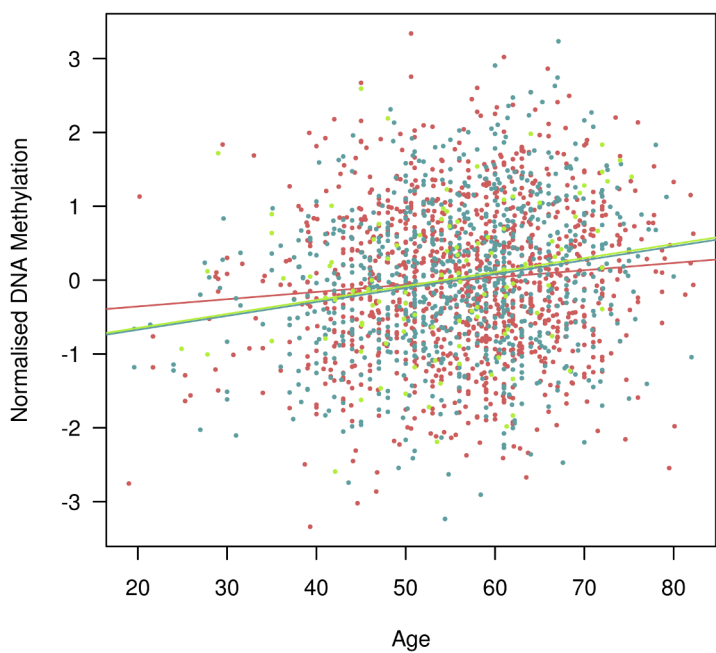

**rs28374715**

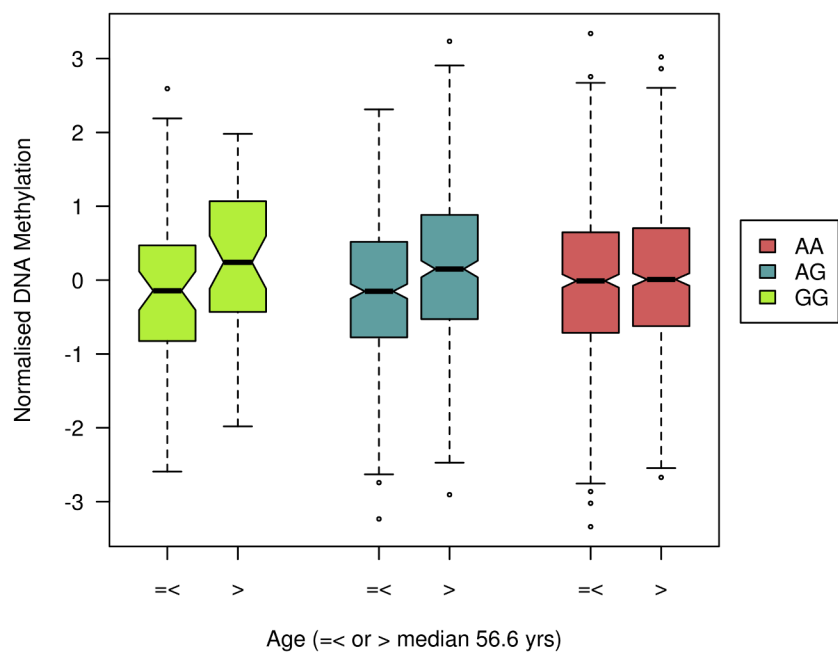

**chr17:38600500-38601000**

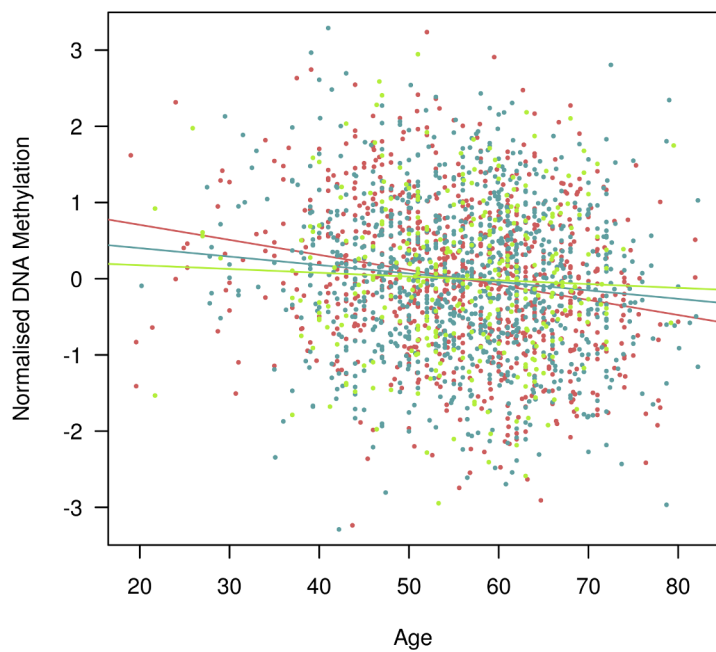

**rs584438 chr17:38600500-38601000**

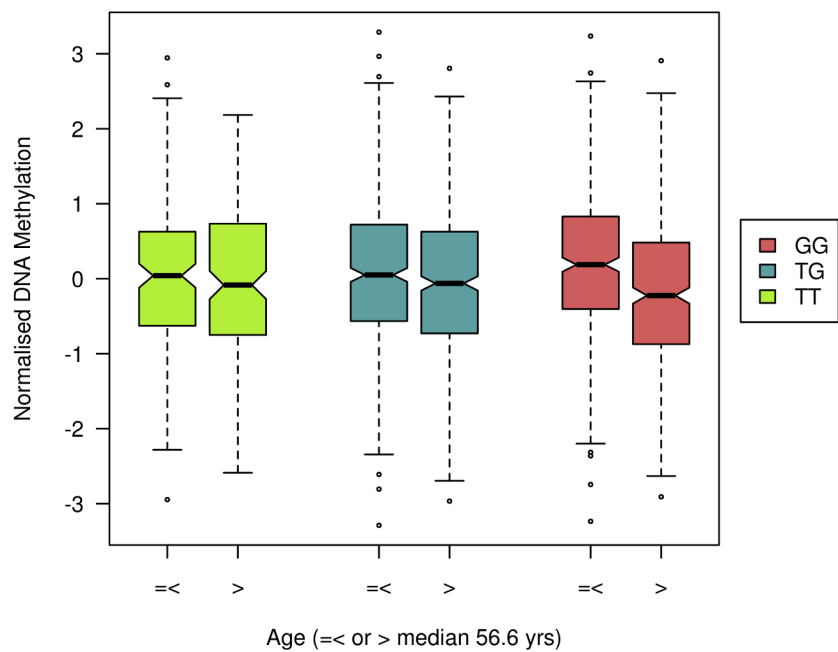

Supplement: Additional file 5: — Figure S3. Genotype-interaction analysis. Variation in ageing signal depending on genotype. Homozygote SNP trait related allele is in red. Left: Scatterplot: x-axis = Age, y-axis = Normalised methylation. Right: Boxplot: three genotype categories separated into two groups by ≤ or > median age (56.6 years). (PDF 910 kb) [file 13059_2016_1051_MOESM5_ESM.pdf]

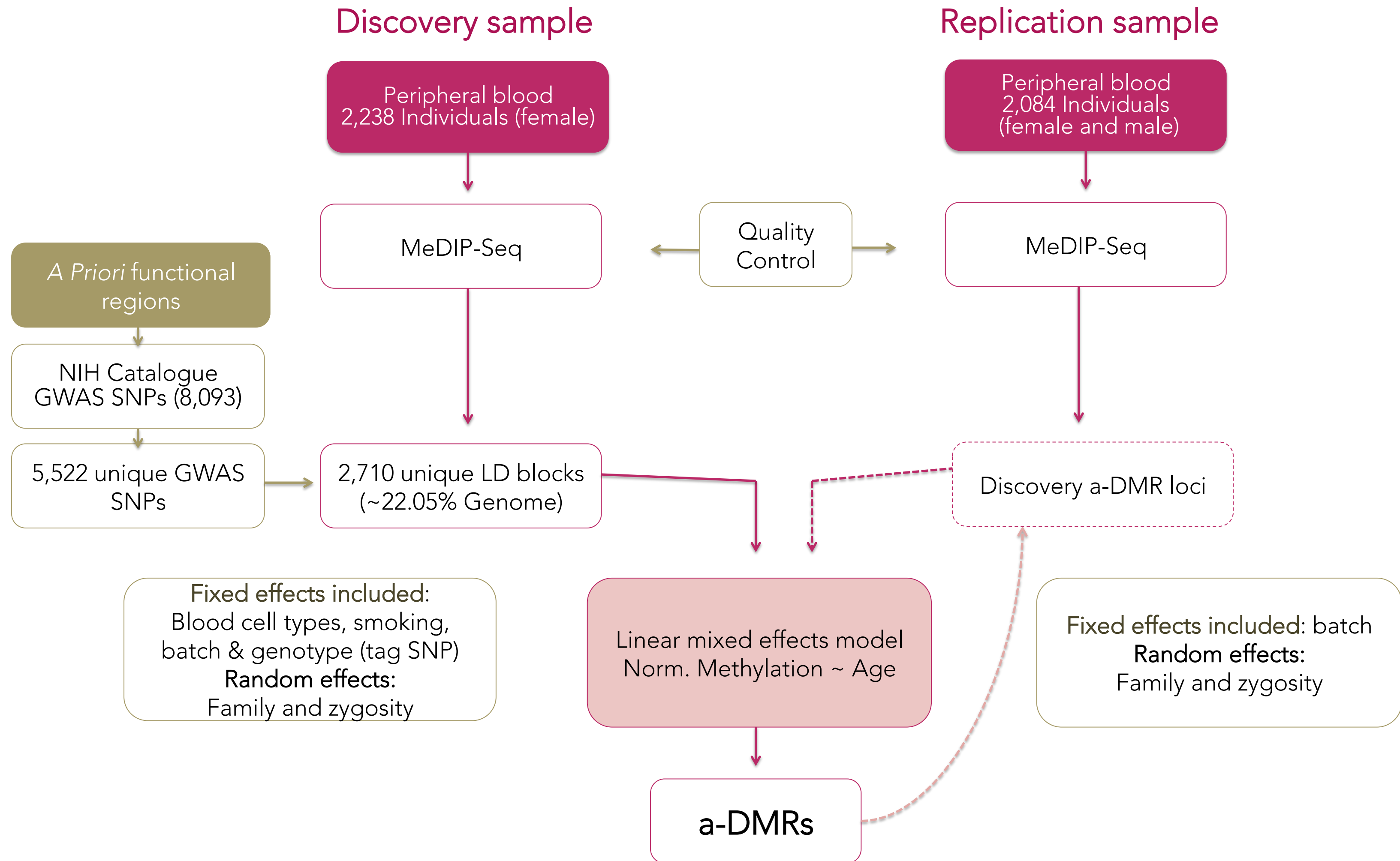

Supplement: Additional file 6: — Figure S4. Study design flowchart. (PDF 152 kb) [file 13059_2016_1051_MOESM6_ESM.pdf]
