# Supplementary material for: Novel regional age-associated DNA methylation changes within human common disease-associated loci
Source: Genome Biol. 2016 Sep 23;17:193. doi: 10.1186/s13059-016-1051-8 (PMC5034469; doi:10.1186/s13059-016-1051-8)
Supplement: Additional file 4: — Figure S2. All a-DMRs within (1) genomic location. Top: a-DMRs (purple), gene, DHS clusters, transcription factor ChIP-seq, ChromHMM segmentation, combined segmentation and conservation; and (2) scatterplot: x-axis = Age, y-axis = Normalised methylation. (PDF 34909 kb) [file 13059_2016_1051_MOESM4_ESM.pdf]

Supplementary Figure 2 – a-DMRs

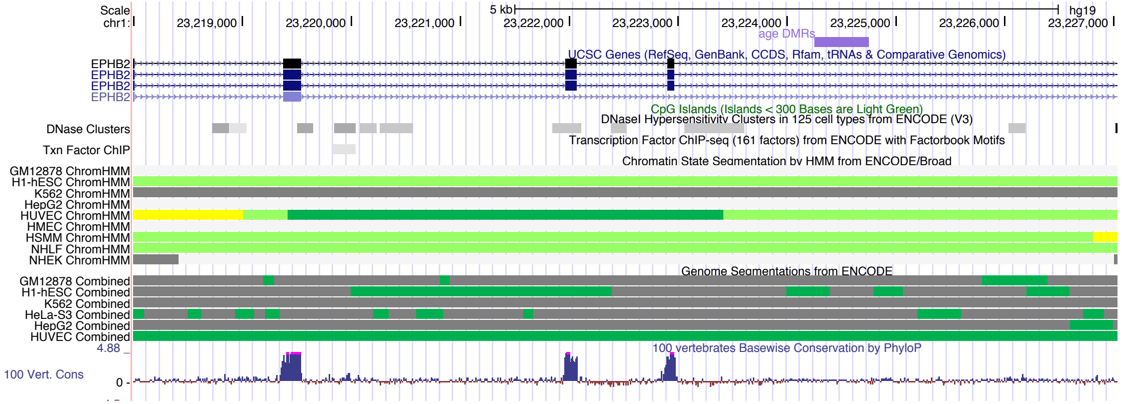

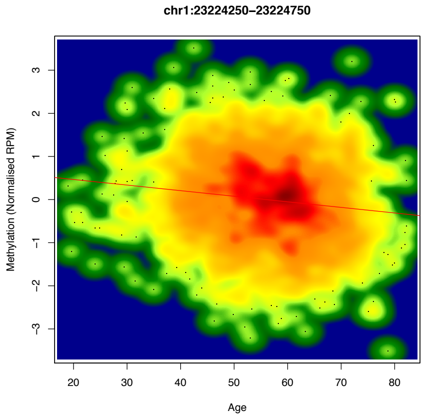

S1.1: *EPHB2* a-DMR

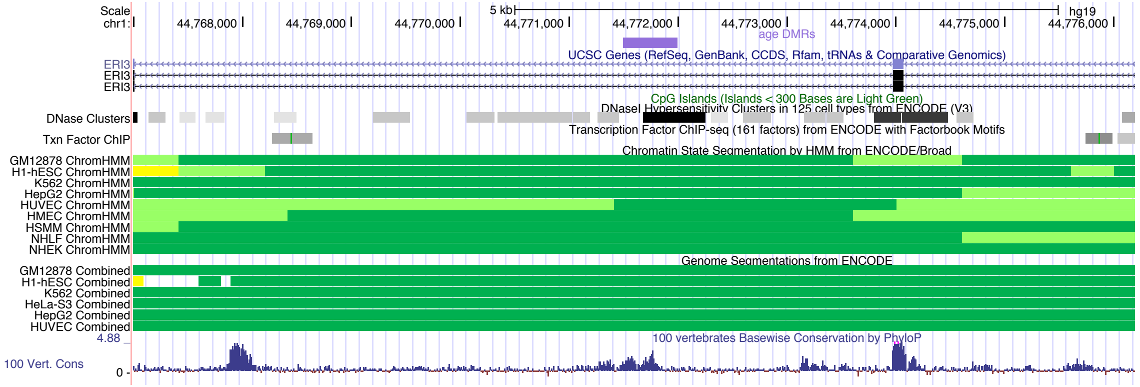

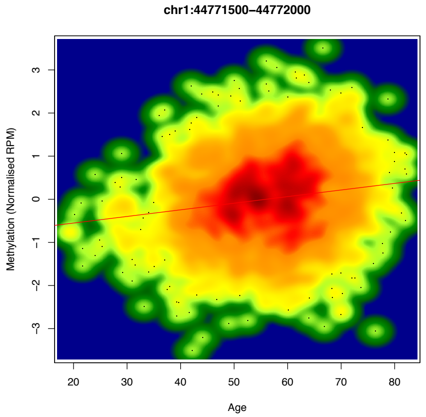

S1.2: *ERI3* a-DMR

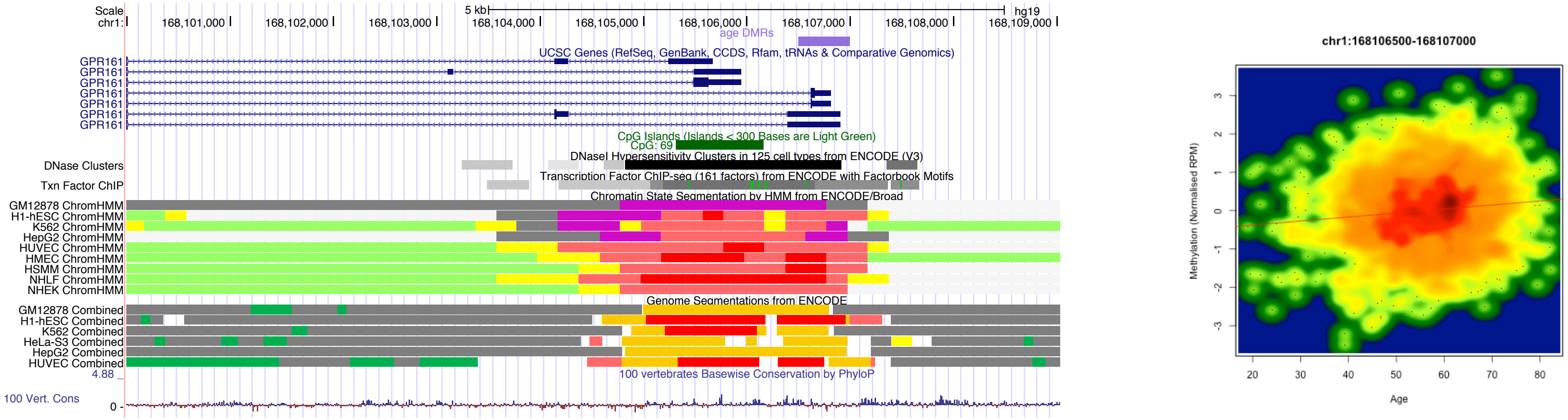

S1.3: *GPR161* a-DMR

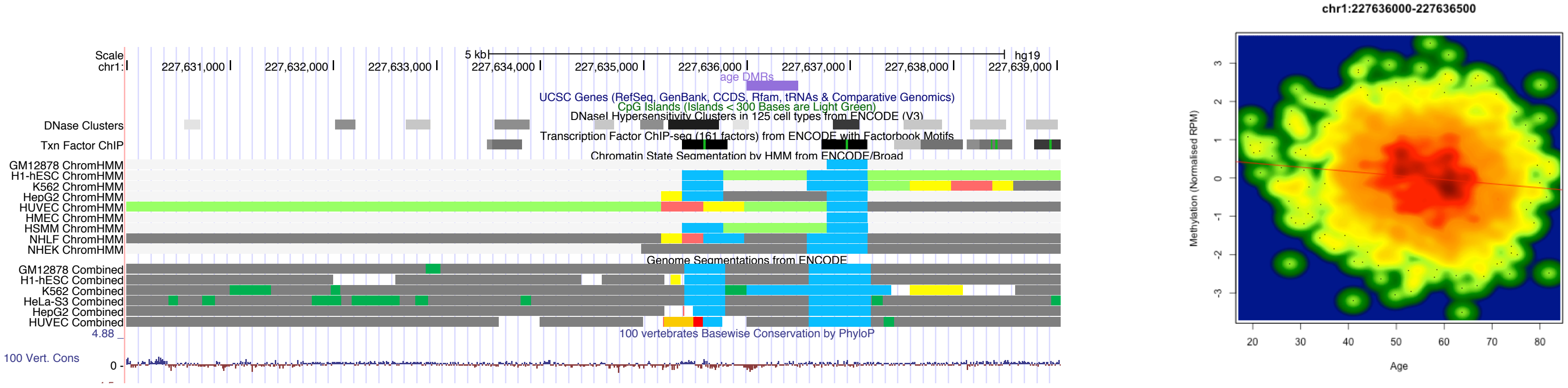

S1.4: upstream ZNF678 a-DMR

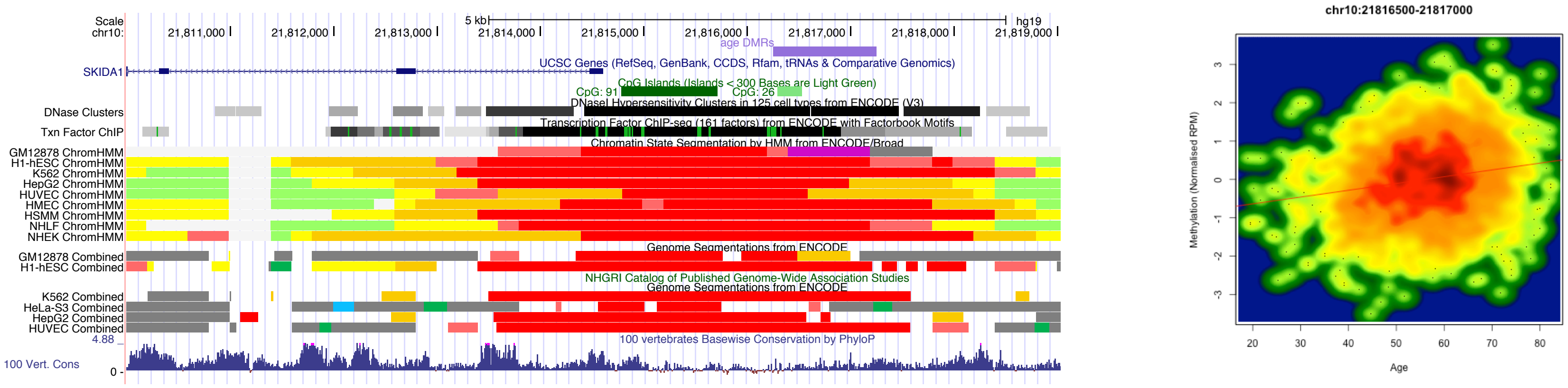

S1.5: *SKIDA1* a-DMR

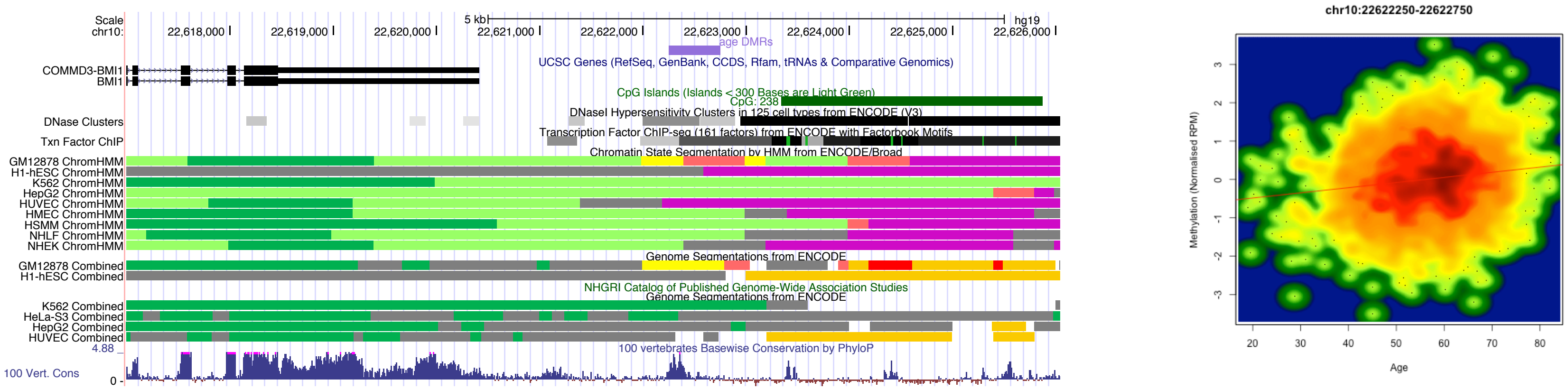

S1.6: *BMI1* a-DMR

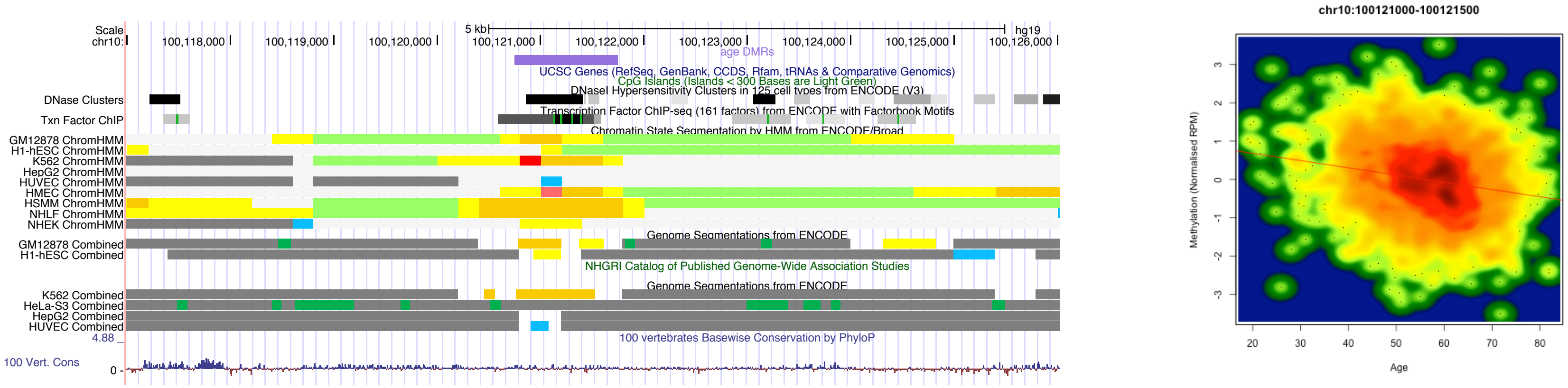

S1.7: downstream *PYROXD2*

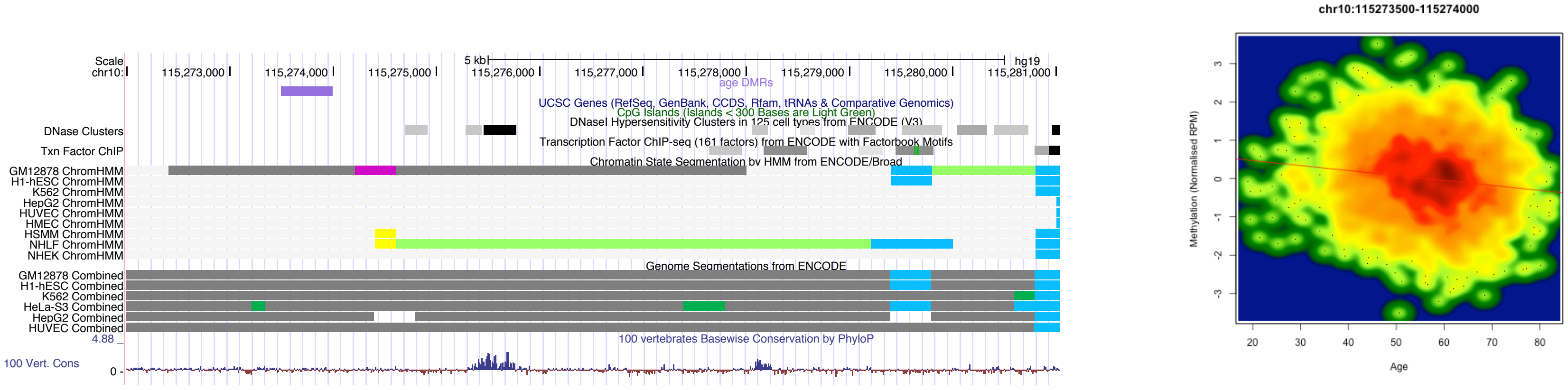

S1.8: upstream *HABP2* a-DMR

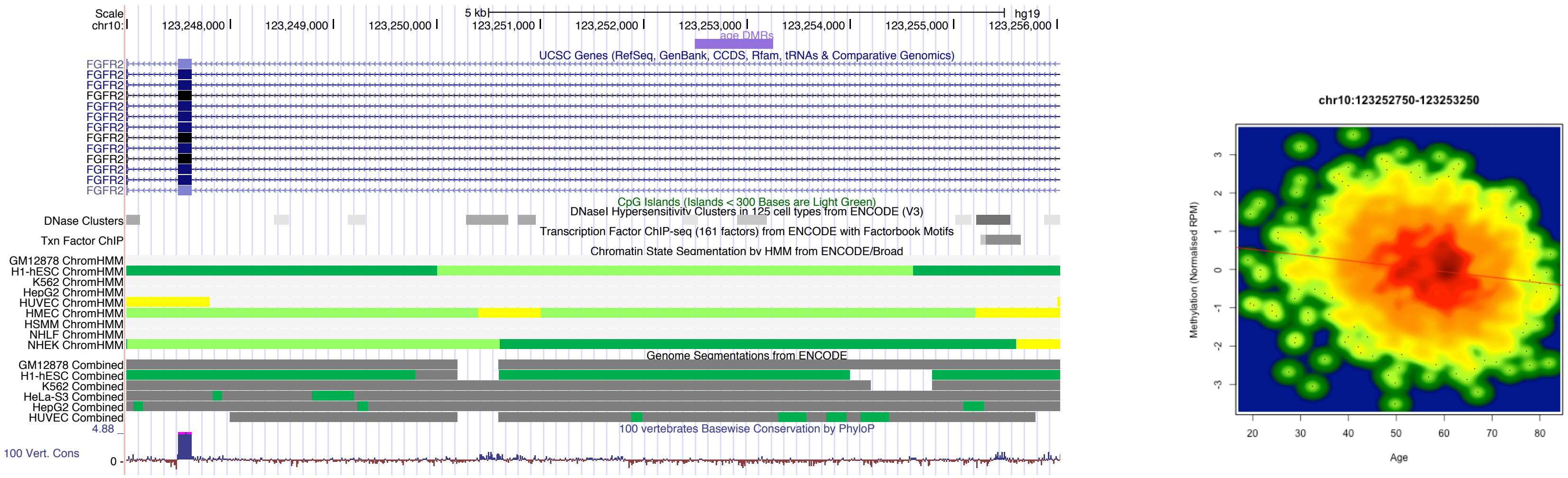

S1.9: *FGFR2* a-DMR

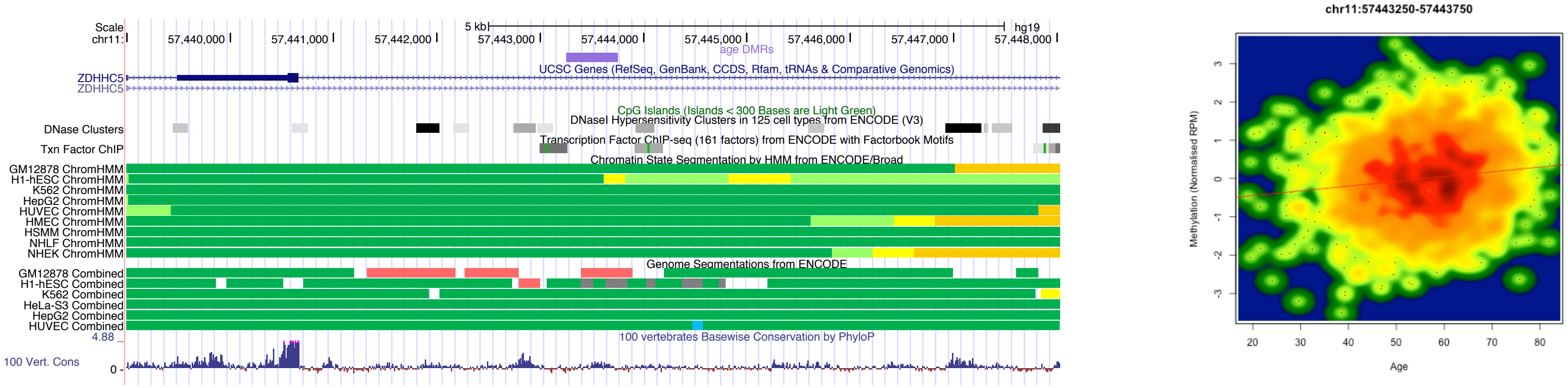

S1.10: *ZDHHC5* a-DMR

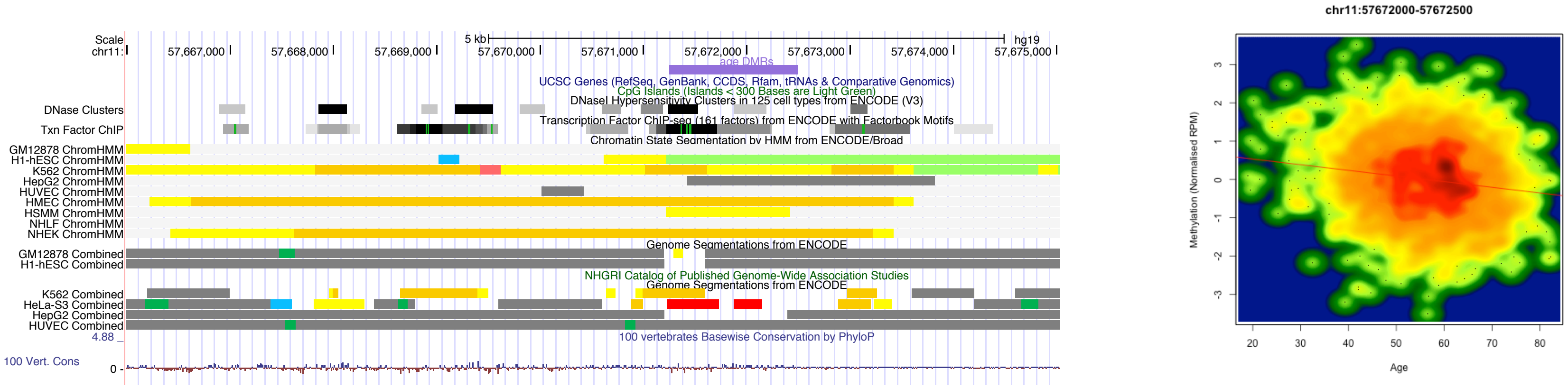

S1.11: intergenic *CTNND1 OR6Q1 I* a-DMR

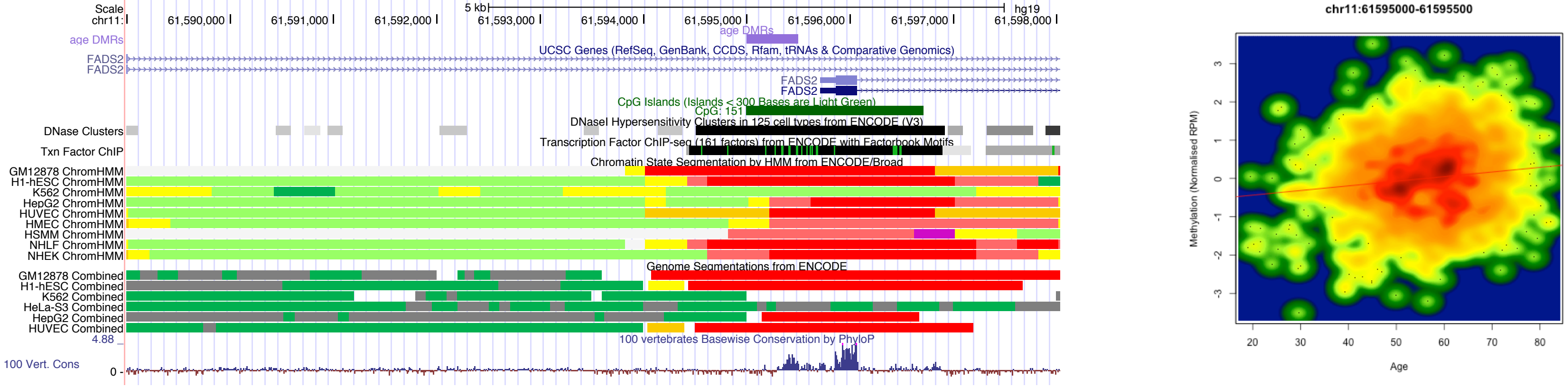

S1.12: *FADS2* a-DMR

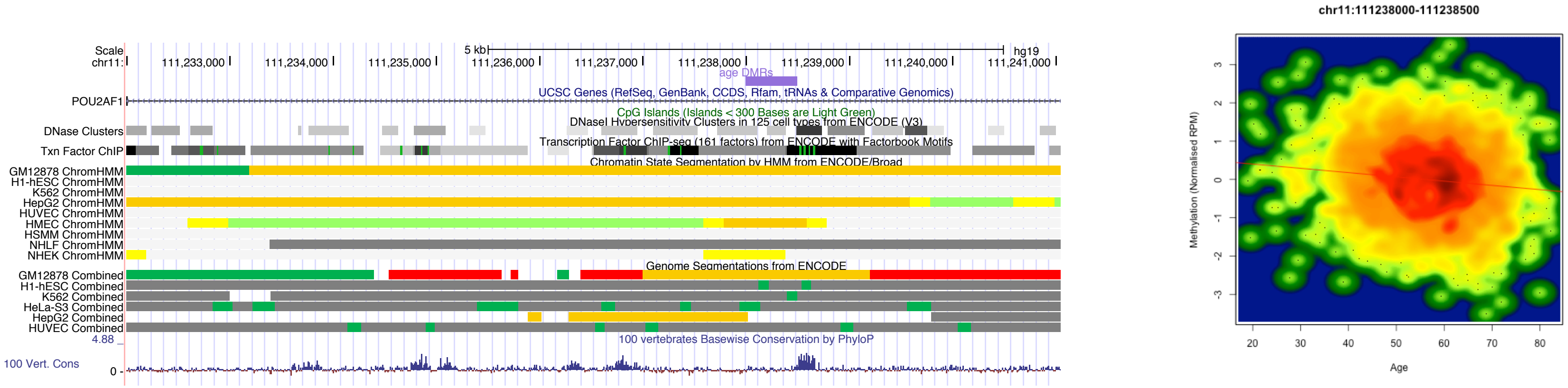

Fig S1.13 *POU2AF1* a-DMR

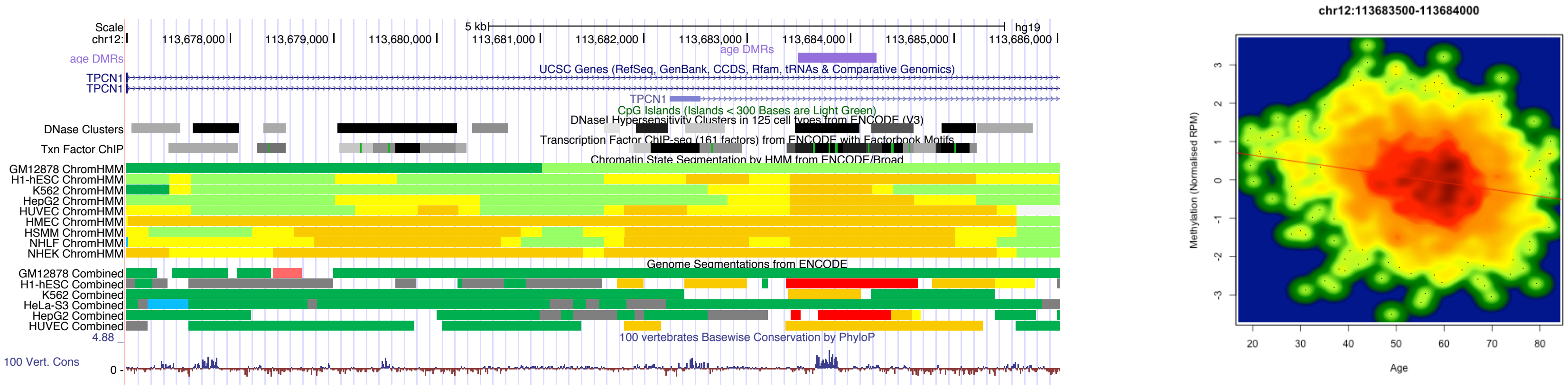

Fig S1.14: *TPCN1* a-DMR

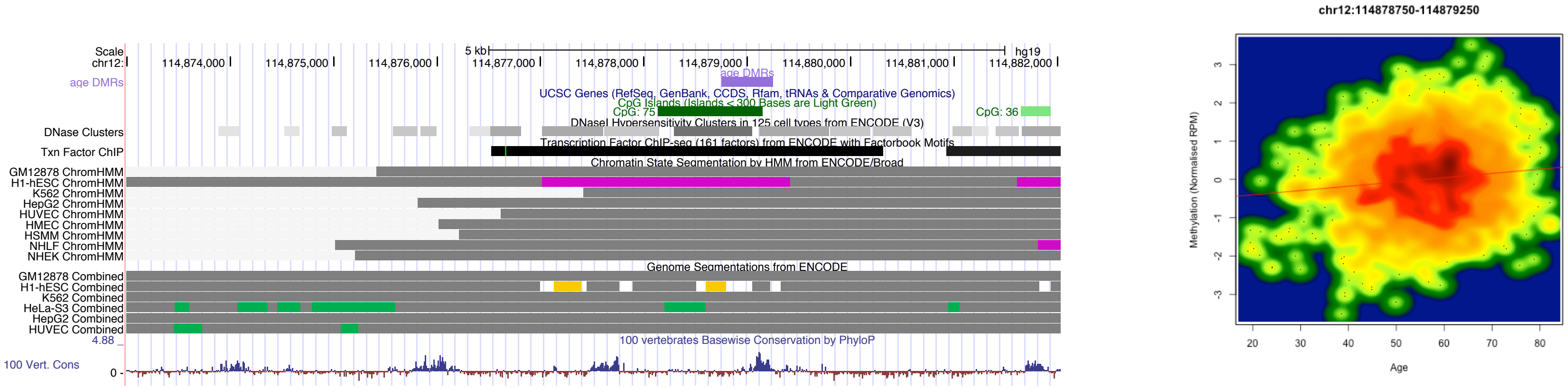

Fig S1.15: upstream *TBX5* a-DMR

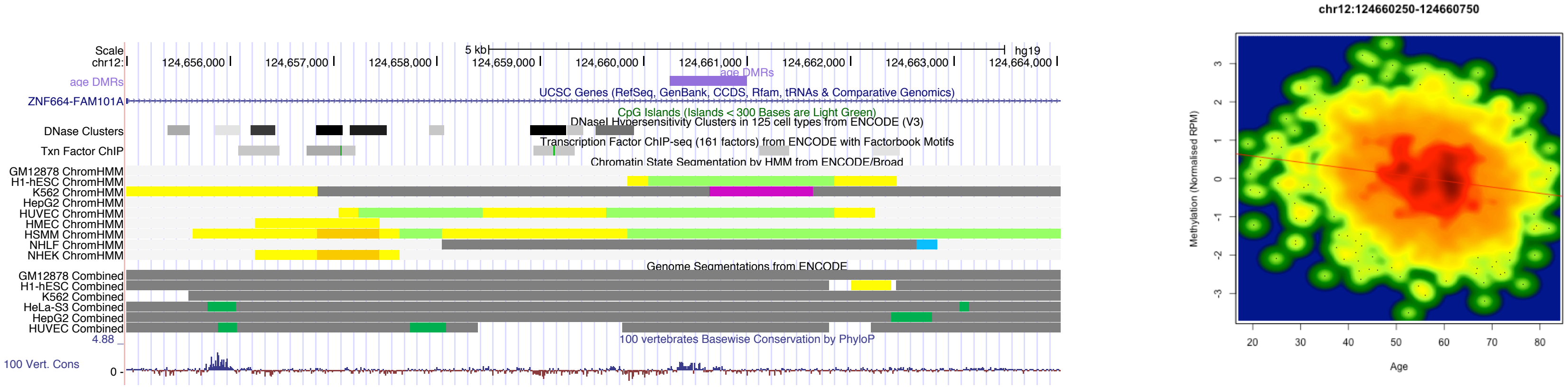

Fig S1.16: *ZNF664-FAM101A* a-DMR

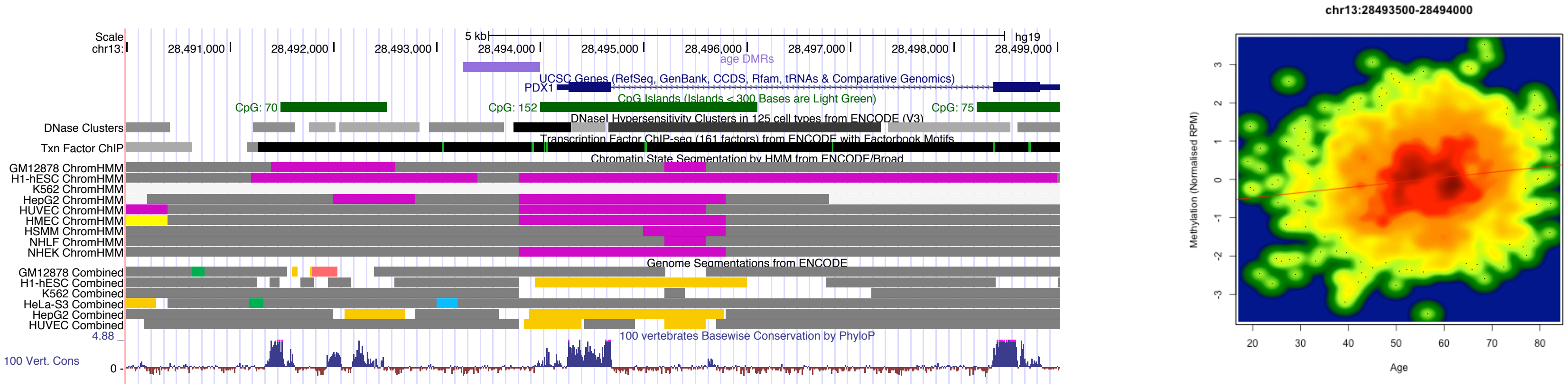

Fig S1.17: *PDX1* a-DMR

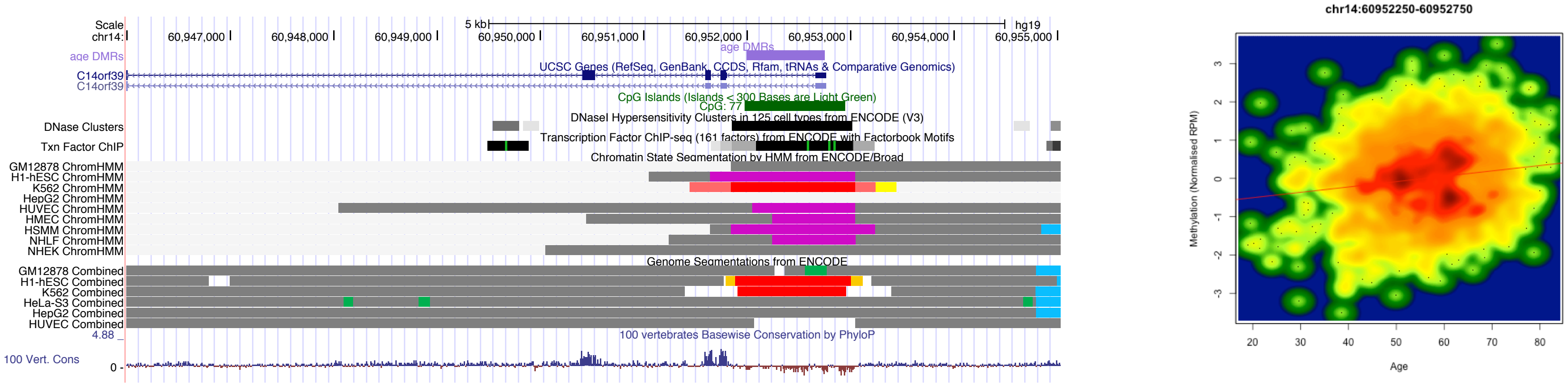

Fig S1.18: *C14orf39* a-DMR

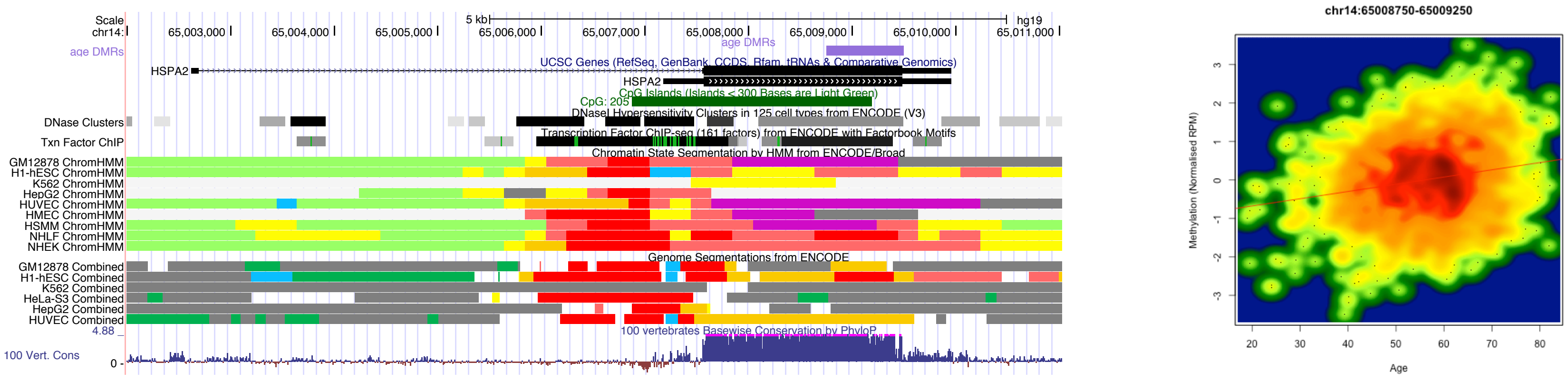

Fig S1.19: *HSPA2* a-DMR

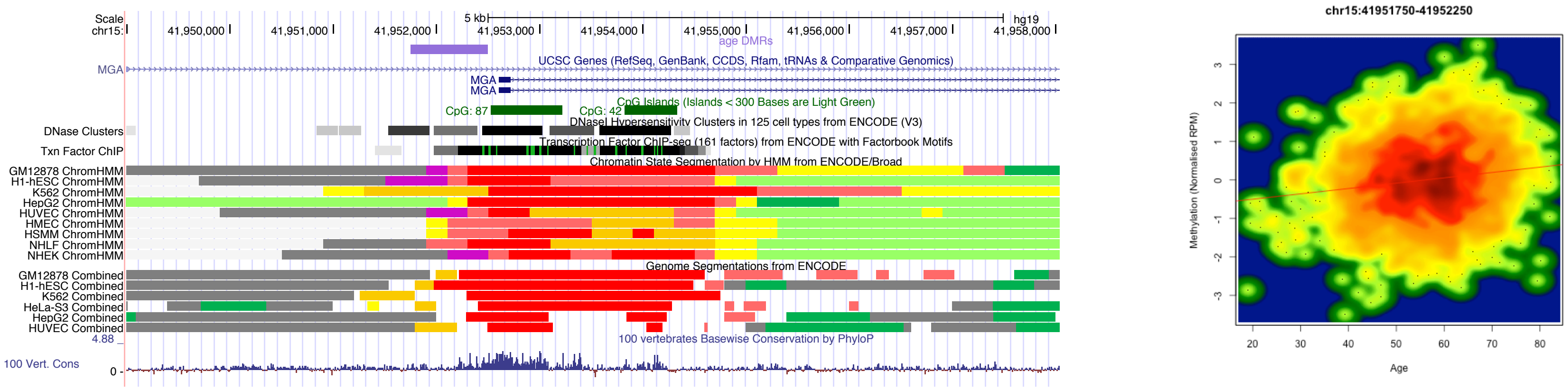

Fig S1.20: *MGA* a-DMR

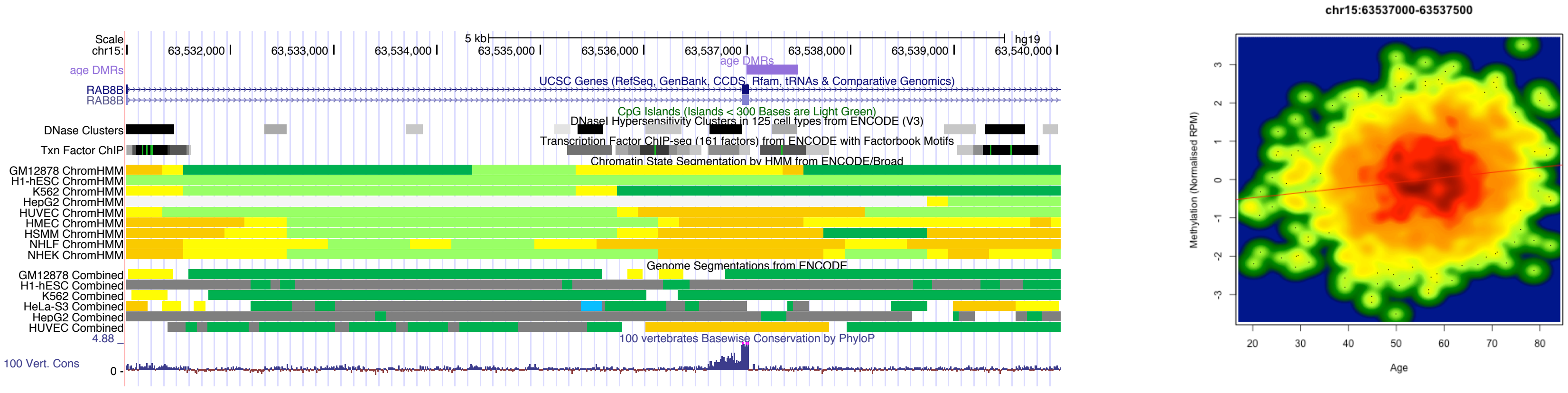

Fig S1.21: *RAB8B* a-DMR

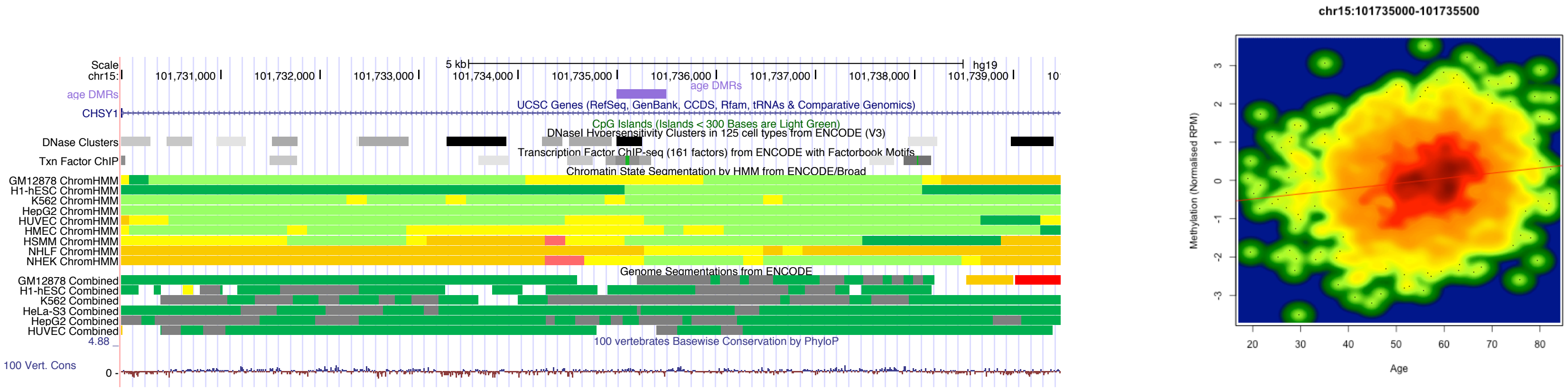

Fig S1.22: *CHSY1* a-DMR

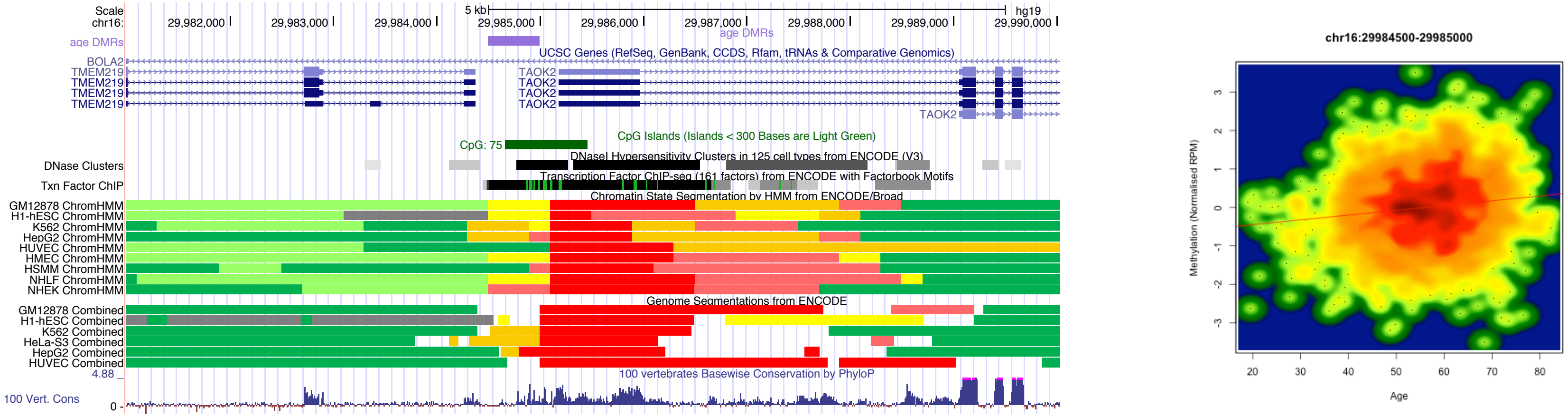

Fig S1.23: *TAOK2* a-DMR

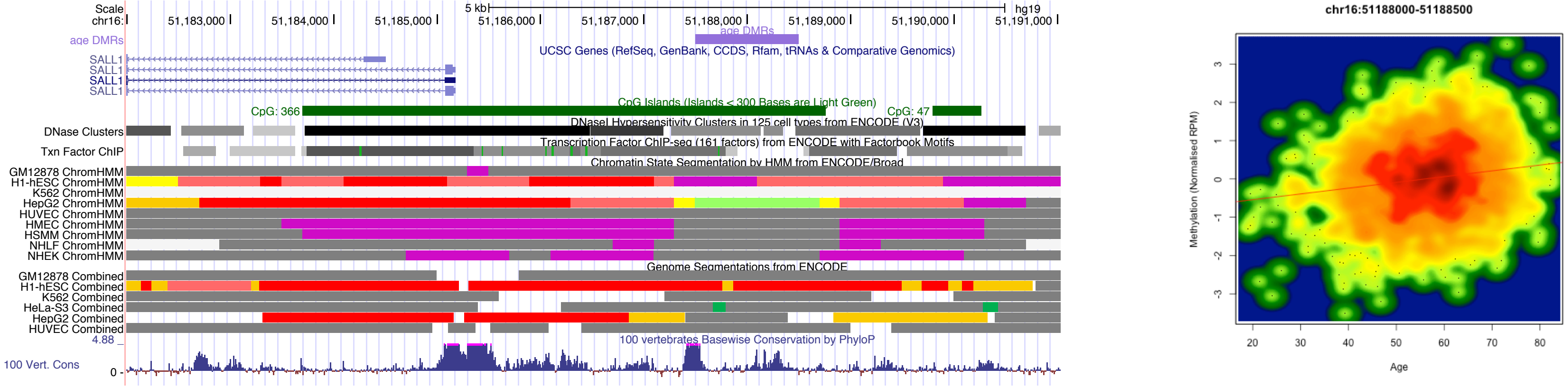

Fig S1.24: *SALL1* a-DMR

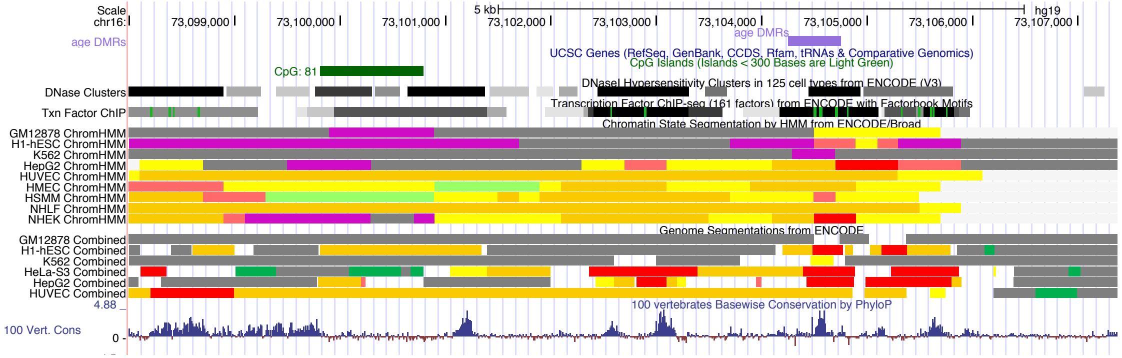
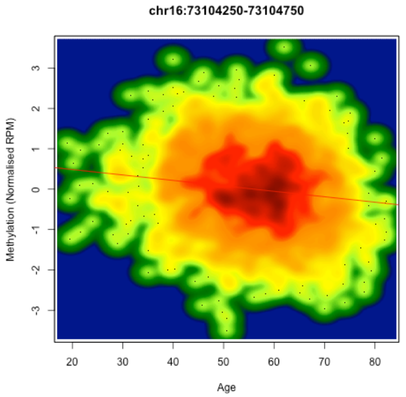

Fig S1.25: upstream *ZFHX3* a-DMR

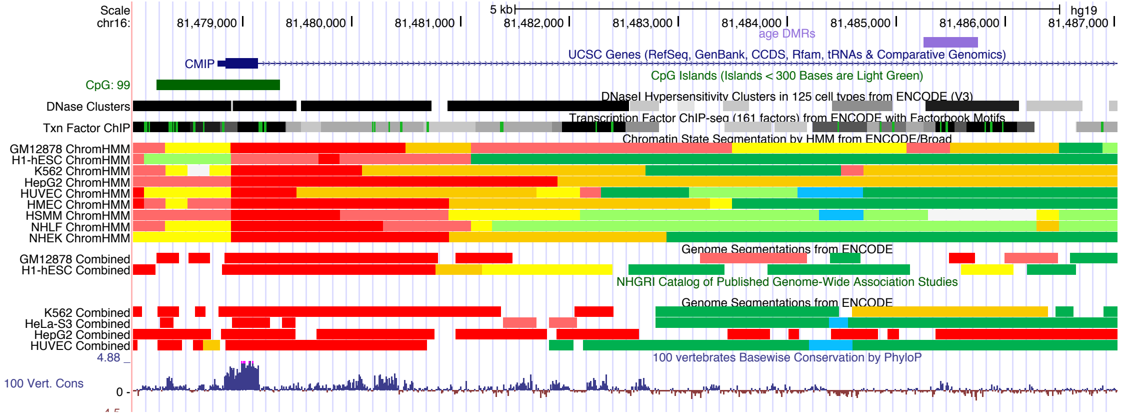
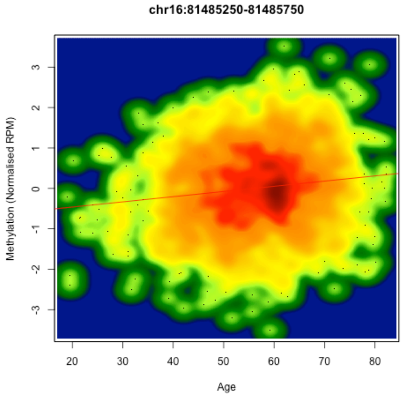

Fig S1.26: *CMIP* a-DMR

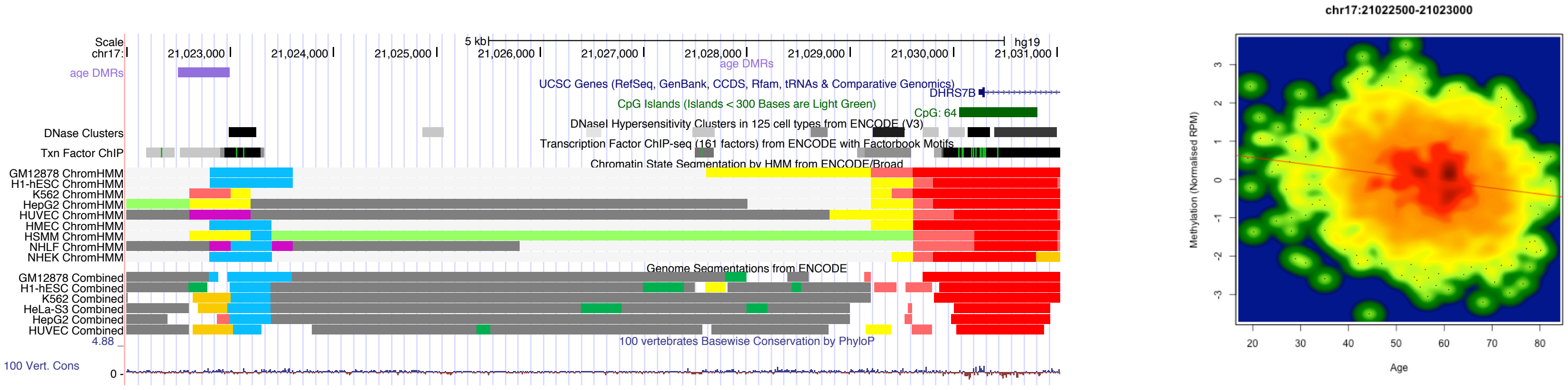

Fig S1.27: upstream *DHRS7B* a-DMR

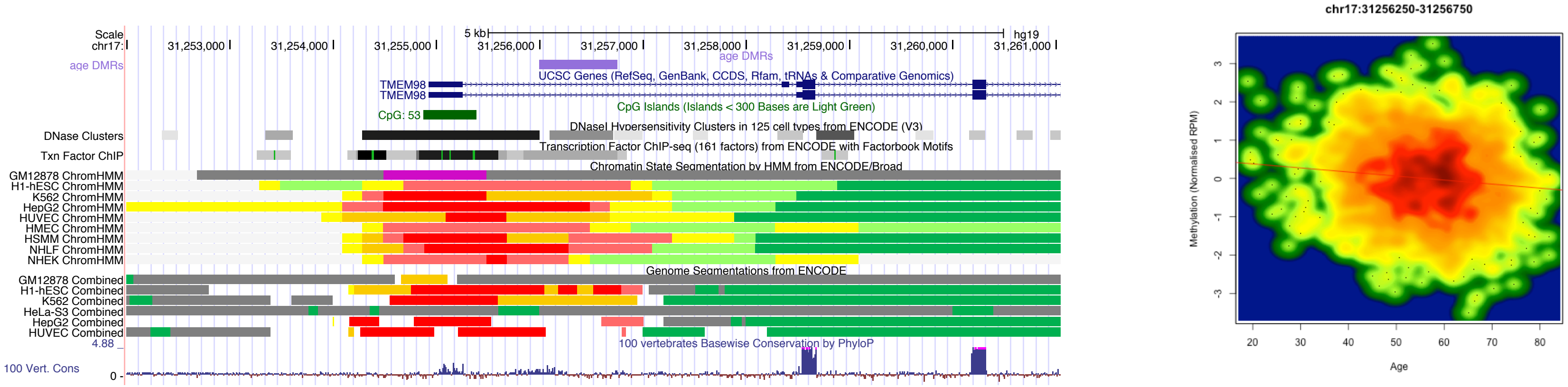

Fig S1.28: *TMEM98* a-DMR

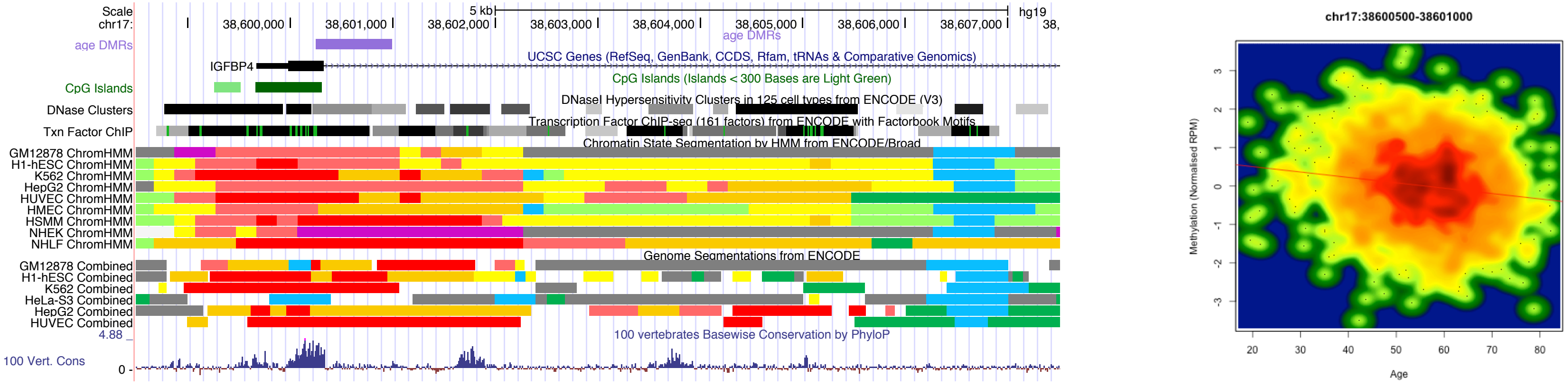

Fig S1.29: *IGFBP4* a-DMR

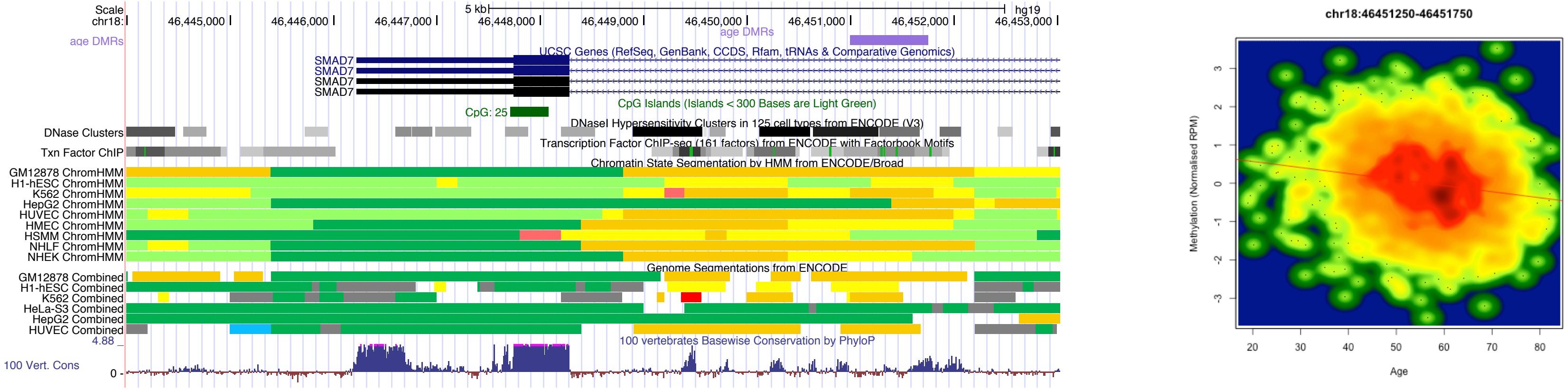

Fig S1.30: *SMAD7* a-DMR

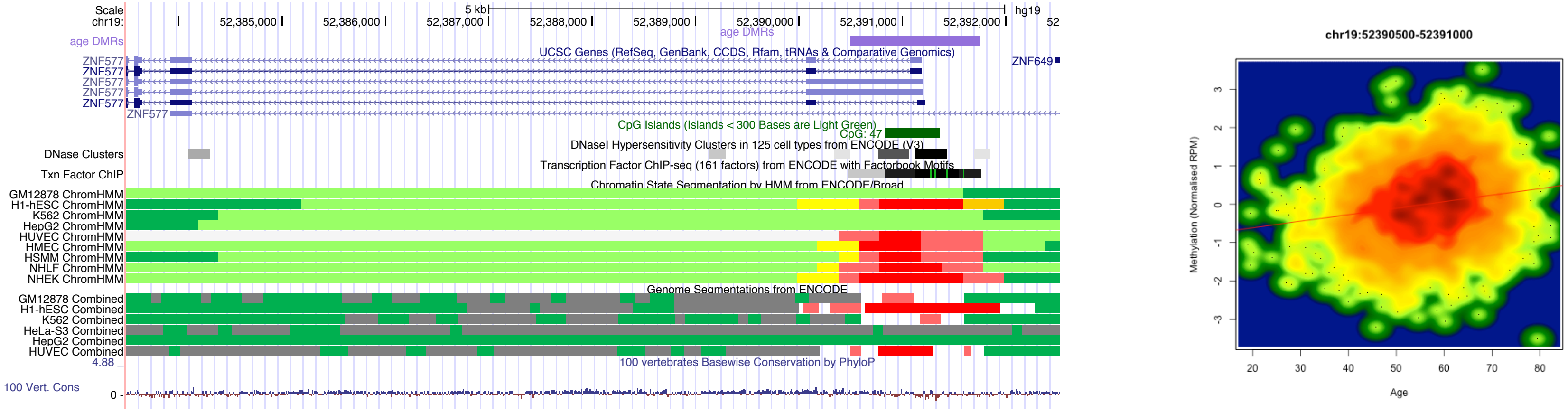

Fig S1.31: *ZNF577* a-DMR

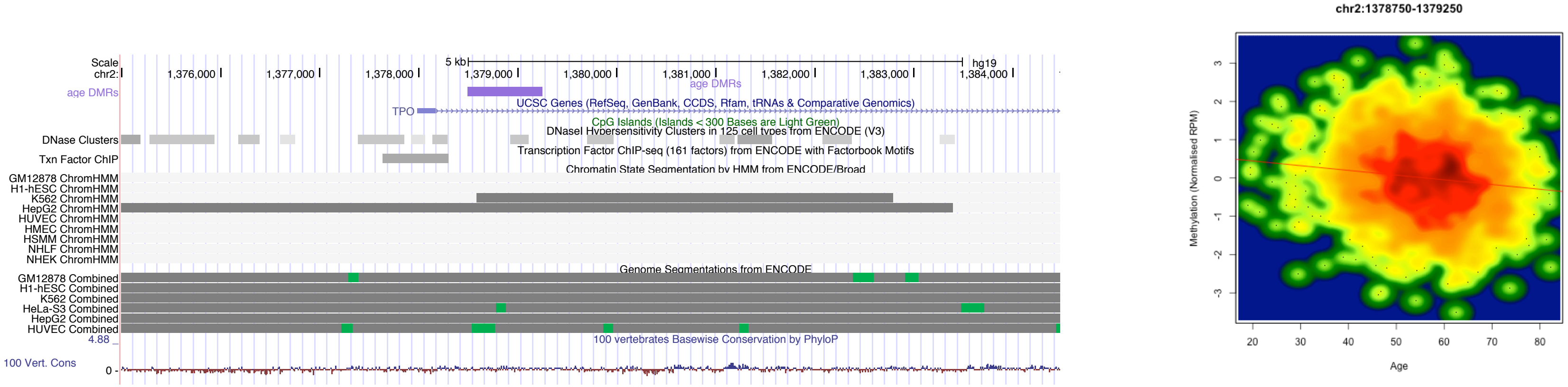

S1.32: *TPO* a-DMR

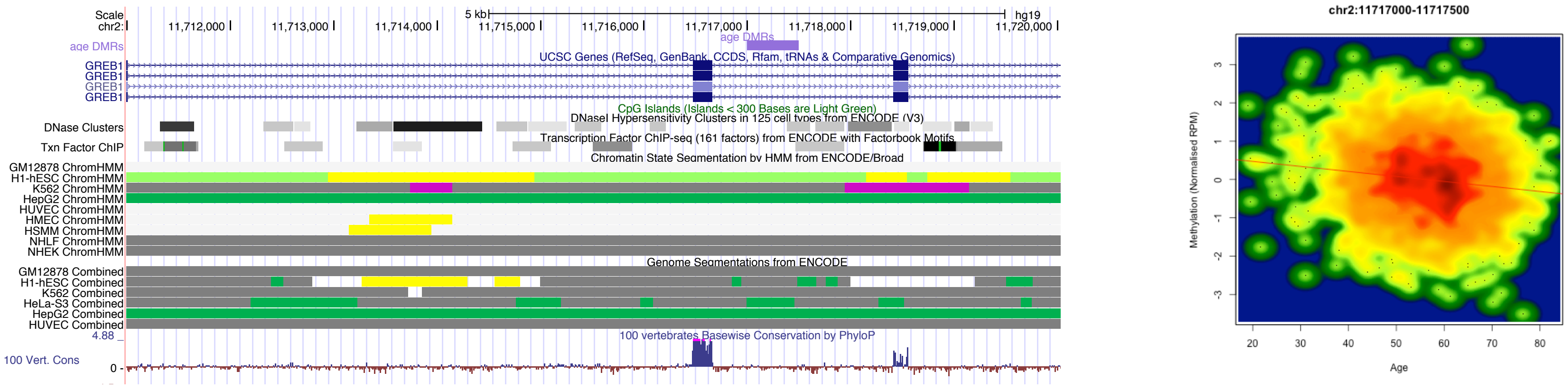

S1.33: *GREB1* a-DMR

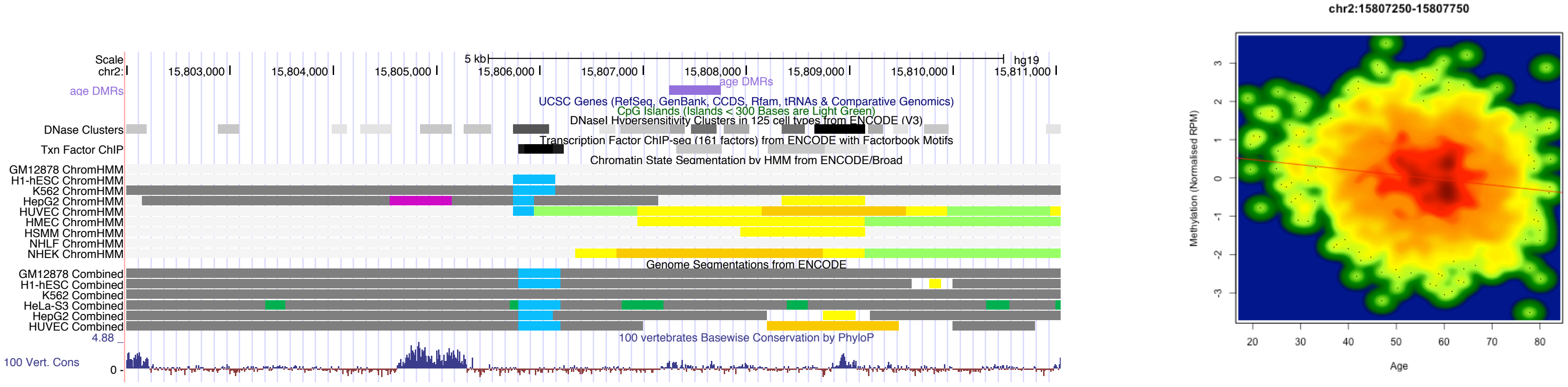

S1.34: downstream *DDX1* a-DMR

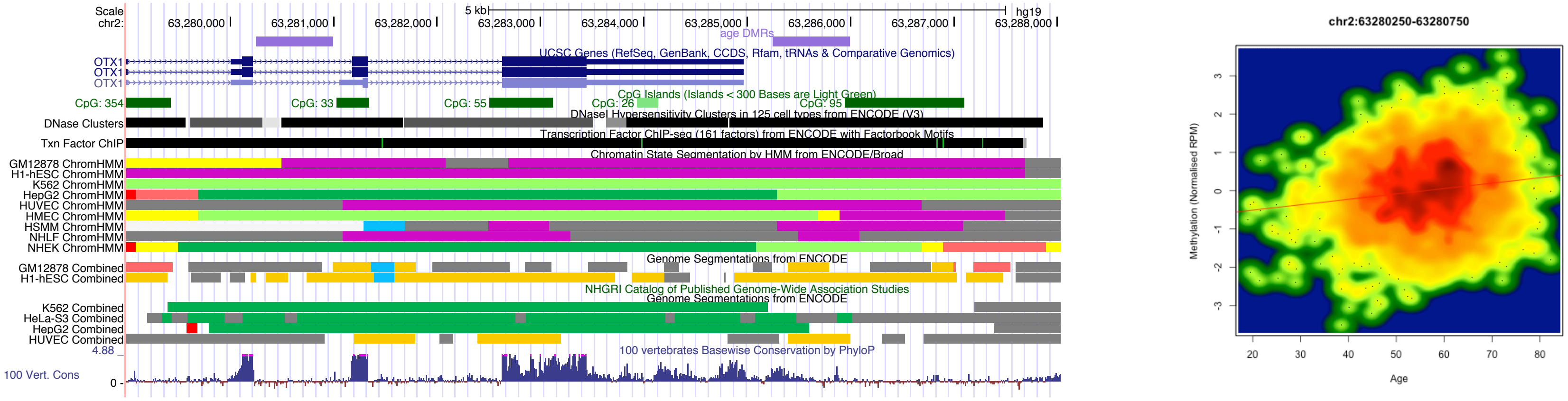

Fig S1.35: *OTX1* a-DMR [1]

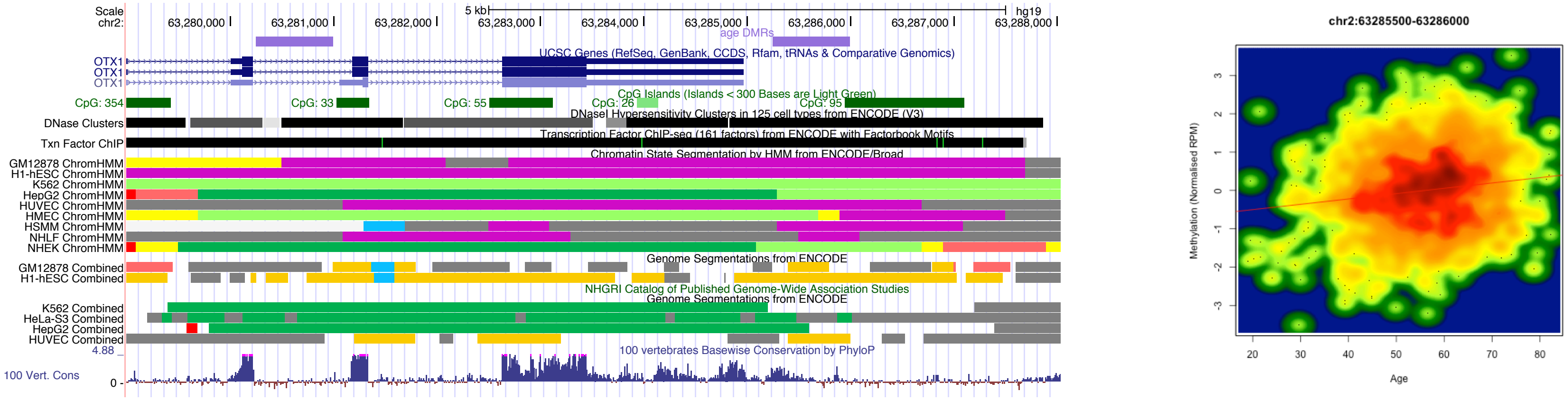

Fig S1.36: *OTX1* a-DMR [2]

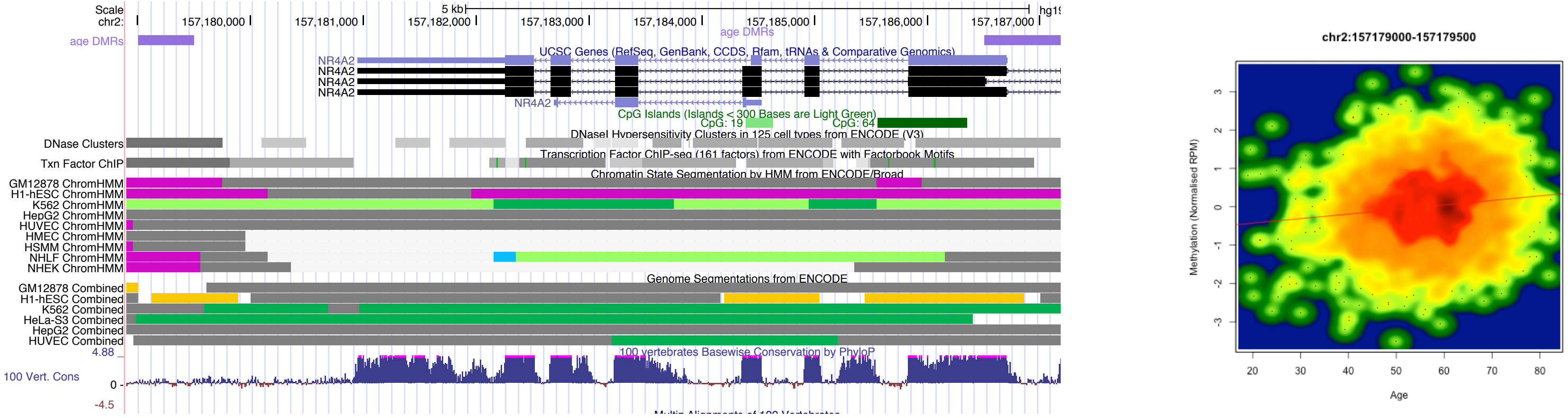

Fig S1.37: *NR4A2* a-DMR [1]

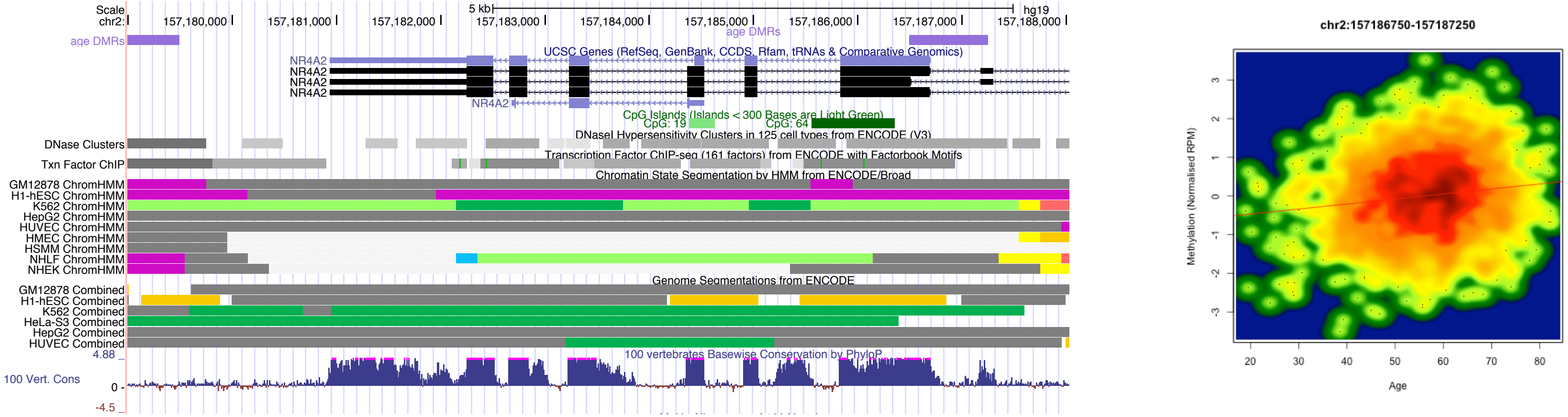

Fig S1.38: *NR4A2* a-DMR [2]

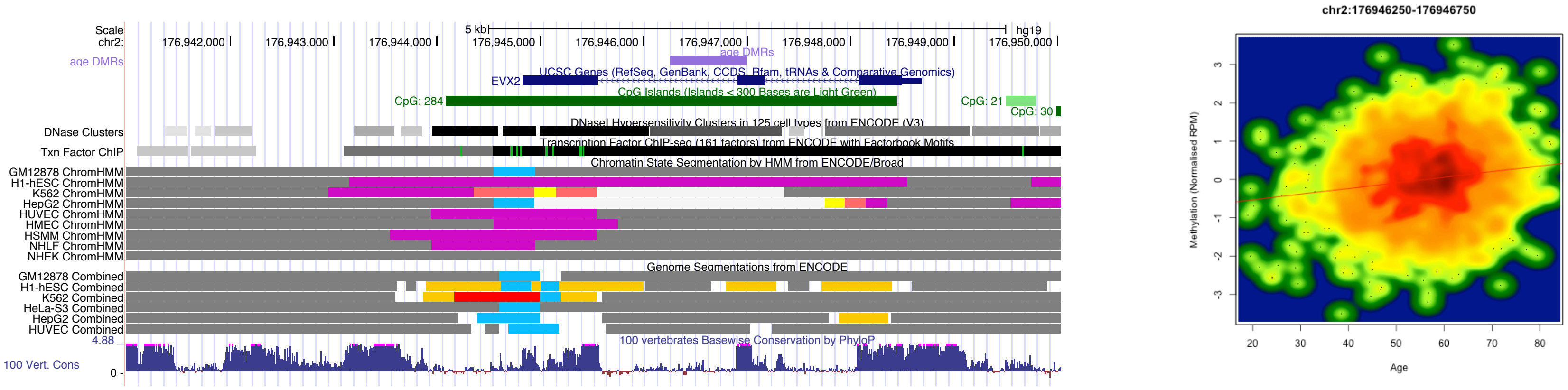

Fig S1.39: *EVX2* a-DMR

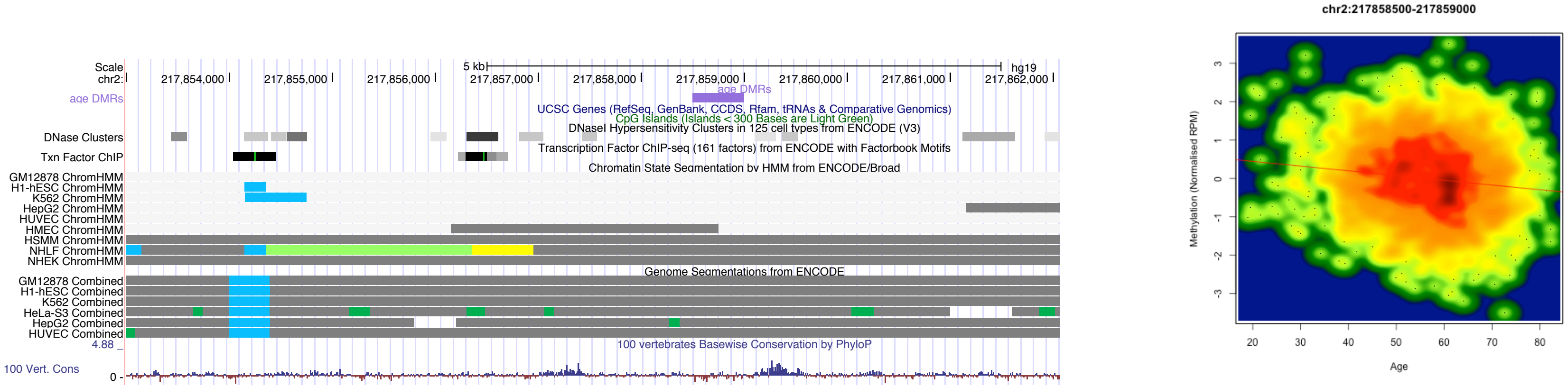

Fig S1.40: unknown chr2 a-DMR

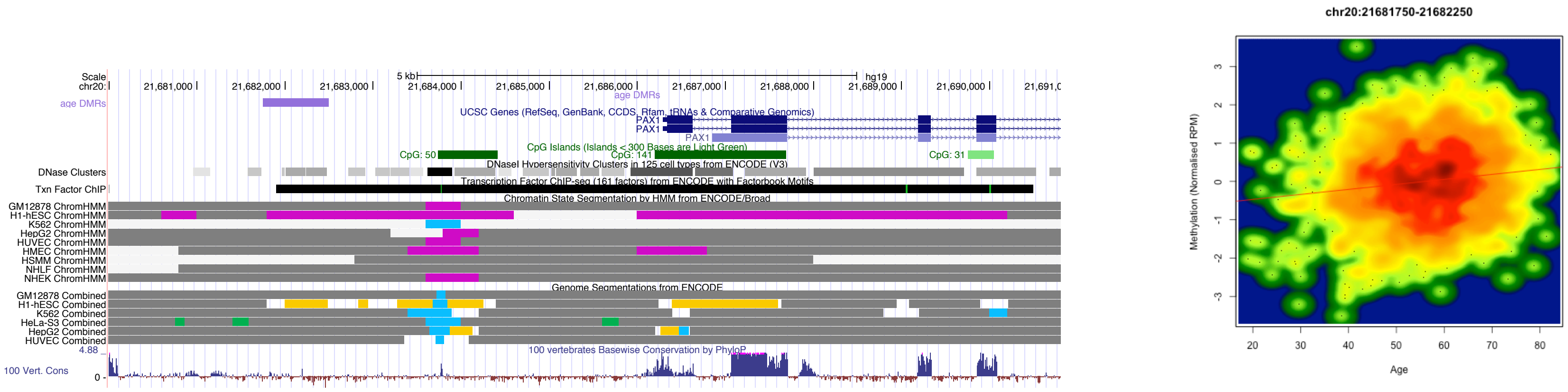

Fig S1.41: upstream *PAX1* a-DMR

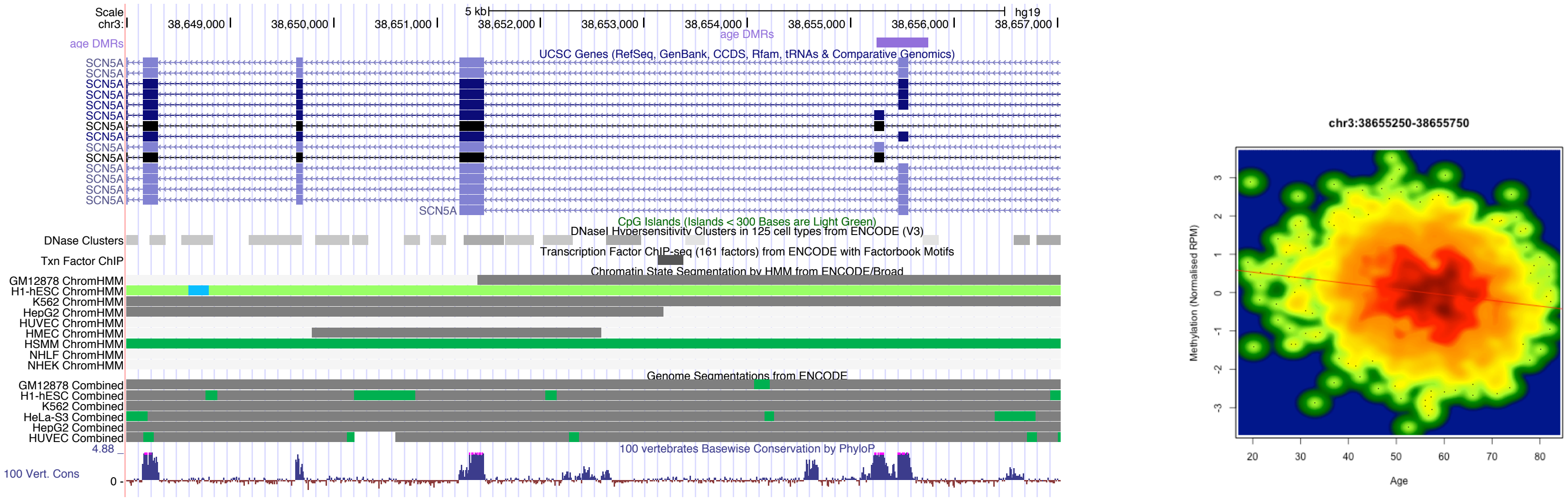

Fig S1.42: *SCN5A* a-DMR

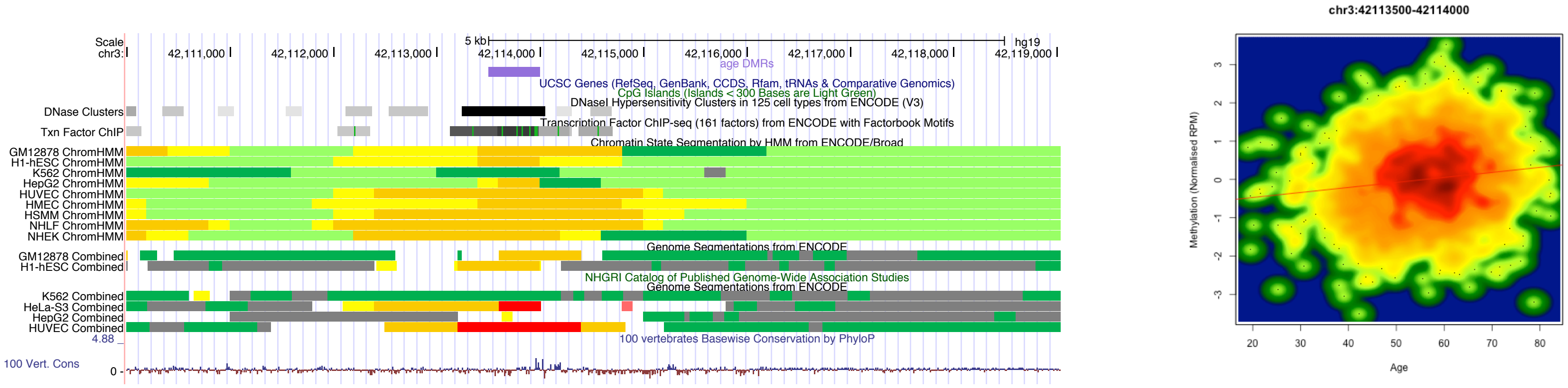

Fig S1.43: upstream *TRAK1* a-DMR

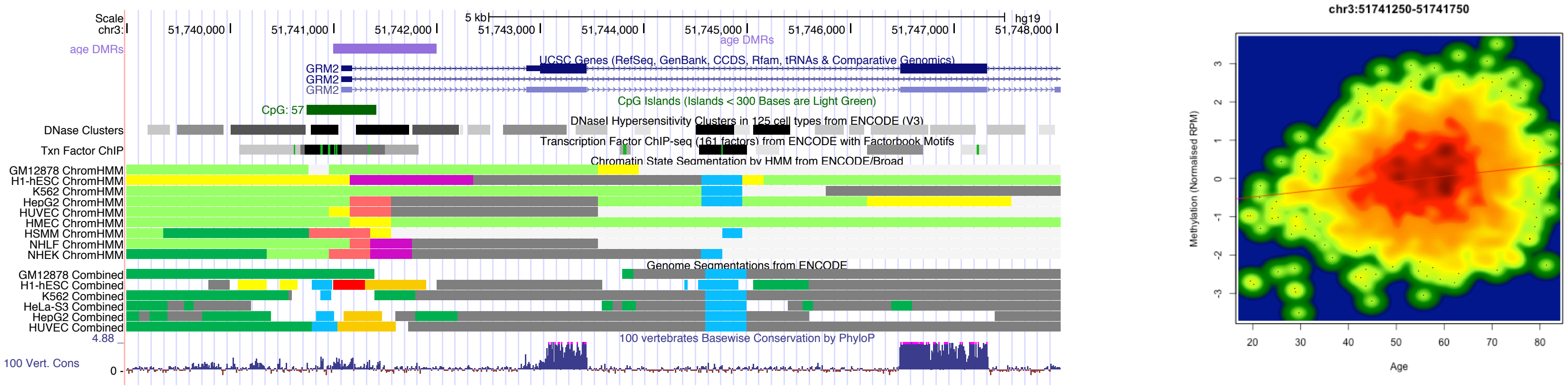

Fig S1.44: *GRM2* a-DMR

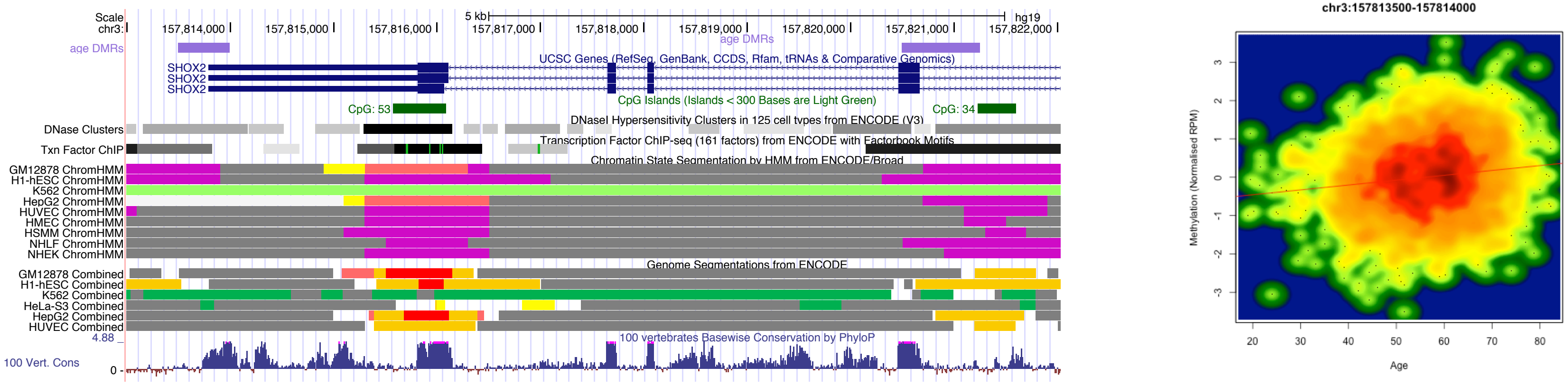

Fig S1.45: *SHOX2* a-DMR [1]

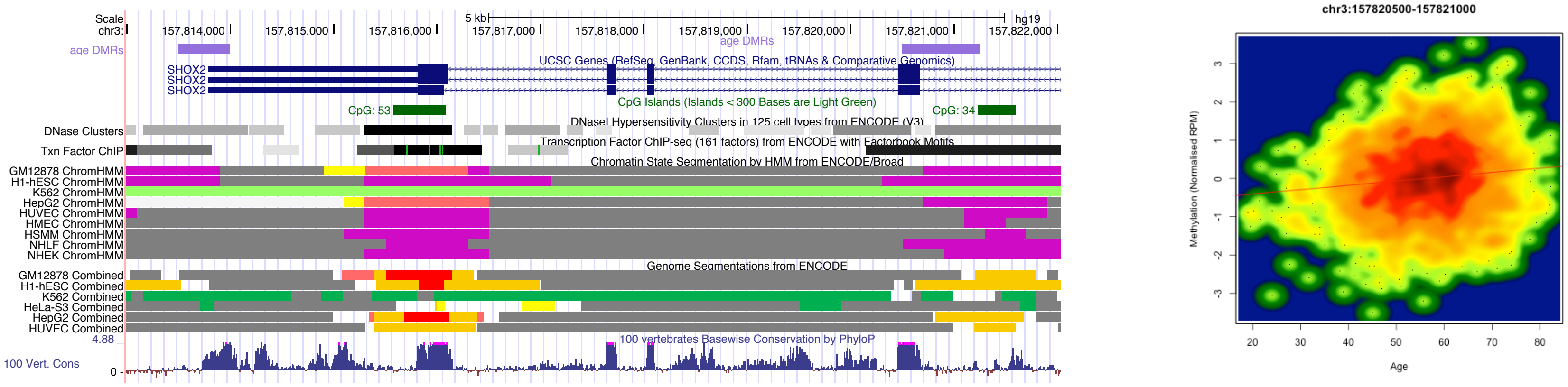

Fig S1.46: *SHOX2* a-DMR [2]

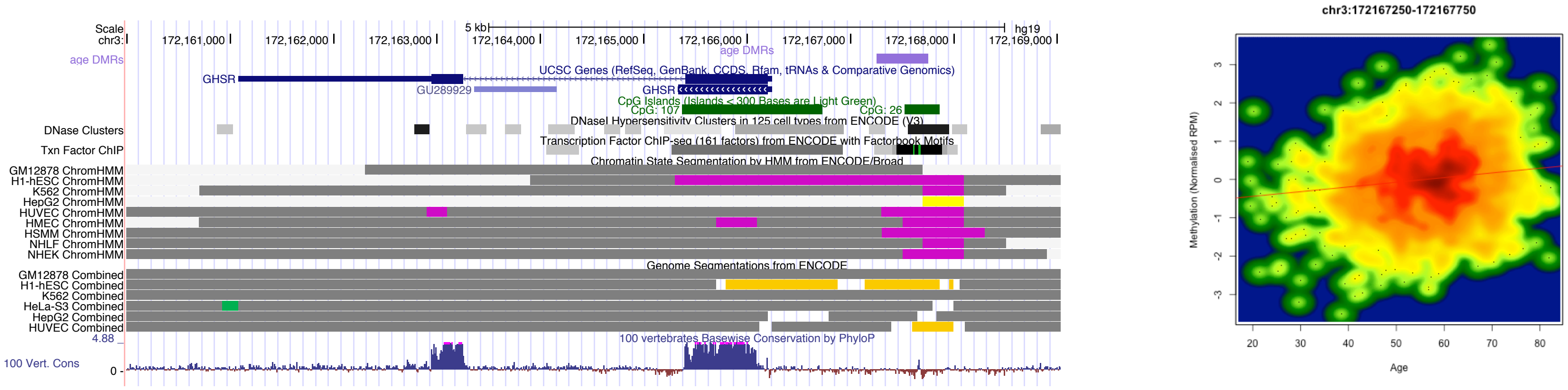

Fig S1.47: *GHSR* a-DMR

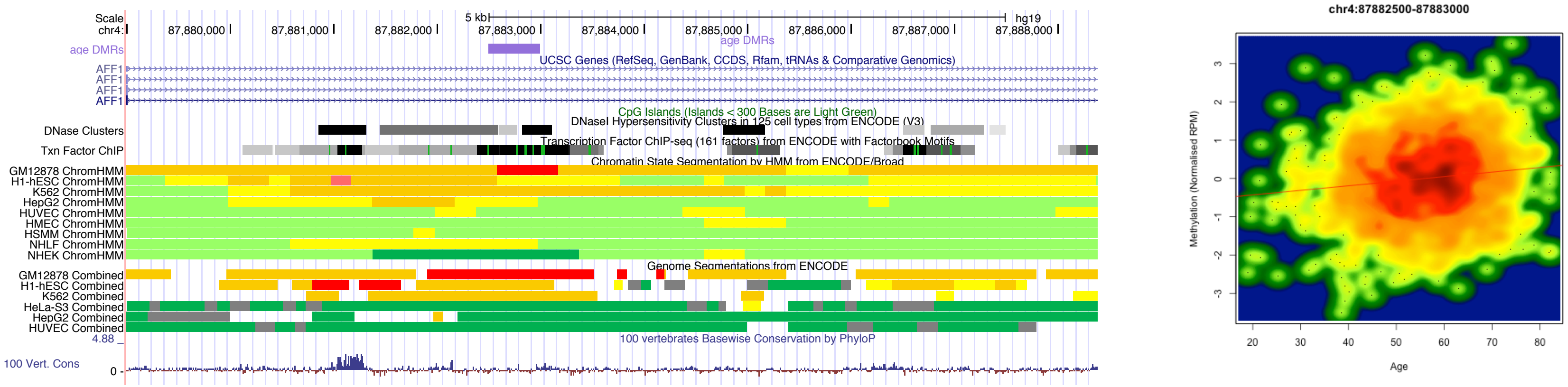

Fig S1.48: *AFF1* a-DMR

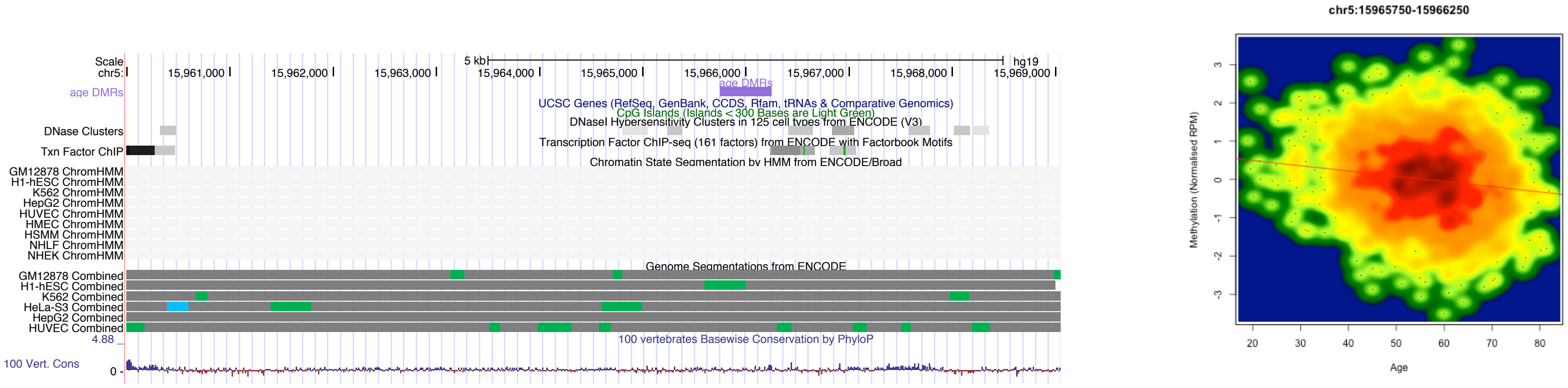

Fig S1.49: downstream *FBXL7* a-DMR

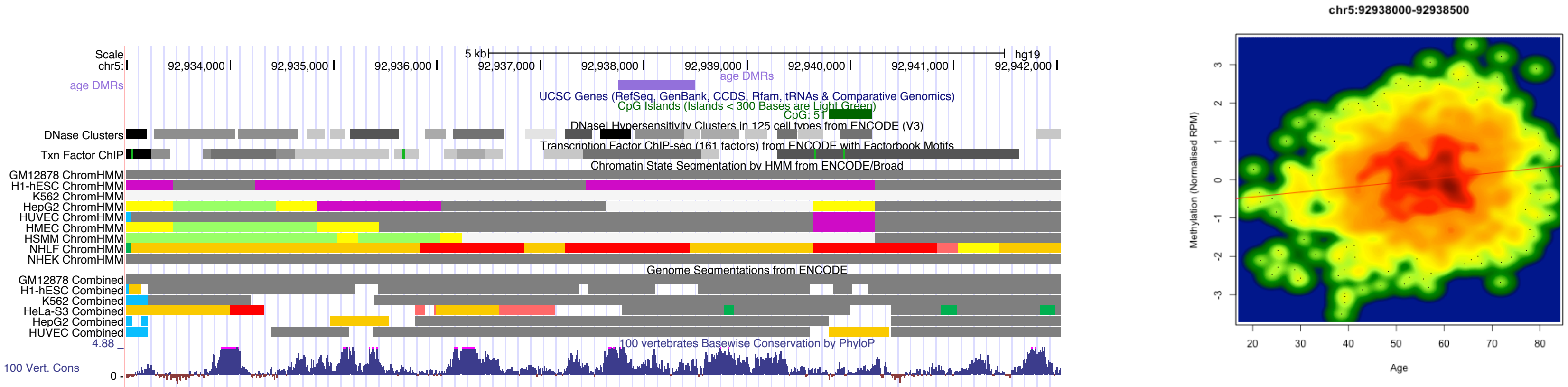

Fig S1.50: intergenic *NR2F1 FAM172A* a-DMR

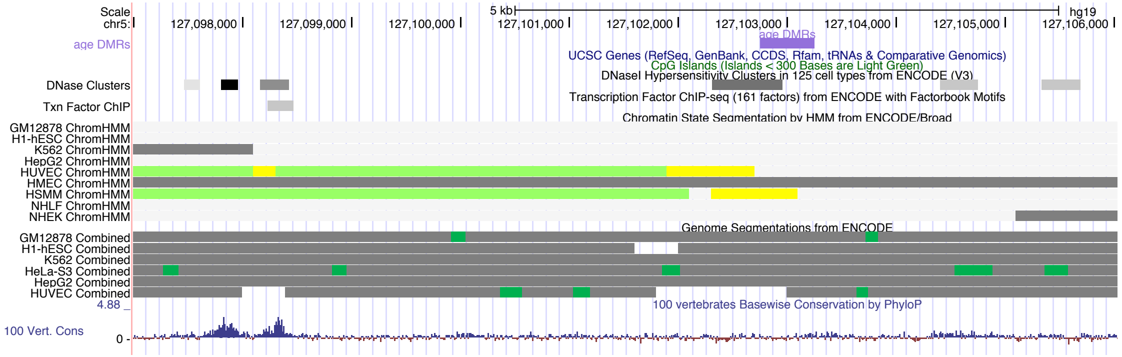

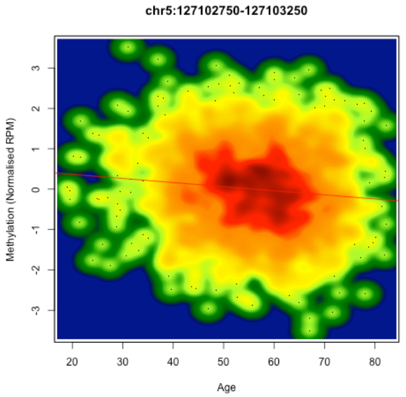

Fig S1.51: downstream *CTXN3* a-DMR

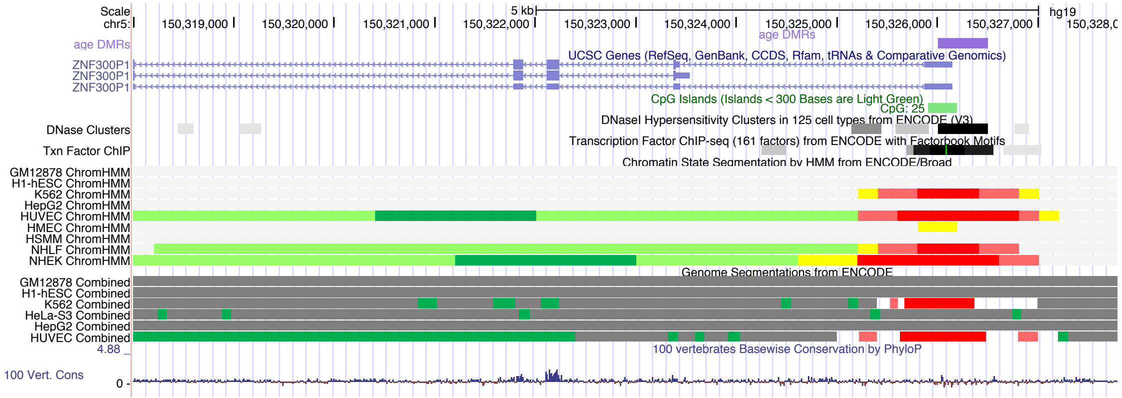

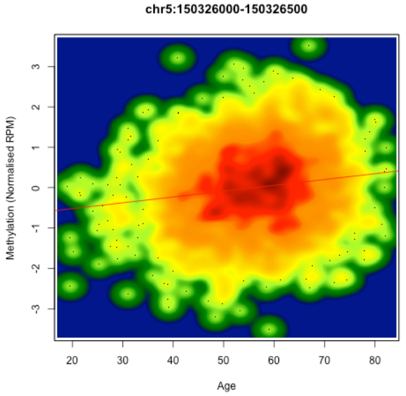

Fig S1.52: *ZNF300P1* a-DMR

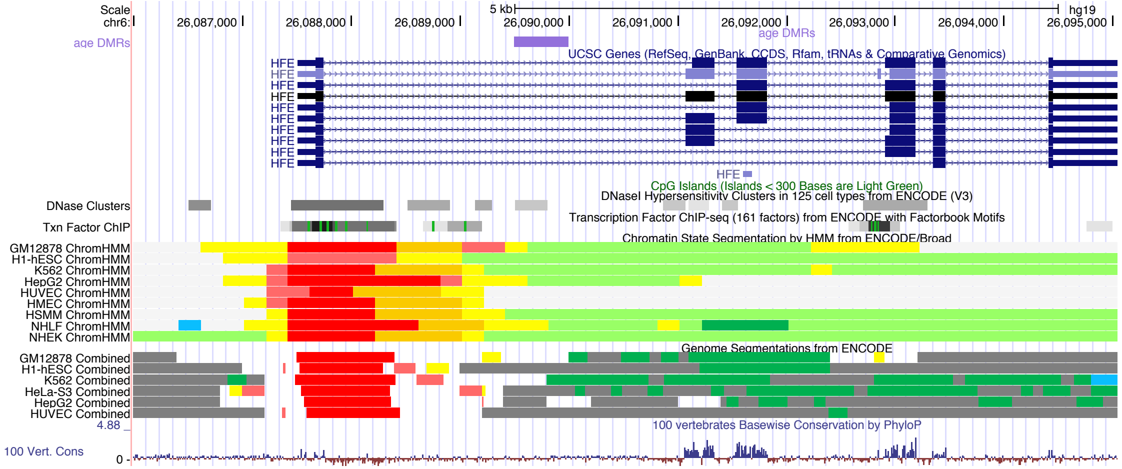

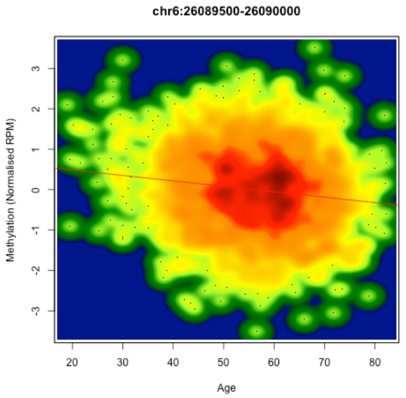

Fig S1.53: *HFE* a-DMR

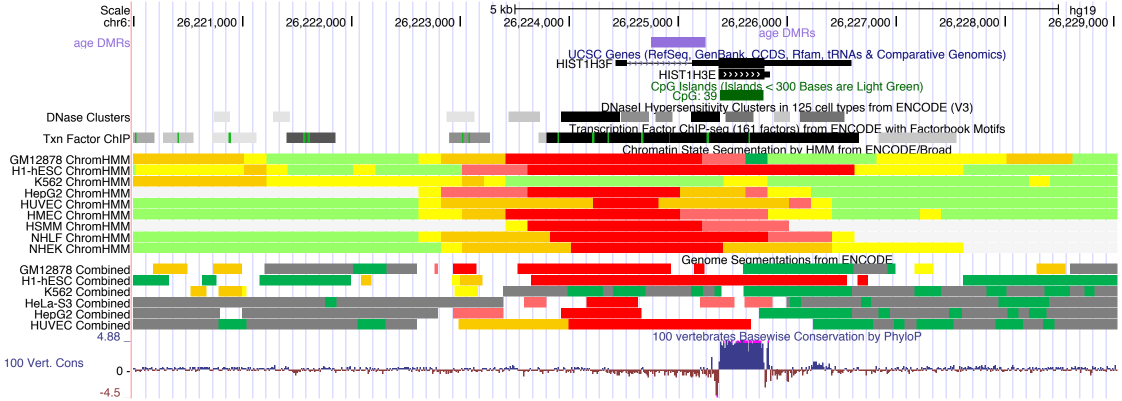

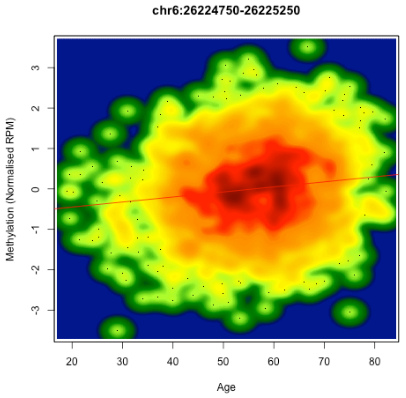

Fig S1.54: *HIST1H3F* a-DMR

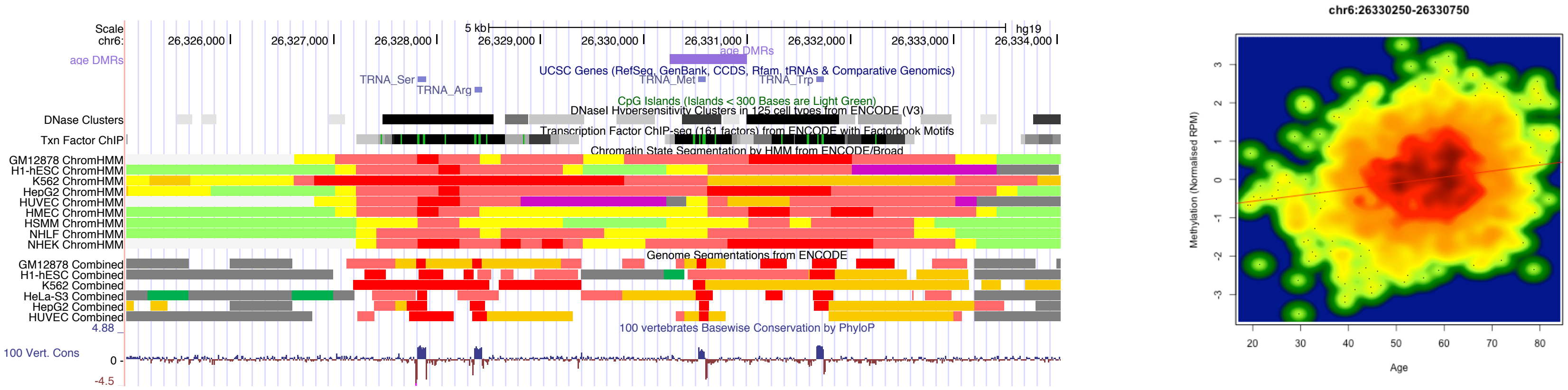

Fig S1.55: tRNA Met a-DMR

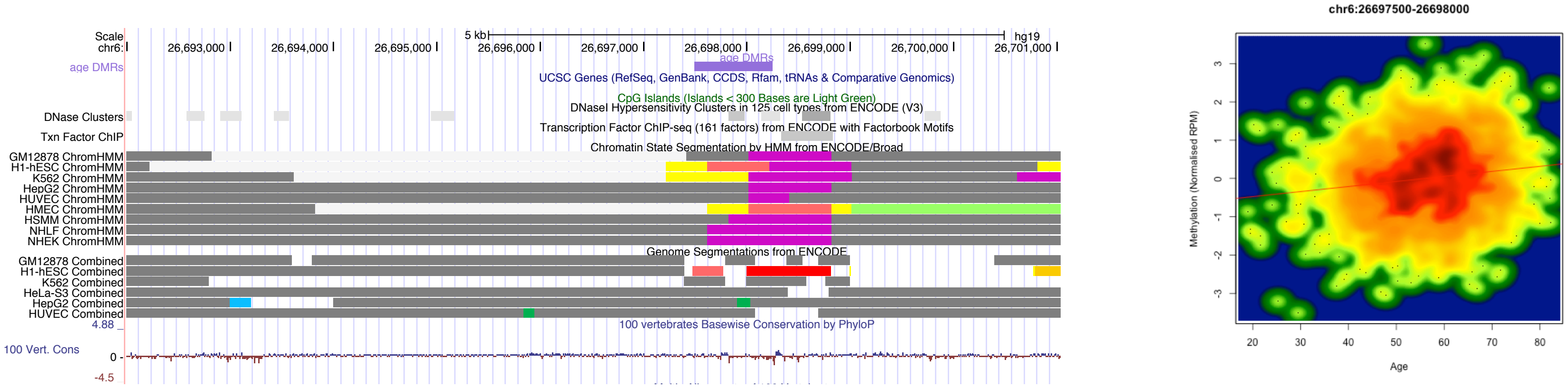

Fig S1.56: upstream *ZNF322* a-DMR

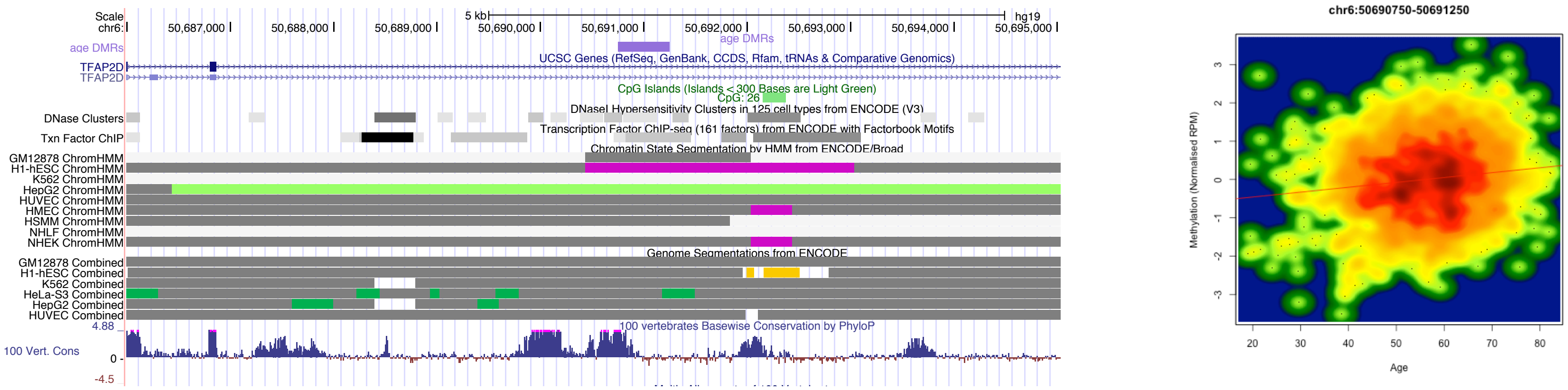

Fig S1.57: *TFAP2D* a-DMR

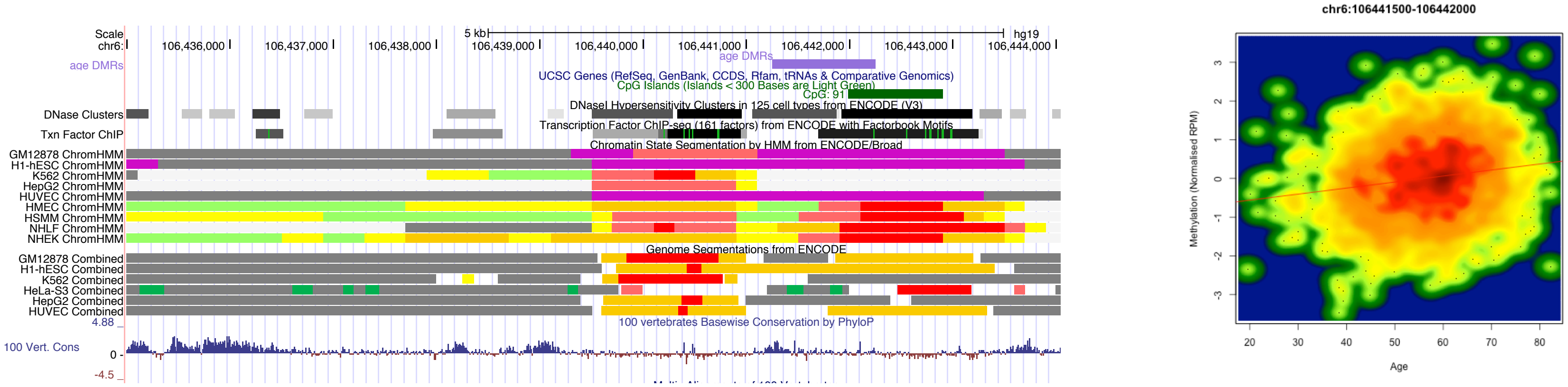

Fig S1.58: unknown chr6 a-DMR

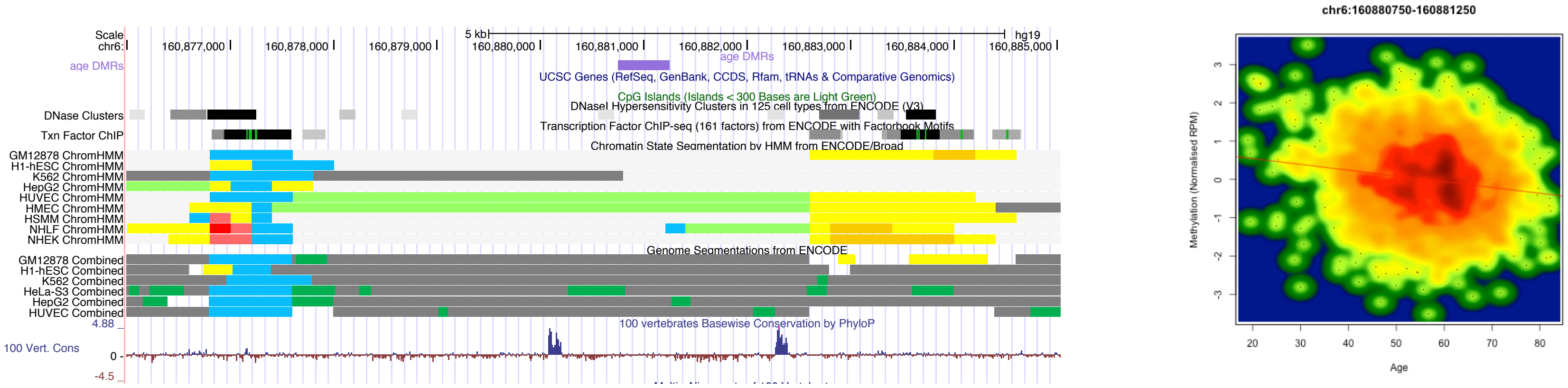

Fig S1.59: intergenic *SLC22A3 LPAL2* chr6 a-DMR

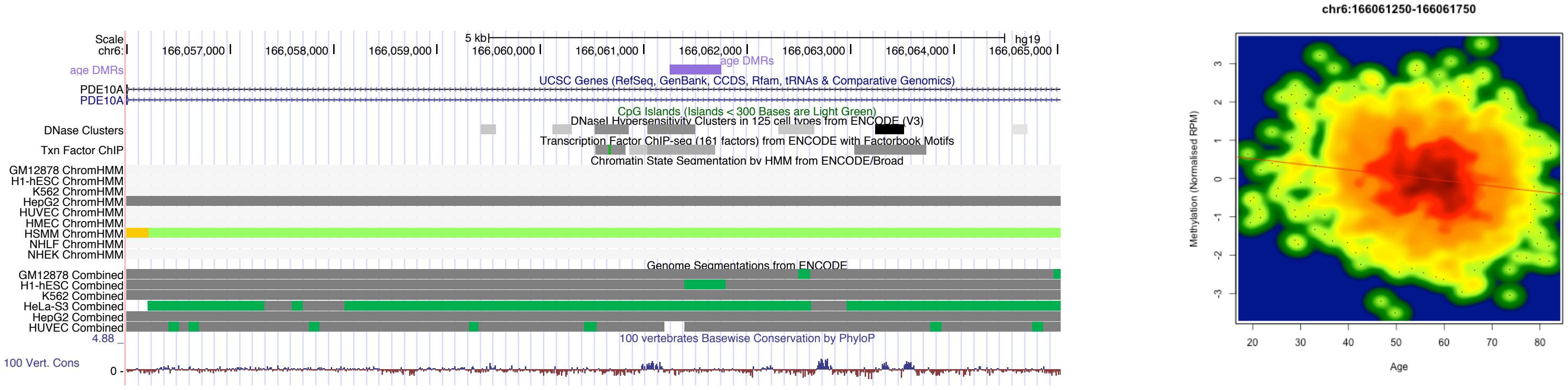

Fig S1.60: *PDE10A* a-DMR

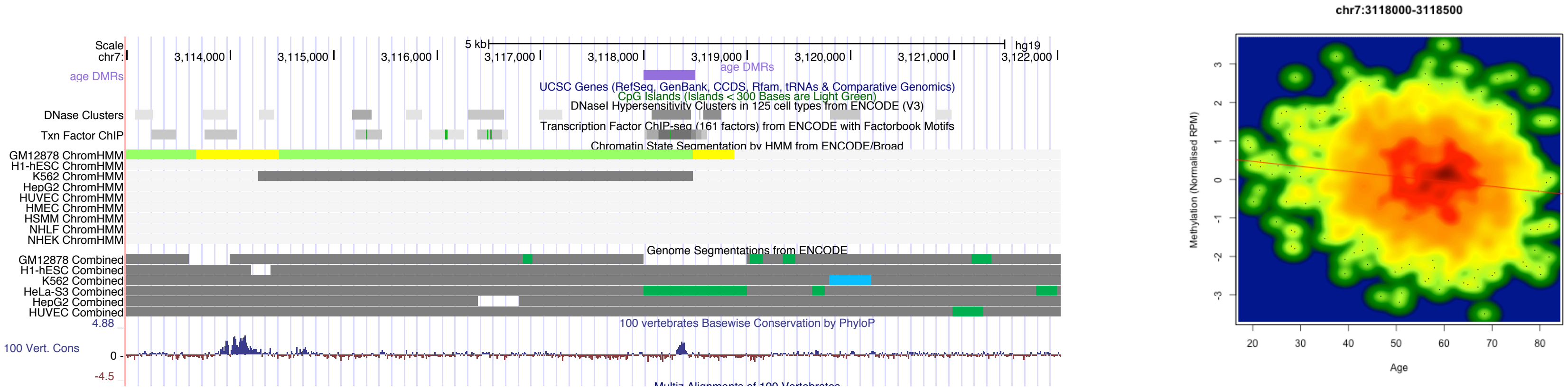

Fig S1.61: upstream *CARD11* a-DMR

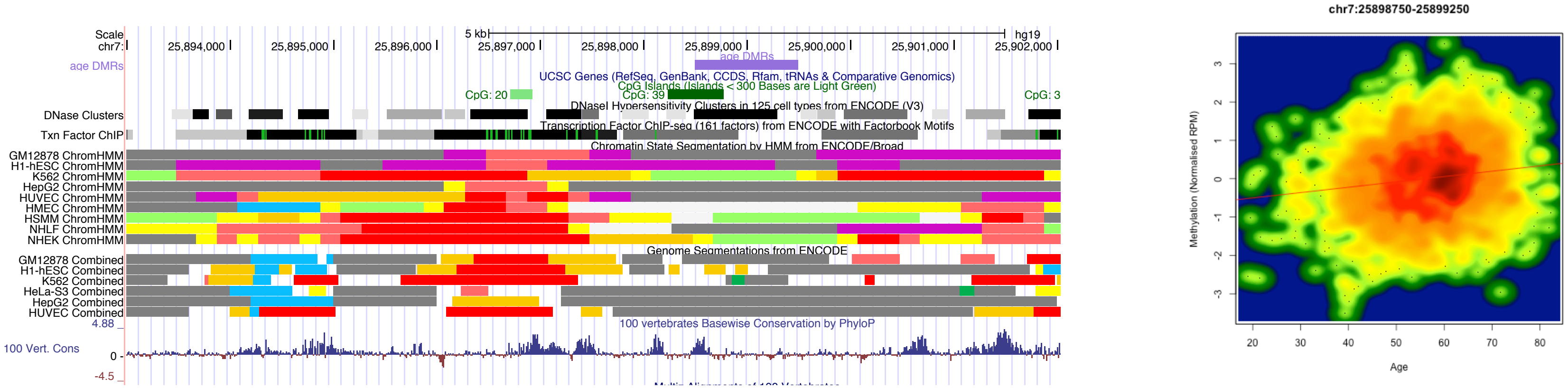

Fig S1.62: upstream *NFE2L3*

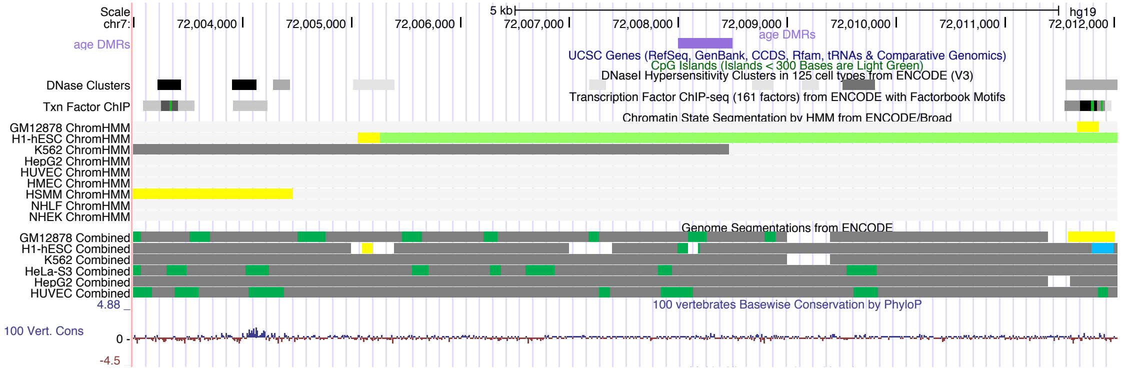

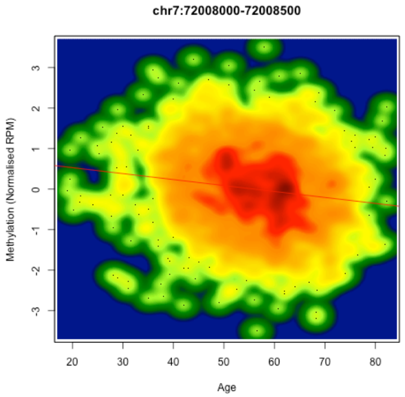

Fig S1.63: downstream *TYW1B* a-DMR

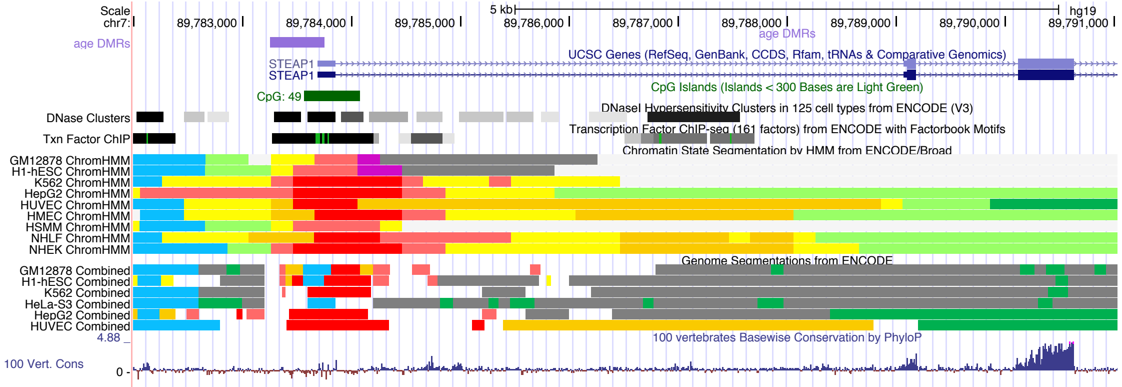

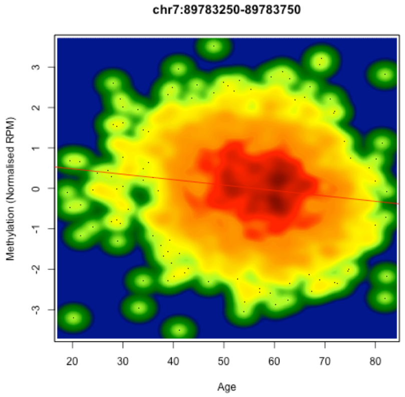

Fig S1.64: *STEAP1* a-DMR

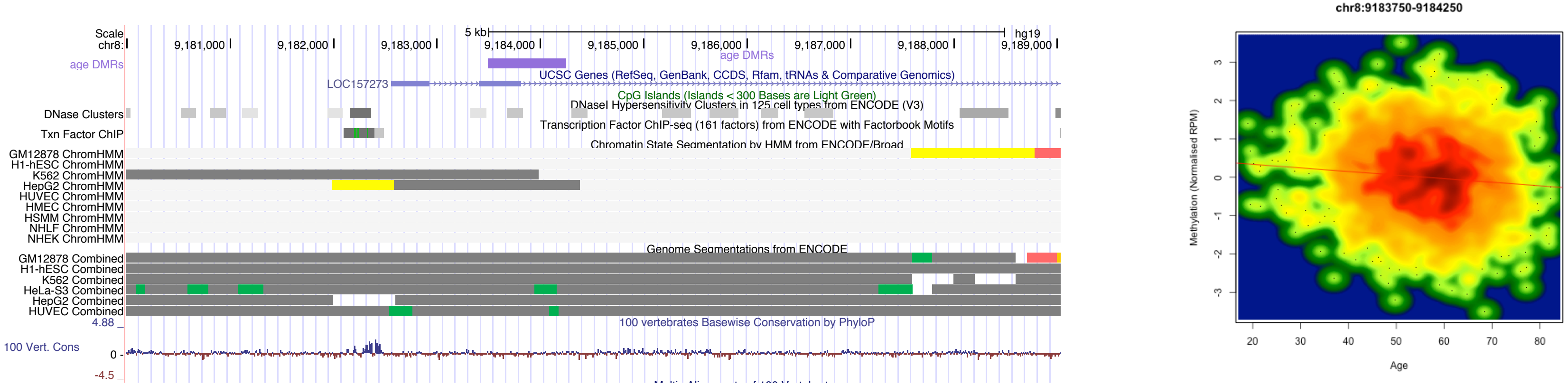

Fig S1.65: *LOC157273* a-DMR

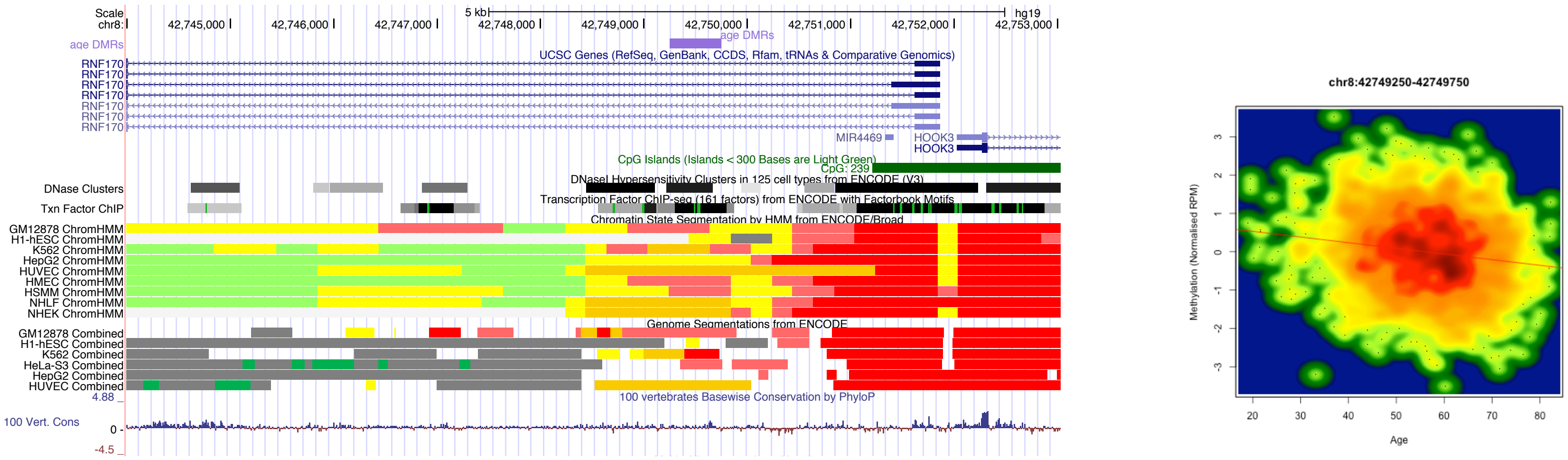

Fig S1.66: *RNF170* a-DMR

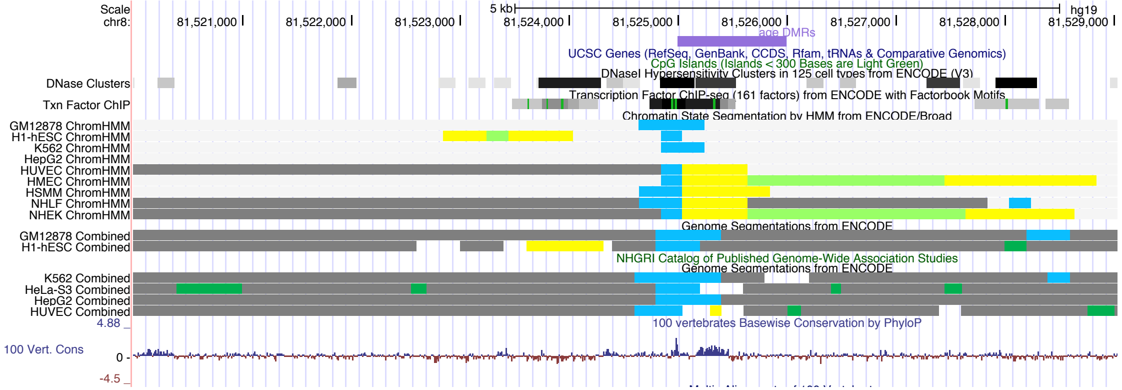

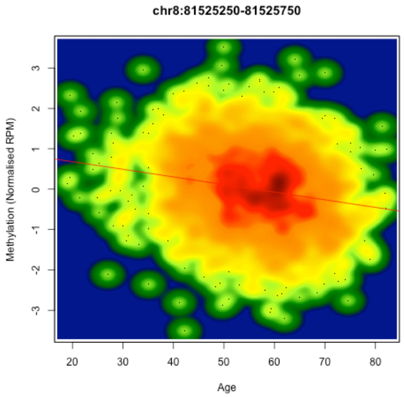

Fig S1.67: downstream *ZNF704*

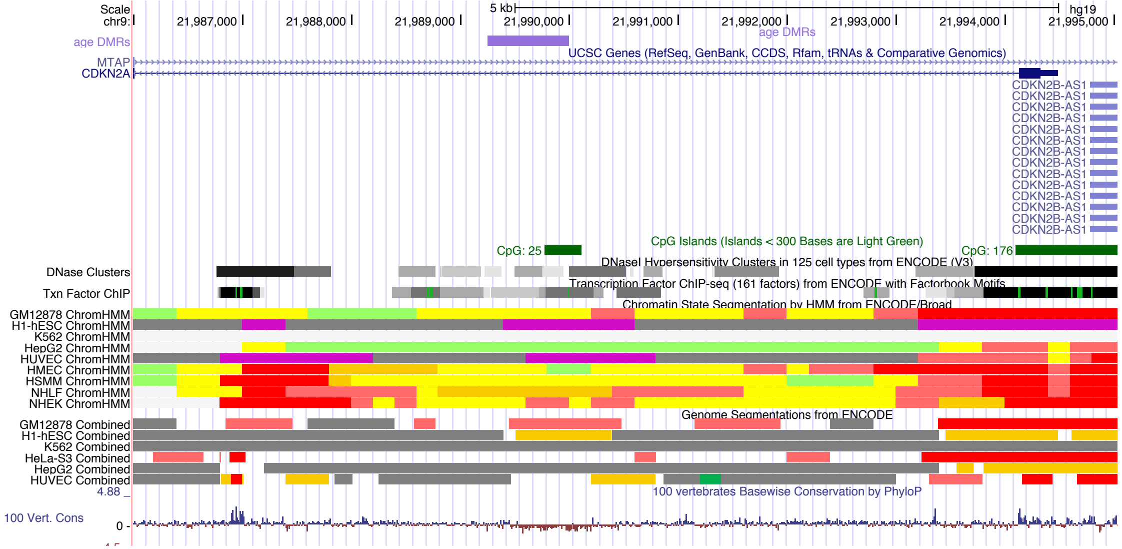

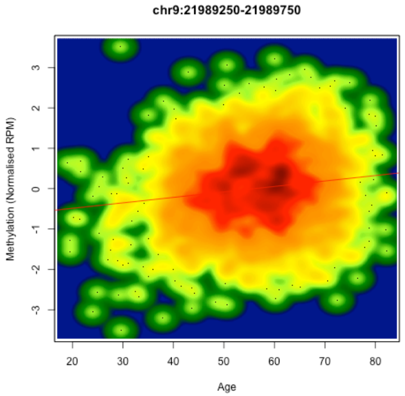

Fig S1.68: *CDKN2A* a-DMR

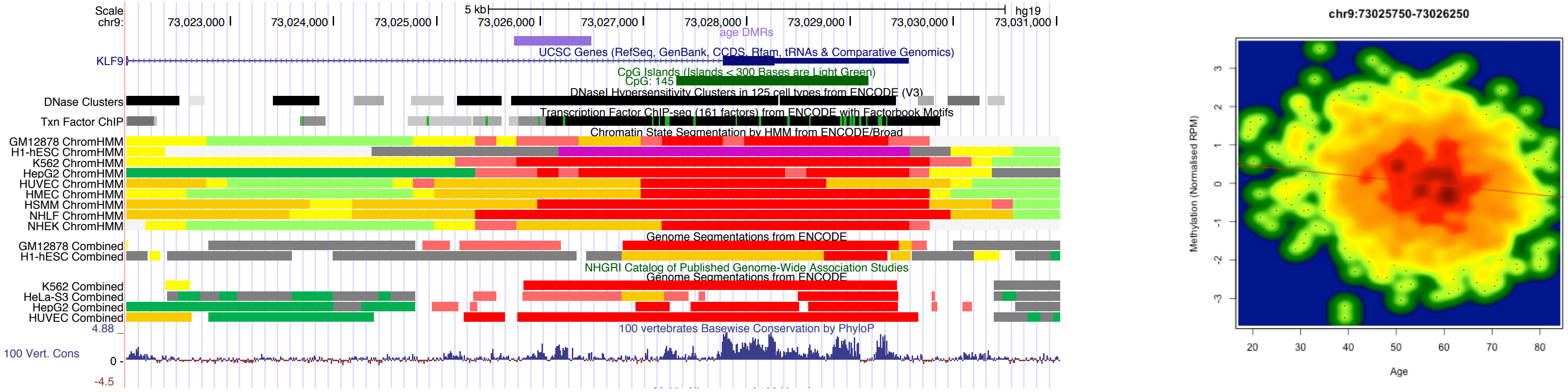

Fig S1.69: *KLF9* a-DMR

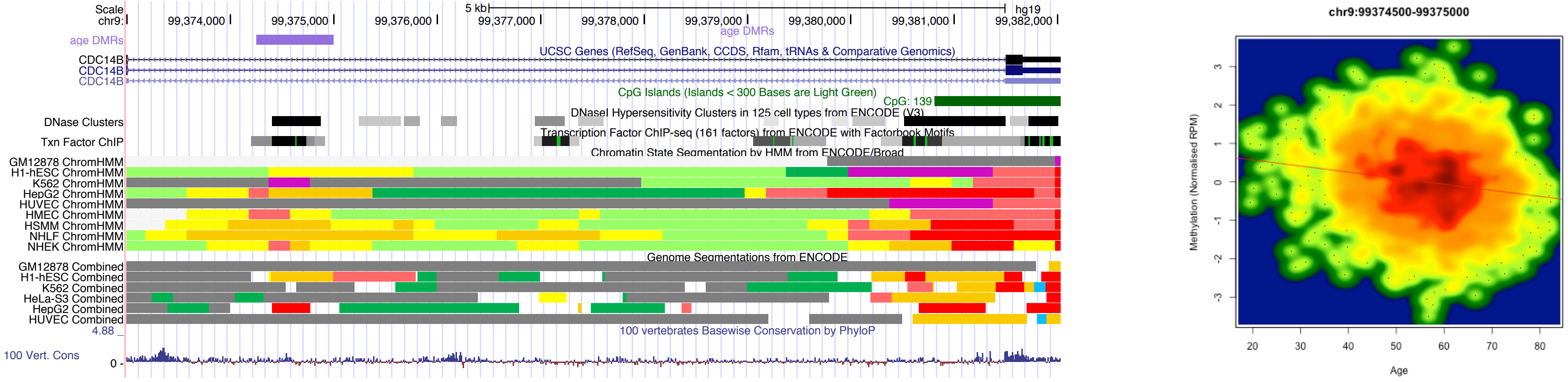

Fig S1.70: *CDC14B* a-DMR

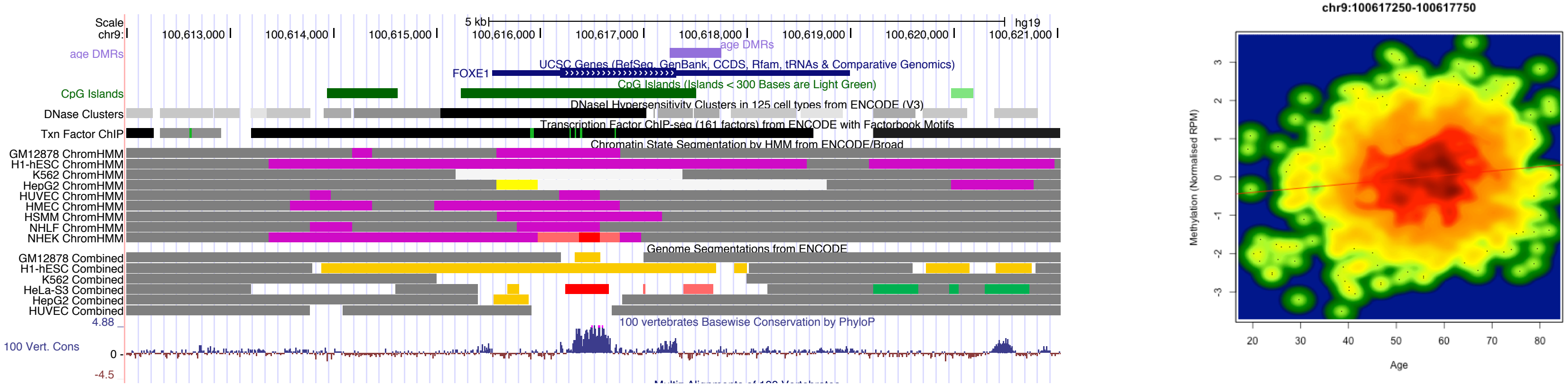

Fig S1.71: *FOXE1* a-DMR
